# Supplementary material for: A mixed-method exploration into the experience of members of the FAO/WHO International Food Safety Authorities Network (INFOSAN): study protocol
Source: BMJ Open. 2019 May 22;9(5):e027091. doi: 10.1136/bmjopen-2018-027091 (PMC6538089; doi:10.1136/bmjopen-2018-027091)
Supplement: Supplementary material 2 [file bmjopen-2018-027091supp002.pdf]

Supplementary File 2 – Information Email #2: Results from Phase 1 and invitation to attend Webinar #2 (includes Phase 2 Participant Information)

To be sent by the INFOSAN Secretariat (infosan@who.int) on behalf of the researcher (cc: c.savelli@lancaster.ac.uk)

Health & Medicine | Lancaster University

Information Email #2

A mixed-methods exploration into the experience of members of the International Food Safety Authorities Network (INFOSAN): Phase 1 results and information about Phase 2

Dear INFOSAN Member,

My name is Carmen Savelli and in August 2018 I contacted you to let you know that I was conducting this study as a student in the PhD Public Health programme at Lancaster University, Lancaster, United Kingdom. You may also know me as one of the Technical Officers working as the INFOSAN Secretariat at the World Health Organization (WHO) in Geneva, Switzerland. I am writing again to inform you of the progress made so far, including results from Phase 1 and provide some additional information for participants in Phase 2.

What is the overall study about?

The purpose of this study is to interrogate INFOSAN in order to describe and explore the experiences of members and better understand the role of the network in mitigating the burden of foodborne illness around the world. The study will examine access to and usage of the INFOSAN Community Website, explore barriers and facilitators to active participation in INFOSAN, determine perceptions about the utility of INFOSAN to mitigate foodborne illness, and scrutinize if and how participation in this network creates value for members.

The study is designed in three phases: Phase 1 is complete and has examined access and usage patterns of the INFOSAN Community Website; Phase 2 will involve the completion of an online survey; and Phase 3 will involve individual interviews conducted online using WebEx. Taking part in one phase of the study does not require your participation in the other phases.

What are the results from Phase 1?

To be inserted

Why have I been approached about Phase 2 of this study?

You have been approached because the study requires information from registered INFOSAN members.

Do I have to take part in Phase 2?

No. It is completely up to you to decide whether or not you take part. Once the survey has been submitted, it will not be possible to opt out because survey responses are anonymous.

Continued on next page…

Continued from previous page…

What will I be asked to do if I take part?
Participation in Phase 2 of the study requires the completion of an online survey of approximately 30-45 minutes which will explore questions about your experience as an INFOSAN member. All INFOSAN Members will receive a separate invitation by email(Information Email #3) to provide consent and take the survey.

Will my data be Identifiable?
No. The information you provide will be reported anonymously. Quantitative data will be reported by region so that no country-specific details will be reported. The data collected for this study will be stored securely and only the researchers conducting this study will have access to this data:

- The files on the computer will be encrypted (that is no-one other than the researcher will be able to access them) and the computer itself password protected for the duration of the study
- At the end of the study, all related electronic information will be archived for 10 years in secure encrypted storage on the Lancaster University server. At the end of this period, they will be destroyed.

For further information about how Lancaster University processes personal data for research purposes and your data rights please visit our webpage: www.lancaster.ac.uk/research/data-protection

What will happen to the results?
The results will be summarised and reported in a PhD thesis and may be submitted for publication in relevant academic or professional conferences and journals or other media including books or websites, pending necessary clearance from WHO. Only those data collected with the expressed consent of participants is utilized and reported on for the purposes of this study. INFOSAN Members will also be invited to participate in a webinar after Phase 2 to learn about the results and next steps.

Are there any risks?
There are no risks anticipated with participating in this study. However, if you experience any distress following participation you are encouraged to inform the researcher and contact the resources provided at the end of this sheet. Neither participating nor abstaining will impact your future treatment as an INFOSAN member or the technical support provided to you or your country by the WHO.

Are there any benefits to taking part?
Although you may find participating interesting, there are no direct benefits in taking part. However, some participants may find it a positive experience to reflect on their role as an INFOSAN member as well as their contributions to a safer food supply, and to participate in research which they may feel is of relevance to them, their institution, their country or the global community.

Who has reviewed the project?
This study has been reviewed and approved by the Ethics Review Committee at the World Health Organization and the Faculty of Health and Medicine Research Ethics Committee at Lancaster University.

Where can I obtain further information about the study if I need it?
If you have any questions about the study, please contact the researcher:

Carmen Savelli
c.savelli@lancaster.ac.uk
+41 22 791 3234

Or his primary supervisor in the Department of Health Research at Lancaster University:

Dr Céu Mateus
c.mateus@lancaster.ac.uk
+44 1524 593182

Or attend the upcoming webinar:

Continued on next page…

Continued from previous page…

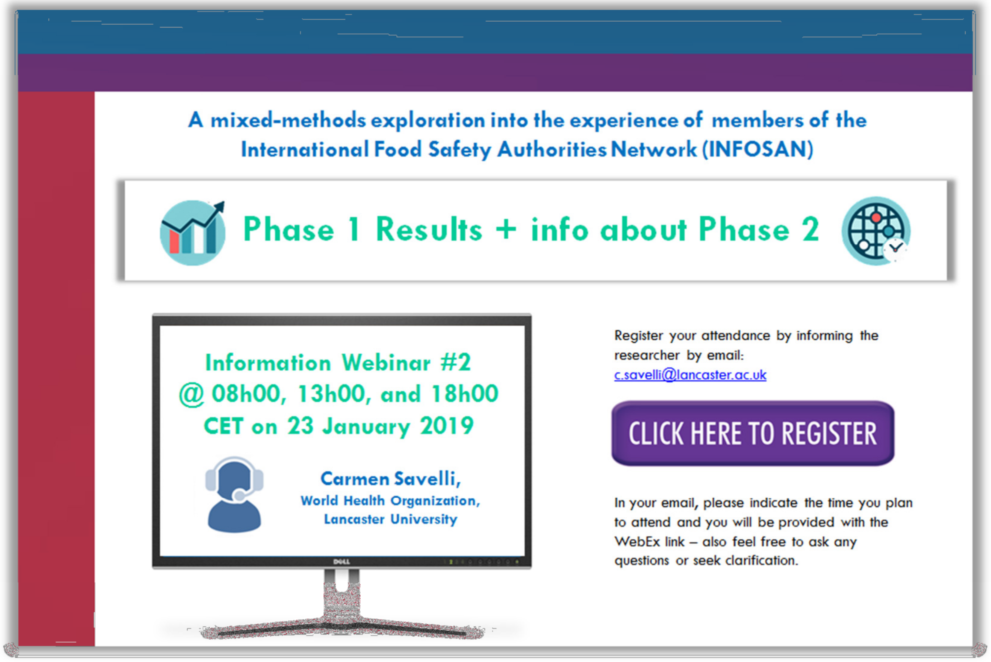

Complaints
If you wish to make a complaint or raise concerns about any aspect of this study and do not want to speak to the researcher or his supervisor, you can contact:

Professor Catherine Walshe
Head of the Division of Health Research
+44 (0)1524 510124
c.walshe@lancaster.ac.uk
Lancaster University
Lancaster
LA1 4YW

If you wish to speak to someone outside of the Public Health Doctorate Programme, you may also contact:

Professor Roger Pickup Tel: +44 (0)1524 593746
Associate Dean for Research Email: r.pickup@lancaster.ac.uk
Faculty of Health and Medicine
(Division of Biomedical and Life Sciences)
Lancaster University
Lancaster
LA1 4YG

Thank you for taking the time to read this information.

Resources in the event of distress
Should you feel distressed either as a result of taking part, or in the future, the following resource may be of assistance: https://online.supportgroups.com/

Kind regards,

Carmen Savelli
