# Supplementary material for: A mixed-method exploration into the experience of members of the FAO/WHO International Food Safety Authorities Network (INFOSAN): study protocol
Source: BMJ Open. 2019 May 22;9(5):e027091. doi: 10.1136/bmjopen-2018-027091 (PMC6538089; doi:10.1136/bmjopen-2018-027091)
Supplement: Supplementary material 3 [file bmjopen-2018-027091supp003.pdf]

Supplementary File 3 – Information Email #3: Invitation to participate in Phase 2 including consent information and link to online survey

To be sent by the INFOSAN Secretariat (infosan@who.int) on behalf of the researcher (cc: c.savelli@lancaster.ac.uk)

Health & Medicine | Lancaster University

Information Email #3

A mixed-methods exploration into the experience of members of the International Food Safety Authorities Network (INFOSAN): Invitation to participate in Phase 2

Dear INFOSAN member,

You are invited to participate in Phase 2 of our study to explore the experiences of members of the International Food Safety Authorities Network (INFOSAN).

Phase 2 of the study involves completing an online survey that should take approximately 30-45 minutes of your time. Complete background details are available in the Participant Information Sheet, shared with you previously and also attached for ease of reference.

By proceeding to the survey you confirm that:

- ✓ You have read the information sheet and understand what is expected of you within this study;
- ✓ You confirm that you understand that any responses/information you give will remain anonymous;
- ✓ Your participation is voluntary;
- ✓ You consent for the information you provide to be discussed with my supervisor at Lancaster University;
- ✓ You consent to Lancaster University keeping the anonymised data for a period of 10 years after the study has finished;
- ✓ By clicking on the button below, you consent to taking part in the current study.

If you have any questions about this survey or other aspects of the study, please do not hesitate to contact me at any time: c.savelli@lancaster.ac.uk

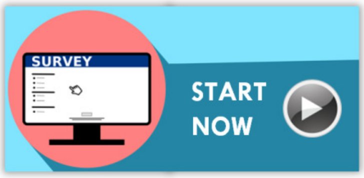

Kind regards,

Carmen Savelli
