# Supplementary figures and images for: A mixed-method exploration into the experience of members of the FAO/WHO International Food Safety Authorities Network (INFOSAN): study protocol
Source: BMJ Open. 2019 May 22;9(5):e027091. doi: 10.1136/bmjopen-2018-027091 (PMC6538089; doi:10.1136/bmjopen-2018-027091)

## Supplementary File 4 – Expansion and Adaptation of Questionnaire

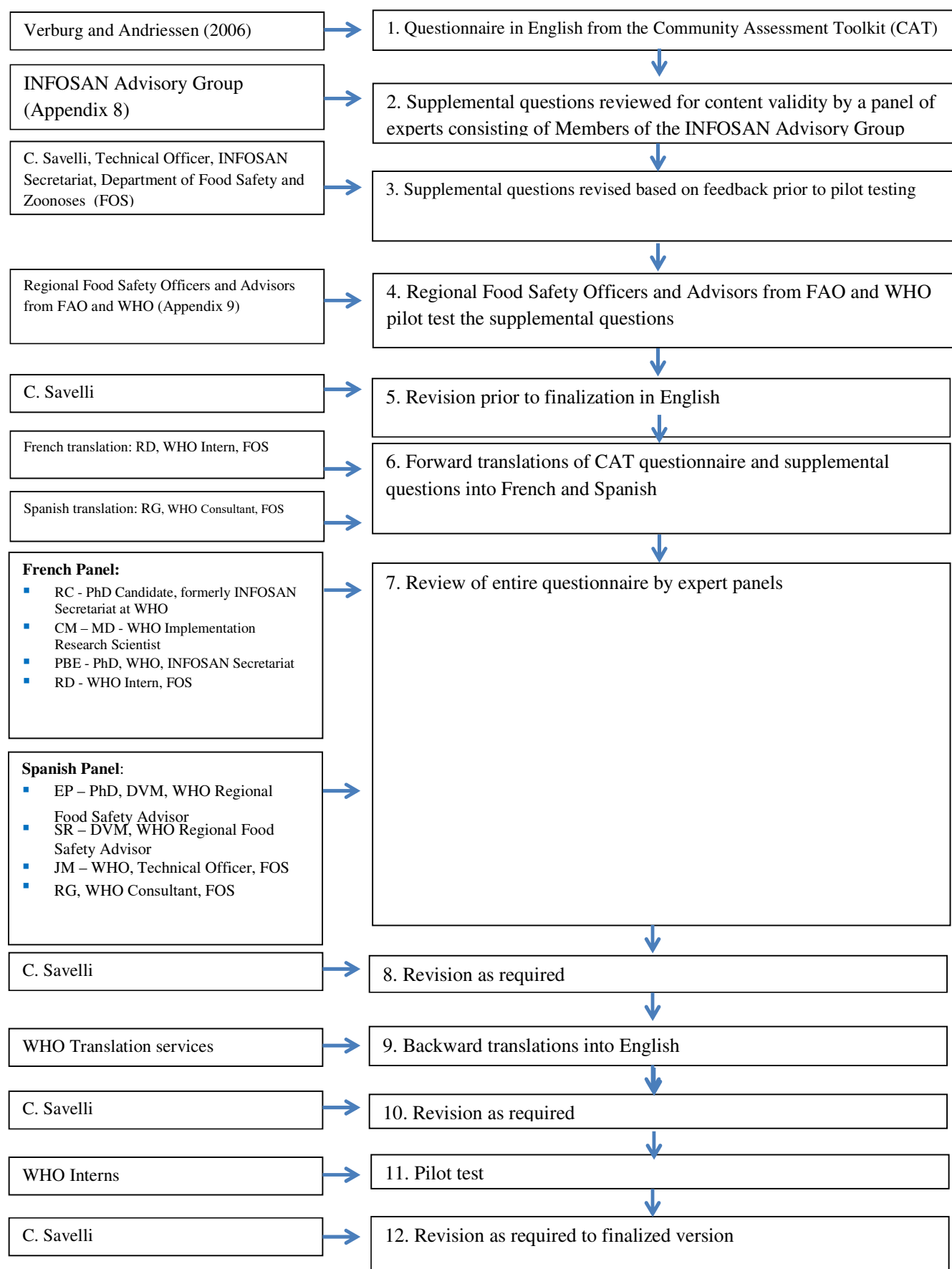

Supplement: Supplementary material 4 [file bmjopen-2018-027091supp004.pdf]
