# Supplementary material for: A mixed-method exploration into the experience of members of the FAO/WHO International Food Safety Authorities Network (INFOSAN): study protocol
Source: BMJ Open. 2019 May 22;9(5):e027091. doi: 10.1136/bmjopen-2018-027091 (PMC6538089; doi:10.1136/bmjopen-2018-027091)
Supplement: Supplementary material 6 [file bmjopen-2018-027091supp006.pdf]

Supplementary File 6 – Email to volunteers for Phase 3 that were not selected

To be sent from the researcher: c.savelli@lancaster.ac.uk

Health & Medicine | Lancaster University

Dear <<Name>>,

Many thanks for your interest in participating in Phase 3 of our study to explore the experiences of members of the International Food Safety Authorities Network (INFOSAN).

We have seen an overwhelmingly positive response to our call for volunteers to fill a limited number of interview slots. Unfortunately, due to resource restrictions including time restraints we will not be able to include you in this phase of the study.

Volunteers were selected on the basis of geographic location (maximum of two participants per WHO region) and length of membership to INFOSAN. If more than two members volunteered from the same region, the two who have been members of INFOSAN the longest were selected. This is what happened in your case.

Please be reminded that your active participation in the INFOSAN community is valued and appreciated and we hope you continue to engage in the important activities of the Network in the future.

If you have any additional information you wish to share regarding your experience as an INFOSAN member, you are always welcome to contact the INFOSAN Secretariat at WHO directly (infosan@who.int) outside of the context of this study.

Kind regards,

CSavelli

Carmen Savelli
