# Supplementary material for: A mixed-method exploration into the experience of members of the FAO/WHO International Food Safety Authorities Network (INFOSAN): study protocol
Source: BMJ Open. 2019 May 22;9(5):e027091. doi: 10.1136/bmjopen-2018-027091 (PMC6538089; doi:10.1136/bmjopen-2018-027091)
Supplement: Supplementary material 7 [file bmjopen-2018-027091supp007.pdf]

Supplementary File 7 – Consent form for volunteers participating in Phase 3

To be sent from the researcher: c.savelli@lancaster.ac.uk

Health & Medicine | Lancaster University

A mixed-methods exploration into the experience of members of the International Food Safety Authorities Network (INFOSAN): Phase 3 consent form

Many thanks for your interest in participating in Phase 3 of our study to explore the experiences of members of the International Food Safety Authorities Network (INFOSAN).

Consent Form

Before you consent to participating in the study we ask that you re-read the participant information (contained in Information Email #4 and attached for ease of reference) and mark each box below with your initials if you agree. If you have any questions or queries before signing the consent form please contact the researcher, Carmen Savelli (c.savelli@lancaster.ac.uk or +41 (0) 79 9456 320).

Initial here

1. I confirm that I have read the participant information and fully understand what is expected of me within this study ☐
2. I confirm that I have had the opportunity to ask any questions and to have them answered. ☐
3. I understand that my interview will be audio recorded and then made into an anonymised written transcript. ☐
4. I understand that audio recordings will be kept until the research project has been examined. ☐
5. I understand that my participation is voluntary and that I am free to withdraw at any time without giving any reason. ☐
6. I understand that once my data have been anonymised and incorporated into themes it might not be possible for it to be withdrawn, though every attempt will be made to extract my data, up to the point of publication. ☐
7. I understand that the information from my interview will be pooled with other participants' responses, anonymised and may be published. ☐
8. I consent to information and quotations from my interview being used in reports, conferences and training events. ☐
9. I understand that the researcher will discuss data with their supervisor as needed. ☐
10. I understand that any information I give will remain confidential and anonymous unless it is thought that there is a risk of harm to myself or others, in which case the principal investigator will need to share this information with their research supervisor. ☐ ☐
11. I consent to Lancaster University keeping written transcriptions of the interview for 10 years after the study has finished. ☐
12. I consent to take part in the above study. ☐

Name of Participant__________________ Signature____________________ Date ___________

Name of Researcher __________________Signature ____________________Date ___________
