# Supplementary material for: A mixed-method exploration into the experience of members of the FAO/WHO International Food Safety Authorities Network (INFOSAN): study protocol
Source: BMJ Open. 2019 May 22;9(5):e027091. doi: 10.1136/bmjopen-2018-027091 (PMC6538089; doi:10.1136/bmjopen-2018-027091)
Supplement: Supplementary material 8 [file bmjopen-2018-027091supp008.pdf]

Supplementary File 8 – Interview Guide for Phase 3

The semi-structured format of the interviews will be conversational in style, allowing the researcher and participant to engage in a dialogue where questions can be modified depending on responses. However, the following questions can serve to start discussions within the bounds of the four themes:

Theme 1) Use of the ICW to support network activities

Preface: The INFOSAN Community Website was launched in 2012 and all members of INFOSAN are registered on there. This online portal is the gateway through which all members can communicate with each other and with the INFOSAN Secretariat.

Questions about experiences:

- How often do you access the INFOSAN Community Website?
- What do you normally use it for?
- Do you think it is supporting the activities of INFOSAN?
- Do you think it could be improved and how?
- Do you think the INFOSAN Secretariat should be posting more frequently on the website?
- What kind of information do you think the INFOSAN Secretariat should be posting there?
- How do you think we might be able to get more members engaged on the website, e.g. involved in discussions, sharing documents, etc.?
- What new features would you like to see?
- Do you think a mobile version of the website would improve participation?

Theme 2) Barriers and facilitators to active participation in INFOSAN

Preface: INFOSAN has an important mandate, to communicate between national government authorities around the world to keep the global food supply safe. However, we know that some members are more active than others. Let’s discuss some of the potential barriers to and facilitators to participation.

Questions about experiences:

- Is limited capacity in your country a barrier to participation (i.e. Limited capacity/infrastructure dedicated to addressing food safety; insufficient funds; human resources/expertise; national food control system underdeveloped) and if so, how?
- Is lack of training a barrier (Laboratory analysis; food safety risk assessment; outbreak investigation), and if so, how?
- Does a lack of national food safety standards pose a barrier, and if so, how?
- What is coordination between national authorities like at the national level?
- Does a lack of coordination pose a barrier, and if so, how?
- Are their legal constraints that bar your participation in INFOSAN (e.g. legal implications hinder prompt information sharing; lack of food safety legislation; lack of cooperation from industry, etc.), and if so, how?
- Is food safety a priority in your country? If not, does this pose a barrier, and if so, how?

- Are their perceptions that participating in INFOSAN may have a negative impact on the economy (i.e. through damages to trade or tourism sectors)?
- Is the mandate of INFOSAN clear? If not, how could it be clarified?
- If more members understood their roles and responsibilities better, would that facilitate participations? What would be the best way to do this?
- Have you ever attended any INFOSAN training either online or in person? What did you think about the training? How could the training be improved?
- Do you think language barriers restrict active participation in INFOSAN , and if yes, how so?
- Do you have concerns over confidentiality that limit what you share with INFOSAN?
- How could these concerns be addressed to help facilitate your improved participation?
- Are there any obvious weaknesses of INFOSAN, which if rectified could significantly improve the functioning and thereby the impact of INFOSAN?

Theme 3) Perceptions of network achievements and attainment of objectives (including reducing foodborne illness)

Preface: INFOSAN aims to prevent the international spread of contaminated food and foodborne disease and strengthen food safety systems globally, by: 1) Promoting the rapid exchange of information during food safety incidents/emergencies; 2) Sharing information on important food safety issues of global interest; 3) Promoting partnership and collaboration between countries; and 4) Helping countries strengthen their capacity to manage food safety risks "from farm to table".

Questions about experiences:

- How useful has INFOSAN been in promoting the rapid exchange of information during food safety incidents/emergencies?
- Have you been involved in such exchanges? What was that like? Did you get the information you needed quickly enough to respond and implement risk management measures?
- What have your experiences been like when dealing with the INFOSAN Secretariat?
- Does INFOSAN share information on important food safety issues of global interest that you have found useful?
- Have you shared information with other network members?
- What have your experiences been like in terms of using information you received through INFOSAN or sharing information with others in the network?
- Has INFOSAN promoted partnership and collaboration between countries and networks? If yes, how so? Was it useful?
- What has been your experience when interacting with other INFOSAN members during a food safety emergency?
- Has INFOSAN helped countries strengthen their capacity to manage food safety risks "from farm to table"? If yes, how so?
- How could INFOSAN improve the way it functions in order to better achieve its objectives?

Relevance:

- To what extent are the objectives of INFOSAN still valid?

- Are the activities and outputs of INFOSAN consistent with the overall goal and the attainment of its objectives?
- Are the activities and outputs of INFOSAN consistent with the intended impacts and effects?

Effectiveness:

- To what extent have the objectives been achieved or are likely to be achieved?
- What are the major factors influencing the achievement or non-achievement of the objectives?

Efficiency:

- Were INFOSAN activities cost-efficient?
- Were objectives achieved in a timely manner?
- Were INFOSAN activities delivered in the most efficient way compared to alternatives?

Impact:

- What has been the biggest impact that INFOSAN has made?
- What real difference has INFOSAN made to members? To the safety of the global food supply? To the improvement of global food safety systems?
- How have populations' health been affected?

Sustainability:

- What are the major factors which influence the achievement or non-achievement of the sustainability of INFOSAN?

Theme 4) Assessing value of INFOSAN

Preface: INFOSAN has been in operation since 2004, and this study is the first of its kind to try and determine if the network has value to members in a robust and rigorous way.

Questions about experiences:

- What has participation in INFOSAN been a valuable experience for you? How so?
- Have you been able to connect with other food safety professionals to exchange information related to food safety?
- Has information received through INFOSAN led you to implement risk management measures in your country to prevent foodborne illness? Can you tell me about some specific events?
- Do you believe that participation in INFOSAN has reduced foodborne illness in your country? How so? How do you think this could be measured?
- Do you think participation in INFOSAN will reduce foodborne illness in your country the future? Globally?
- Does the utilization of the INFOSAN Community Website add value to the network activities?
- Do you feel like you are part of a global community? Is that something you value?

Is there anything else about your experience as an INFOSAN Member that you would like to share with me at this time?
