# Supplementary material for: A mixed-method exploration into the experience of members of the FAO/WHO International Food Safety Authorities Network (INFOSAN): study protocol
Source: BMJ Open. 2019 May 22;9(5):e027091. doi: 10.1136/bmjopen-2018-027091 (PMC6538089; doi:10.1136/bmjopen-2018-027091)
Supplement: Supplementary material 9 [file bmjopen-2018-027091supp009.pdf]

Supplementary File 9 – Information Email #5 – Results from Phase 3 and study conclusion (including invitation to attend Webinar #4)

To be sent by the INFOSAN Secretariat (infosan@who.int) on behalf of the researcher (cc: c.savelli@lancaster.ac.uk)

Health & Medicine | Lancaster University

Information Email #5

A mixed-methods exploration into the experience of members of the International Food Safety Authorities Network (INFOSAN): Phase 3 results and study conclusion

---

Dear INFOSAN Member,

My name is Carmen Savelli and in May 2018 I first contacted you to let you know that I was conducting this study as a student in the PhD Public Health programme at Lancaster University, Lancaster, United Kingdom. You may also know me as one of the Technical Officers working as the INFOSAN Secretariat at the World Health Organization (WHO) in Geneva, Switzerland. The study has now concluded.

What was the overall study about?

The purpose of this study is to interrogate INFOSAN in order to describe and explore the experiences of members and better understand the role of the network in mitigating the burden of foodborne illness around the world. The study will examine access to and usage of the INFOSAN Community Website, explore barriers and facilitators to active participation in INFOSAN, determine perceptions about the utility of INFOSAN to mitigate foodborne illness, and scrutinize if and how participation in this network creates value for members.

The study was designed in three phases: Phase 1 has examined access and usage patterns of the INFOSAN Community Website; Phase 2 involved the completion of an online survey by INFOSAN members; and Phase 3 involved individual interviews conducted online using WebEx.

What are the results from Phase 3?

To be inserted

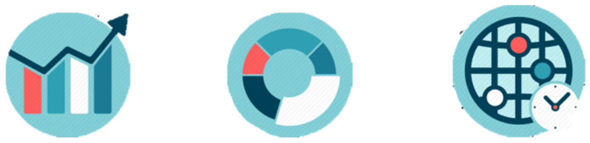

Continued on next page…

Continued from previous page…

How can I find out more about the results from Phase 3 and the overall study conclusion?
Attend the upcoming Webinar:

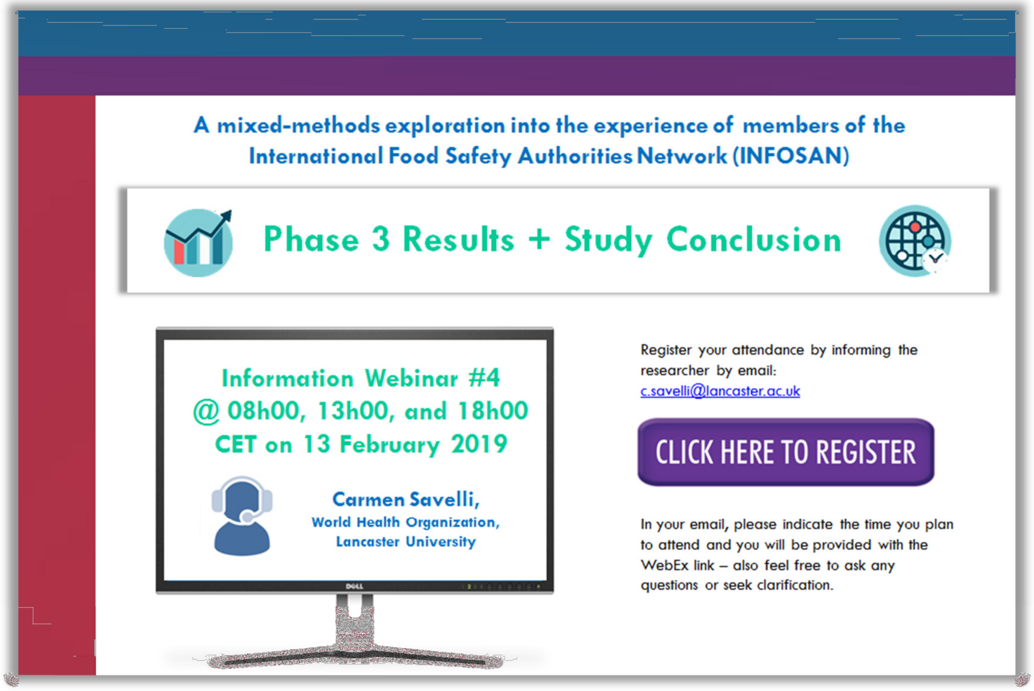

What are the next steps?

The results of this study have been shared with the INFOSAN Secretariat and other senior staff at the World Health Organization for their consideration. Improving the capacity of INFOSAN members to better prevent, detect, and respond to food safety emergencies is a priority.

Any forthcoming publications linked to the study results will be announced by email to the INFOSAN members by the INFOSAN Secretariat.

If you have any additional feedback about this study you are welcome to contact the researcher (c.savelli@lancaster.ac.uk).

If you have other feedback about INFOSAN in general that you wish to share with WHO, please contact the INFOSAN Secretariat directly (infosan@who.int).

Many thanks to all INFOSAN members who contributed time and data to this study. Your participation has been invaluable and is sincerely appreciated.

Kind regards,

C Savelli

Carmen Savelli
