# Supplementary material for: A mixed-method exploration into the experience of members of the FAO/WHO International Food Safety Authorities Network (INFOSAN): study protocol
Source: BMJ Open. 2019 May 22;9(5):e027091. doi: 10.1136/bmjopen-2018-027091 (PMC6538089; doi:10.1136/bmjopen-2018-027091)
Supplement: Author's manuscript [file bmjopen-2018-027091.draft_revisions.pdf]

BMJ Open is committed to open peer review. As part of this commitment we make the peer review history of every article we publish publicly available.

When an article is published we post the peer reviewers' comments and the authors' responses online. We also post the versions of the paper that were used during peer review. These are the versions that the peer review comments apply to.

The versions of the paper that follow are the versions that were submitted during the peer review process. They are not the versions of record or the final published versions. They should not be cited or distributed as the published version of this manuscript.

BMJ Open is an open access journal and the full, final, typeset and author-corrected version of record of the manuscript is available on our site with no access controls, subscription charges or pay-per-view fees (http://bmjopen.bmj.com).

If you have any questions on BMJ Open's open peer review process please email info.bmjopen@bmj.com

BMJ Open

A mixed-methods exploration into the experience of members of the FAO/WHO International Food Safety Authorities Network (INFOSAN): Study Protocol

Journal:	*BMJ Open*
Manuscript ID	bmjopen-2018-027091
Article Type:	Protocol
Date Submitted by the Author:	05-Oct-2018

Complete List of Authors:	Savelli, Carmen; Organisation mondiale de la Sante, Food Safety and Zoonoses; Lancaster University Faculty of Health and Medicine, Health Research<br>Mateus, Ceu; University of Lancaster, Division of Health Research

Keywords:	PUBLIC HEALTH, Information management < BIOTECHNOLOGY & BIOINFORMATICS, Information technology < BIOTECHNOLOGY & BIOINFORMATICS, World Wide Web technology < BIOTECHNOLOGY & BIOINFORMATICS

SCHOLARONE™
Manuscripts

Title: A mixed-methods exploration into the experience of members of the FAO/WHO International Food Safety Authorities Network (INFOSAN): study protocol

Authors: Carmen Joseph Savelli[1,2], Céu Mateus[2]

1) World Health Organization, Geneva, Switzerland
2) Lancaster University, Lancaster, United Kingdom

Corresponding Author: Carmen Joseph Savelli
World Health Organization
Food Safety and Zoonoses
Avenue Appia 20
1211, Geneva, Switzerland
savellic@who.int
+41 22 79 13234

Key words: international cooperation, foodborne diseases, food safety, networking, World Health Organization

Word Count: 5998

For peer review only

Abstract

Introduction: The International Food Safety Authorities Network (INFOSAN) is a global network of national food safety authorities from 188 countries, managed jointly by FAO and WHO, which facilitates the rapid exchange of information during food safety related events. The proposed research will interrogate INFOSAN in order to describe and explore the experiences of members and better understand the role of the network in mitigating the burden of foodborne illness around the world.

Methods: Examined through a community of practice (CoP) lens, a three-phase research design will combine quantitative and qualitative methods (including website analytics in phase 1, online survey administration in phase 1, and semi-structured interviews in phase 3) to elicit a broad and deep understanding of the network operation and member experiences.

Analysis: In phases 1 and 2, quantitative data collected from the INFOSAN Community Website and the online questionnaires will be analysed using descriptive summary statistics. In Phase 3, Interpretative Phenomenological Analysis (IPA) will be utilized to engage in a dialogue with study participants to explore and describe their lived experiences regarding participation in activities related to INFOSAN. An important aspect of the overall analysis will be triangulation of the information collected from each phase including quantitative indicators and qualitative value stories in order to provide a robust understanding of member experience.

Ethics: This study has undergone ethical review and has received approval from Lancaster University's Faculty of Health and Medicine Research Ethics Committee as well as the World Health Organization's Ethics Review Committee.

Dissemination: Findings from the study will be disseminated as a PhD thesis submitted to Lancaster University. In addition, results of the research shall be submitted for publication to relevant academic or professional conferences and journals or other media including books or websites.

Strengths and limitations of this study

- This study represents the first ever to explore and describe the experiences of INFOSAN members with respect to their participation in network activities as a means to improve global food safety and prevent foodborne illness.
- The mixed-method approach will strengthen the credibility of the findings and provide a more complete view and deeper understanding of the experiences of members.
- Results from this study can be utilized directly by the INFOSAN Secretariat at WHO in order to plan future interventions intended to encourage and support active participation in this important global network.
- Results from this study may also contribute new knowledge regarding the coordination of international virtual networks and communities of practice in the realm of global health.
- INFOSAN is a global network and members speak a variety languages; the main limitation of this study is that the online survey will only be available in English, French and Spanish and interviews will only be conducted in English due to limited resources of the study team.

1. Introduction

In 2015, the WHO reported estimates of the global burden of foodborne diseases for the first time. Together, 31 foodborne hazards are estimated to cause 600-million cases of foodborne disease

For peer review only

and 420,000 deaths annually, worldwide (WHO, 2015). Foodborne diseases are preventable, but ensuring a safe national food-supply requires a robust food control system and coordination among different government sectors responsible for human health, animal health, agriculture, trade and others. In addition, as a global commodity, food produced in one country can readily cause international outbreaks if contaminated food is exported abroad. Channels of communication on matters of food safety must therefore be well established within and between countries in order to prevent national and international food safety emergencies (WHO, 2014). It is for these reasons why the WHO launched INFOSAN in 2004. Today, the overall aim of INFOSAN is to halt the international spread of contaminated food, prevent foodborne disease outbreaks, and strengthen food safety systems globally (WHO, 2016). The four main objectives of INFOSAN are to: 1) Promote the rapid exchange of information during food safety related events; 2) Share information on important food safety related issues of global interest; 3) Promote partnerships and collaboration between countries, and between networks; and 4) Help countries strengthen their capacity to manage food safety emergencies. Using the INFOSAN Community Website (ICW; a secure, online portal), INFOSAN members from around the world exchange information on urgent food safety issues and emerging trends of potential global interest. The ICW also provides a virtual environment to share lessons-learned and allows members to pose questions to one another for the purpose of building knowledge related to food safety (WHO, 2016).

INFOSAN members have a common identity that is defined by a shared domain of interest. By joining the network, each has committed to taking actions that contribute to a safer global food supply by engaging in joint activities and discussions to facilitate knowledge transfer and exchange (KTE). Common responsibilities are also shared by members, as defined by the INFOSAN Secretariat. Combined, these common responsibilities and activities create a sense of community, and are undertaken with the intention of facilitating the application of best practices to improve food safety. In addition, INFOSAN members are each practitioners in their respective countries, some as food regulators, or risk analysts, or epidemiologists or other professionals. While their focus may be different, the uniting factor is that their practice, in some respect, aims to make contributions towards the reduction of foodborne illness. It is the shared domain, community, and practice which allows for

For peer review only

INFOSAN to be understood as a Community of Practice (CoP; Wenger, McDermott and Snyder, 2002). A CoP is a group of people sharing a particular concern, problem or passion for an area and who deepen their knowledge and expertise by learning from one another and interacting on a regular basis (Wenger et al., 2002). Such interactions may occur in person or through technology-mediated means, as with INFOSAN.

A growing body of research suggests that KTE can be effectively fostered within CoPs, leading to the uptake and application of best practices by individuals and teams in various sectors including health, business, and beyond (Ho et al., 2010). Multiple systematic reviews (Kothari et al., 2011, Mairs et al., 2013, Quinn et al., 2014, Barbour et al., 2018) suggest that fostering a virtual or electronic CoP among professionals in public health is useful for encouraging KTE which translates into adoption of evidence-based best practices, and by extension, improved public health. Rajic et al. (2013) have described the benefits of facilitating KTE among food safety professionals working at the intersection of agriculture and health. Together, the literature suggests that a CoP like INFOSAN, connecting food safety and public health professionals from around the world, is an appropriate tool to facilitate KTE in this area. However, while INFOSAN has been operating for nearly 15 years to facilitate the aforementioned activities among its members, it has never been characterized or examined as a functional CoP and its value, as understood from the perspective of its members, has never been determined in a systematic or rigorous way. Furthermore, a paucity of research has been conducted to investigate the attributes and effectiveness of specific tools or CoPs like INFOSAN to facilitate cross-border communication during international food safety events. To date, most of the publications mentioning such tools focus on summarising a particular incident response, rather than explicitly examining the tools that were utilised. However, such reports of international food safety events commonly conclude with recommendations to better utilise existing international networks and communication tools to improve and expedite information exchange (Einöder-Moreno et al., 2016; Guzman-Herrador et al., 2013; Inns et al., 2016; Knoblauch et al., 2015; Nygard et al., 2008; Pezzoli et al., 2008; Rebolledo et al., 2013). In addition, several published studies have specified the important role that INFOSAN has played in facilitating rapid international communication between

For peer review only

government officials that led to the timely implementation of risk management measures during a food safety emergency (Acciari et al., 2015; Gossner et al., 2009; Khardori, 2012).

3. Research Aim

The overall aim is to explore and describe the experiences of INFOSAN members with respect to their participation in network activities as a means to improve global food safety and prevent foodborne illness.

4. Research Objectives

1) Assess the functioning of INFOSAN as a community of practice by obtaining systematic insights into the characteristics, performance and opinions of members.
2) Gain a broad and deep understanding of members' perceptions of the utility of INFOSAN as a global communication tool for KTE and the prevention of foodborne illness in their respective country.
3) Determine if participation in INFOSAN creates value for members and explore the mechanisms through which this may occur.

5. Main research questions

1) How is the ICW being used to support the network activities?
2) What are the barriers and enablers to active participation in INFOSAN?
3) Do members of INFOSAN believe that participation in the network has prevented (or will prevent) foodborne illness in their country?
4) Does participation in INFOSAN create value for members and if so, through what mechanisms does this occur?

6. Methodology and methods

6.1. Philosophical underpinnings

The theoretical framework for exploring experiences in CoPs proposed by Wenger et al. (2011) is utilized for this study and has its roots in critical realism. Critical realism is a philosophical perspective that accepts the existence of stable and enduring features of reality independently of one's

For peer review only

ability to perceive them (Fade, 2004). Critical realism retains an ontological perspective where the real world exists independent of perceptions, while accepting a constructionist and relativist epistemic orientation, whereby our understanding of the world can only be measured as a sum of different perspectives (Maxwell, 2012). The importance of utilizing quantitative indicators together with qualitative narratives from members is a concept described by Wenger et al. (2011) in order to understand what kind of experiences create value within CoPs. Quantitative indicators can be readily obtained from most virtual CoPs by measuring the number of unique users logging in to a web portal over time or the number of postings within a discussion forum, for example. However, Wenger et al., (2011) suggest that relying on such indicators alone would require too many assumptions to be made to accurately find meaning in experiences and determine value. Such indicators should rather serve as a point of reference from which value-creation stories can be elicited from members directly in order to provide a more robust understanding of experience. Likewise, examining only qualitative narratives from members would ignore the opportunity to crosscheck information with indicators to determine how perceptions and actions correspond. It is this acknowledgement of multiple perspectives of reality which demonstrates congruence with critical realism. In taking this approach, discrepancies and correspondences can be observed and both grounded narratives and aspirational narratives become illuminated (where grounded narratives are those for which value-creation stories are supported by quantitative indicators and aspirational narratives are not; Wenger et al., 2011).

With an understanding of INFOSAN as a functional CoP, and with an appreciation for the critical realist perspective that underpins its examination, it is incumbent upon the researcher of the proposed study to take a mixed-method approach in order to strengthen the credibility of the findings and provide a more complete view and deeper understanding of the experiences of members. The utilization of such an approach for the investigation of experiences of online community members is strongly supported by a growing body of literature (de Laat and Lally, 2003; Roberts, 2015; Guldberg and Mackness, 2009).

6.2. Setting

The study will be conducted by the researcher from within the Department of Food Safety and Zoonoses (FOS) at the headquarters of the WHO in Geneva, Switzerland (where the INFOSAN

For peer review only - http://bmjopen.bmj.com/site/about/guidelines.xhtml

Secretariat is based), however the true setting is global since INFOSAN membership spans 188 countries.

6.3 General comments on sampling

For each phase of the research, participants will only include registered INFOSAN members. These individuals have been officially designated by their national government and are all registered on the ICW (N=500). INFOSAN membership includes both men and women in approximately equal proportions.

6.4 General comments on recruitment

Written permission to conduct the proposed research on INFOSAN has been received from the Director of FOS at the WHO, where the INFOSAN Secretariat is based (supplementary file #1). To announce the launch of the study, all members of INFOSAN will receive by email, written introductory information about the proposed research (Information Email #1; supplementary file #2), including an invitation to attend an online seminar (i.e. webinar), delivered by the researcher, to find out more information about the study and ask any questions or seek clarification. This webinar, like all future webinars discussed in this proposal, will be scheduled at three different times to allow INFOSAN members across different time zones to participate. The webinar will be delivered using the secure online conferencing tool, WebEx. The live webinars will not be recorded, but a recording will be made by the researcher alone and will be made available to INFOSAN members to view in case they would like to do so at their convenience.

6.5. Phase 1: Analysis of INFOSAN Community Website access and usage

6.5.1. Sampling, recruitment and consent

Information Email #1 will explain the three different phases of the proposed research, and remind INFOSAN members that data analysed in Phase 1 of the study will be extracted from the ICW in accordance with the Terms and Conditions of Use that each member consented to when they registered online (supplementary file #3). The relevant text within the Terms and Conditions of Use, reads as follows: "Utilization of website analytics tools or other methods will be applied periodically to summarize members' access to and usage of the INFOSAN Community Website for monitoring, evaluation and research purposes." As such, data pertaining to all members of INFOSAN with respect

For peer review only

to their access to and usage of the ICW, will be utilised in Phase 1 of the study unless a member expressly indicates their wish to be excluded. Information Email #1 (and related webinars) will ensure that INFOSAN members understand that any members not wishing to have their website access and usage data utilised for the purpose of this study will have two weeks to make this indication by email to the researcher. All those members who do not otherwise object will be recruited for Phase 1. Those who are recruited in Phase 1 will have a further two weeks to opt out of the study, after which time this will no longer be possible due to aggregation of the data.

6.5.2. Data collection and analysis

Access to the ICW is granted to the researcher in his capacity as a staff member at WHO and approval for use in this research has been granted by the Director of FOS, WHO (supplementary file #1). Data from the ICW will be collected retrospectively for the period between February 2012 (when the website was launched) and December 2018. Information from all recruited members concerning the following variables will be downloaded from the website, anonymised and exported into Microsoft Excel and SPSS for analysis: type of member, sex, country, languages spoken, government sector, primary function (i.e. risk assessment, risk communication, or risk management), and areas of scientific expertise. These data have all been automatically collected and stored in the internal website database at the time of member registration (see screenshot of online registration form in supplementary file #4). Additional data about length of membership, last access to the website, and discussion thread initiations, responses and views will also be collected and exported for analysis. Once collected, all anonymised data will be analysed using descriptive summary statistics, which will allow for stratification by a number of variables including type of member, geographic region, and length of membership. Analysing these data will provide an objective, foundational layer of information about the experiences of all members and will be triangulated with data from Phases 2 and 3 to determine if members' reported attitudes and experiences reflect their online behaviours. In addition, triangulated data will also allow for INFOSAN to be described with respect to its stage of community development according to Wenger et al (2002), taking into account its structuring characteristics as described by Dubé, Bourhis and Jacob (2006).

6.6. Phase 2: Administration of an online survey

For peer review only

6.6.1. Sampling, recruitment and consent

Upon conclusion of data collection and analysis in Phase 1, all members of INFOSAN will receive Information Email #2 (supplementary file #5) which will include indicative results from Phase 1 and an invitation to attend a webinar, delivered by the researcher, to learn more about the results from Phase 1 and to provide further details about Phase 2. Immediately following the webinar, all INFOSAN members (N=500) will receive Information Email #3 (supplementary file #6), including the invitation to participate in Phase 2 of the study and containing a link to an online survey. Only those who express consent will be recruited as participants for Phase 2. No consent form will be collected because that would compromise anonymity, however, the email will explain that by clicking on the link, the individual confirms that they have read the introductory information and understand what is expected of them as a participant in this phase of the study (full details in supplementary file #6).

6.6.2. Data collection and analysis

Recruited participants will be requested to complete an online questionnaire that will take between 30 and 45 minutes. Responses to the questionnaire are intended to provide systematic insights into the characteristics, performance and opinions of INFOSAN members and contribute to a broader understanding of their experiences. The questionnaire will consist of questions from the Community Assessment Toolkit (CAT; Verburg and Andriessen, 2006) as well as a supplemental set of questions, tailored specifically to INFOSAN members. During the development of the CAT, Verburg and Andriessen (2006) demonstrated that the methodology for its development was based on dominant theories of CoPs and group dynamics. The CAT was pilot tested and studied with seven CoPs (n=271) to enable reliability tests and scale analysis. Cronbach's alpha for the scales ranged from .59 to .91 and as the items referred to quite separate goals, Verburg and Andriessen (2006) reported these values acceptable. Utilizing the CAT in this study will enable future comparative research between CoPs that have been assessed with the same tool (e.g. Roberts, 2015). However, given the unique nature of INFOSAN and the specific objectives of this study, it is also necessary to develop a short set of supplemental questions to examine the experiences of INFOSAN members that are unique to this particular CoP. A preliminary set of supplemental questions has been inserted to the

For peer review only

appropriate sections of the CAT questionnaire in supplementary file #7. These supplemental questions will be reviewed for content validity by a panel of experts consisting members of the INFOSAN Advisory Group (supplementary file #8) since they are familiar with the constructs that the supplemental questions are designed to measure. The expert panel will judge whether the supplemental questions adequately measure the construct they are intended to assess, and whether these supplemental questions are indeed sufficient to measure the domain of interest. A Content Validity Index (CVI) will be computed for each supplementary item and items with a CVI of 0.78 or higher for three or more experts will be considered evidence of good content validity (Polit, Beck and Owen, 2007). Based on feedback from the expert panel, the supplemental questions will be revised prior to pilot testing. After incorporating any suggestions from INFOSAN Advisory Group members, FAO/WHO Regional Food Safety Advisors/Officers (supplementary file #9) will be requested to pilot test the supplemental questions. As a measure of reliability, internal consistency will be estimated using the coefficient alpha, also known as Cronbach's alpha. Determining the internal consistency will reflect the extent to which the questionnaire items are inter-correlated, or whether they are consistent in measurement of the same construct (Tsang, Royse and Terkawi, 2017).

Because the questionnaire (including the CAT questions and the supplementary questions) will be disseminated to INFOSAN members in 188 countries, it will be adapted from English into French and Spanish in order to encourage a higher response rate. The aim of the adaptation process is to achieve different language versions of the English instrument that are conceptually equivalent in both French and Spanish. The instrument should be equally natural and acceptable and should practically perform in the same way, with a focus on cross-cultural and conceptual equivalence, rather than on linguistic/literal equivalence. A well-established method to achieve this goal is to use forward-translations and back-translations (WHO, 2018). This method has been refined over the course of several WHO studies to result in the following process which will be undertaken to adapt the questionnaires into French and Spanish (WHO, 2018). First, one native Spanish speaker and one native French speaker from the Department of Food Safety and Zoonoses at WHO will conduct forward translations of the entire questionnaire. These translators will be health professionals, familiar with the terminology of the area covered by the instrument. Next, two bilingual expert panels will be

For peer review only

convened to identify and resolve the inadequate expressions/concepts of the translation, as well as any discrepancies between the forward translation and the English versions of the questions. Next, using the same approach as outlined in the first step, the questionnaire will then be translated back to English by an independent translator (from central translation services at WHO), whose mother tongue is English and who has no knowledge of the questionnaire. As in the forward-translation, emphasis in the back-translation should be on conceptual and cultural equivalence and not linguistic equivalence. Discrepancies should be discussed with the researcher and further work (forward translations, discussion by the bilingual expert panel, etc.) will be iterated as many times as needed until a satisfactory version is reached. This version will then be pre-tested with a group of 20 interns at WHO including 10 native French speakers and 10 native Spanish speakers. A call for volunteers will be disseminated through the WHO intern mailing list. Pre-test respondents will be administered the questionnaire and then systematically debriefed. This debriefing will ask respondents what they thought the questions were asking, whether they could repeat the questions in their own words, and what came to their minds when they heard a particular phrase or term. The debriefing will take the form of a focus group, organized at WHO. The adaptation process followed will be traceable through a number of documents including: 1) initial forward versions; 2) a summary of recommendations by the expert panels; 3) the back-translations; 4) a summary of problems found during the pre-testing of the instrument and the modifications proposed; 5) the final version; and 6) a description of the samples used in this process (i.e. the composition of the expert panel and the pre-test respondent samples). The process of expanding and adapting the questionnaire is summarized in supplementary file #10.

Quantitative data collected from the online questionnaires will be analysed using descriptive summary statistics, allowing for stratification by a number of variables including type of member, geographic region, length of membership, etc. A variety of techniques for univariate, bivariate and multivariate analysis using SPSS will be employed in order to examine patterns and relationships between variables. Depending on the response rate to the survey, the researcher may need to adjust for non-response bias in order to generalise the results to the entire network (Bryman, 2012). The researcher is a native English speaker with a working knowledge of French and basic knowledge of

For peer review only

Spanish. As such, for the very few instances where an open text response is an open, answers provided in French or Spanish will be translated by the researcher with the aid of Google translate, if necessary.

6.7. Phase 3: Semi-structured interviews

6.7.1. Sampling, recruitment and consent

Upon conclusion of data collection and analysis in Phase 2, all members of INFOSAN will receive Information Email #4 (supplementary file #11) which will include indicative results from Phase 2 and an invitation to attend a webinar, delivered by the researcher, to learn more about the results from Phase 2 and to provide further details about Phase 3. The email and webinar will also include a request that members interested in participating in Phase 3 indicate this by emailing the researcher. The online survey in Phase 2 will also serve as a recruitment tool for Phase 3 since a concluding statement on the survey will indicate that members interested in participating in Phase 3 of the research should contact the researcher separately by email.

A minimum of six and a maximum of 12 participants will be included in the study sample, aiming for two participants from each of the six geographic regions delineated by WHO. The purpose is not to be representative of the entire network, however, because INFOSAN is global, including participants from each region may reveal a richer pool of experience than if all members were selected from a single region. In addition, a sample size within this range should allow for the examination of similarities and differences between individuals, without producing an overwhelmingly large amount of qualitative data that cannot be managed within the confines of the study timeline. This sample of participants will be restricted to those INFOSAN members who have been registered members for a minimum of two years at the time of their interview to ensure they have a reasonable level of experience with the network from which to draw. The sample will be limited to those members who speak English due to limited funding for research conduct (including for translation and interpretation) and limited time for collecting and analysing data in other languages. In the event that at least one member per WHO region does not volunteer after receiving Information Email #4, follow-up emails will be sent to those members from the regions where volunteers are still needed, to indicate as such.

For peer review only

If more than two people from a single WHO region indicate their interest in participating in Phase 3, the two people who have been members the longest will be selected and notified by email from the researcher, providing they are not from the same country**.** Any individuals who volunteer for Phase 3 but who are not selected will be emailed individually by the researcher and provided with an explanation about how the selection was made (supplementary file #12).

Prior to commencing their interview, INFOSAN members who volunteer and are recruited for Phase 3 of the study will need to have read, signed, and returned by email to the researcher, the Phase 3 consent form as detailed in supplementary file #13. Immediately before the interview, the researcher will ensure the participants are aware they can choose to withdraw from the study at any time, for any reason, before or during their interview and withdraw their data up to two weeks after their interview.

6.7.2. Data Collection and Analysis

Recruited participants will be requested to participate in a semi-structured interview conducted online using the secure tool, WebEx, because participants are anticipated to be located in various countries around the world (no face-to-face interviews will be conducted). The interviews will be scheduled to last between 45 minutes and one hour. The same four themes explored in Phase 2 will be explored during the interviews, and the discussion will focus on answering the related questions of 'how?' and 'why?' instead of just 'what?' as elaborated in the interview guide in supplementary file #14 (results from Phase 1 and/or 2 may lead to the addition or deletion of certain questions from the current interview guide). The researcher will follow Interpretative Phenomenological Analysis (IPA) to engage in a dialogue with study participants to explore and describe their lived experiences regarding participation in activities related to INFOSAN. Such a method requires a flexible data collection instrument (Smith et al., 2009) and therefore, the semi-structured format of the interviews will be conversational in style, allowing the researcher and participant to engage in a dialogue where questions can be modified depending on responses. This format also enables the researcher to prompt further elaboration in certain areas of interest identified by participants, allowing for more flexibility than a structured interview (Smith et al., 2009). The interviews will be audio recorded and then transcribed by the researcher. Audio recordings will be made using WebEx and immediately downloaded, password protected and encrypted on a laptop. Audio recorded data will be anonymised

For peer review only

as far as is possible (given the nature of audio data) by saving the file with an de-identified tag (e.g Participant #1 Region X). Prior to recording, the researcher will remind the participant that they can refrain from using names of people and places to the extent possible when answering questions, to assist with anonymization. Once recording has started, the researcher will not use the participant's name during the interview. Transcripts will be anonymized by replacing identifying names of people or places with a de-identified tag (e.g Participant #1 Region X). Analysis will involve multiple readings of the interview transcripts, coupled with note taking, followed by transformation of notes into emergent themes, and finally, seeking relationships and clustering themes (Smith et al., 2009).

An important aspect of the overall analysis will be triangulation of the information collected from each phase including quantitative indicators and qualitative value stories. Anonymised information and quotations from participant interviews will be reported, representing the limits to confidentiality. The combined strengths of quantitative and qualitative methods can contribute to improved study validity, credibility, and overall integrity and provide a broad and deep understanding of members' experiences (Bryman, 2012; Allsop, 2013; Cresswell, 2009). The indicative results from Phase 3, along with a triangulated analysis of data from all three phases will be communicated to INFOSAN members through Information Email #5 (supplementary file #15) and a final webinar, delivered by the researcher.

7. Patient and Public Involvement

Patients were not involved in the design of this study. The first author has had conversations with several INFOSAN members in finalizing this study design. These conversations have followed a presentation of the research design at a regional meeting of INFOSAN members in Miami, USA in November 2017 where INFOSAN members from approximately 30 countries were present. In addition, a more detailed presentation of the study design was delivered at a meeting of 8 INFOSAN members in Geneva, Switzerland in December 2017, where further discussion has contributed to the finalization of the study design, including the overall aim, objectives and research questions. The questionnaire will also be vetted by several target participants (selected because they are also part of the INFOSAN Advisory Group) as described in supplementary file #10. The results of each phase of

For peer review only

the study will be communicated to study participants via email and through webinars delivered by the first author.

8. – Ethical issues

8.1. – Data management and storage

In accordance with the General Data Protection Regulation (GDPR) and the (UK) Data Protection Act 2018, all electronic data will be anonymised and stored onto encrypted password-protected storage devices, including data keys and a laptop computer) provided by the WHO. These encrypted files will utilise meaningful file names and version numbers and will be accompanied by a Readme file. Personal identifiers will be kept separately from anonymised data in either encrypted computer storage (for electronic files) or in a locked filing cabinet (for paper files) and destroyed following the completion and acceptance of the PhD thesis. Only the researcher and his supervisors will have access to the anonymised data for the duration of the study. Electronic copies of transcripts will be transferred in person by the researcher from WHO headquarters to Lancaster University on encrypted, password-protected storage devices and then archived for 10 years in secure encrypted storage on the Lancaster University server, after which time they will be destroyed. Storage and destruction of data will be done by Lancaster University's designated Data Protection Officer.

8.2. – Conducting insider research

By the nature of this work-based research project, the researcher is an insider, investigating an issue that examines, in broad terms, the operation of an organizational programme. The researcher is therefore an agent of his organization as a technical officer at WHO and also an agent of Lancaster University as a PhD student. As such, the ethical considerations for the design of this research project have been carefully made from the insider researcher perspective.

In addition to approval being granted by the Director of FOS to conduct this research, the proposed study has been subject to scrutiny and approval by the Faculty of Health and Medicine Research Ethics Committee at Lancaster University and the WHO Research Ethics Board before it could commence. This process involved technical review by an external scientific committee of experts (supplementary file #16). In addition, the conduct of the research will be governed by the

For peer review only

WHO Code of Ethics and Professional Conduct and the WHO Code of Conduct for Responsible Research which both emphasise the need for all research to be conducted with integrity, accountability, independence, impartiality, respect, and professional commitment.

While there are several positive aspects to being an insider researcher (e.g. informed perspective, ability to implement study recommendations directly, etc.), the potential conflicts of interest must be carefully considered, acknowledged and addressed. As members of INFOSAN are familiar with the researcher in his role at WHO, he will need to ensure transparency and clarity that the proposed research is being conducted as part of a PhD study. It will also be necessary to ensure that despite having access to additional data or information, that only those data collected with the expressed consent of participants is utilised and reported on for the purposes of this study. INFOSAN members must be assured that neither their participation nor abstention will impact their future treatment as an INFOSAN member or the technical support provided to them or their agency by the WHO.

Acknowledging the role of the researcher as an insider is congruent with the methodology used to conduct this research on INFOSAN through a CoP lens. IPA as a research methodology has its theoretical roots in critical realism (Fade, 2004) and is concerned with carefully detailing the lived experience of individuals. As explained by Guldberg and Mackness (2009), utilizing IPA to understand experience aligns with a CoP lens since the focus of analysis is on the interpretations of members and the values they attribute to them. In addition, IPA acknowledges and embraces the role of the researcher's interpretation and understanding of members' lived experiences. The researcher, through his insider role on the INFOSAN Secretariat, plays an important part in connecting, communicating and facilitating interaction among members, and therefore his familiarity with the participants and ability to provide expertise in the interpretation and understanding of members' experiences should be considered an asset.

While the role of insider researcher can present some potential conflicts of interest, transparency in process, due permissions from senior WHO staff, and assurances given to INFOSAN members, should ensure this research is conducted to the highest ethical standard. In addition, several techniques well-known to insider researchers will be employed to aid in accurately understanding and

For peer review only

documenting the experiences of INFOSAN members, including the practice of reflexivity. The practice of reflexivity will involve active engagement of the self and questioning of the researcher's own perceptions in order to expose their contextualized nature (Green, 2014). A diary will be kept by the researcher during data collection and analysis to document reflexivity including predictions of outcomes for each phase of the study and how they relate to one another.

9. Dissemination plans

The researcher will disseminate findings from the study in the following ways: 1) via webinars, open to all INFOSAN members following each of the three phases of the study; 2) as an oral presentation to staff within FOS, WHO; and 3) as a PhD thesis submitted to Lancaster University. In addition, results of the research shall be submitted for publication to relevant academic or professional conferences and journals or other media including books or websites.

Authors' contributions: CS conceived the original idea, designed the study, drafted the manuscript and approved the final document. CM drafted the manuscript and approved the final document.

Funding statement: This study is funded by the World Health Organization.

Competing interests: None

References

Acciari, V., Iannetti, L., Gattuso, A., Sonnessa, M., Scavia, G., Montagna, C., et al. (2015). Tracing sources of Listeria contamination in traditional Italian cheese associated with a US outbreak: investigations in Italy. *Epidemiology And Infection*, *144*(13), 2719-2727. http://dx.doi.org/10.1017/s095026881500254x

Allsop, J. (2013). *Researching health: qualitative, quantitative and mixed methods*. Sage.

Barbour, L., Armstrong, R., Condron, P., & Palermo, C. (2018). Communities of practice to improve public health outcomes: a systematic review. Journal Of Knowledge Management, 22(2), 326-343. doi: 10.1108/jkm-03-2017-0111

Bryman, A. (2012). *Social research methods (4th Edition)*. Oxford: Oxford University Press.

Creswell, JW. (2009). *Research Design: Qualitative, Quantitative and Mixed Methods Approaches*. Sage.

de Laat, M., & Lally, V. (2003). Complexity, theory and praxis: Researching collaborative learning and tutoring processes in a networked learning community. *Instructional Science*, 31, pp. 7-39.

For peer review only

Dube, L., Bourhis, A., & Jacob, R. (2006). Towards a Typology of Virtual Communities of Practice. Interdisciplinary Journal Of Information, Knowledge, And Management, 1, 069-093. doi: 10.28945/115

Einöder-Moreno, M., Lange, H., Grepp, M., Osborg, E., Vainio, K., & Vold, L. (2016). Non-heat-treated frozen raspberries the most likely vehicle of a norovirus outbreak in Oslo, Norway, November 2013. *Epidemiology And Infection*, *144*(13), 2765-2772. http://dx.doi.org/10.1017/s0950268816000194

Fade, S. (2004). Using interpretative phenomenological analysis for public health nutrition and dietetic research: a practical guide. *Proceedings Of The Nutrition Society*, *63*(04), 647-653. http://dx.doi.org/10.1079/pns2004398

Gossner, C., Schlundt, J., Ben Embarek, P., Hird, S., Lo-Fo-Wong, D., & Beltran, J. et al. (2009). The Melamine Incident: Implications for International Food and Feed Safety. *Environmental Health Perspectives*, *117*(12), 1803-1808. http://dx.doi.org/10.1289/ehp.0900949

Greene, M. (2014). On the inside looking in: Methodological insights and challenges in conducting qualitative insider research. *The Qualitative Report*, 19 (15), 1-13.

Guldberg, K., & Mackness, J. (2009). Foundations of communities of practice: enablers and barriers to participation. *Journal Of Computer Assisted Learning*, *25*(6), 528-538. http://dx.doi.org/10.1111/j.1365-2729.2009.00327.x

Guzman-Herrador, B., Nilsen, E., Cudjoe, K., Jensvoll, L., Kvamme, J., & Lindegård Aanstad, A. et al. (2013). A Shigella sonnei outbreak traced to imported basil – the importance of good typing tools and produce traceability systems, Norway, 2011. *Eurosurveillance*, *18*(49), 20650. http://dx.doi.org/10.2807/1560-7917.es2013.18.49.20650

Ho, K., Jarvis-Selinger, S., Norman, C., Li, L., Olatunbosun, T., Cressman, C., & Nguyen, A. (2010). Electronic communities of practice: Guidelines from a project. *Journal Of Continuing Education In The Health Professions*, *30*(2), 139-143. http://dx.doi.org/10.1002/chp.20071

Inns, T., Ashton, P., Herrera-Leon, S., Lighthill, J., Foulkes, S., & Jombart, T. et al. (2016). Prospective use of whole genome sequencing (WGS) detected a multi-country outbreak of Salmonella Enteritidis. *Epidemiology And Infection*, *145*(02), 289-298. http://dx.doi.org/10.1017/s0950268816001941

Khardori, N. (2012). A Multistate Outbreak of Hepatitis A Associated With Semidried Tomatoes in Australia, 2009. *Yearbook Of Medicine*, *2012*, 101-102. http://dx.doi.org/10.1016/j.ymed.2012.08.004

Knoblauch, A., Bratschi, M., Zuske, M., Althaus, D., Stephan, R., & Hächler, H. et al. (2015). Cross-border outbreak of Salmonella enterica ssp. enterica serovar Bovismorbificans: multiple approaches for an outbreak investigation in Germany and Switzerland. *Swiss Medical Weekly*. http://dx.doi.org/10.4414/smw.2015.14182

Kothari, A., Hovanec, N., Hastie, R., & Sibbald, S. (2011). Lessons from the business sector for successful knowledge management in health care: A systematic review. *BMC Health Services Research*, *11*(1). http://dx.doi.org/10.1186/1472-6963-11-173

Mairs, K., McNeil, H., McLeod, J., Prorok, J., & Stolee, P. (2013). Online strategies to facilitate health-related knowledge transfer: a systematic search and review. *Health Information & Libraries Journal*, *30*(4), 261-277. http://dx.doi.org/10.1111/hir.12048

For peer review only

Maxwell, J. (2012). *A realist approach for qualitative research*. Thousand Oaks, CA: SAGE.

Nygård, K., Lassen, J., Vold, L., Andersson, Y., Fisher, I., & Löfdahl, S. et al. (2008). Outbreak of Salmonella Thompson Infections Linked to Imported Rucola Lettuce. *Foodborne Pathogens And Disease*, *5*(2), 165-173. http://dx.doi.org/10.1089/fpd.2007.0053

Pezzoli, L., Elson, R., Little, C., Yip, H., Fisher, I., & Yishai, R. et al. (2008). Packed with Salmonella—Investigation of an International Outbreak of Salmonella Senftenberg Infection Linked to Contamination of Prepacked Basil in 2007. *Foodborne Pathogens And Disease*, *5*(5), 661-668. http://dx.doi.org/10.1089/fpd.2008.0103

Polit, D., Beck, C., & Owen, S. (2007). Is the CVI an acceptable indicator of content validity? Appraisal and recommendations. *Research In Nursing & Health*, *30*(4), 459-467. http://dx.doi.org/10.1002/nur.20199

Quinn, E., Huckel-Schneider, C., Campbell, D., Seale, H., & Milat, A. (2014). How can knowledge exchange portals assist in knowledge management for evidence-informed decision making in public health?. *BMC Public Health*, *14*(1). http://dx.doi.org/10.1186/1471-2458-14-443

Rajić, A., Young, I., & McEwen, S. (2013). Improving the Utilization of Research Knowledge in Agri-food Public Health: A Mixed-Method Review of Knowledge Translation and Transfer. *Foodborne Pathogens And Disease*, *10*(5), 397-412. http://dx.doi.org/10.1089/fpd.2012.1349

Rebolledo, J., Garvey, P., Ryan, A., O'donnell, J., Cormican, M., & Jackson, S. et al. (2013). International outbreak investigation of Salmonella Heidelberg associated with in-flight catering. *Epidemiology And Infection*, *142*(04), 833-842. http://dx.doi.org/10.1017/s0950268813001714

Roberts, G. (2015). Communities of practice: Exploring enablers and barriers with school health clinicians. *Canadian Journal Of Occupational Therapy*, *82*(5), 294-306. http://dx.doi.org/10.1177/0008417415576776

Smith, J, Flowers, P, & Larkin, M. (2009). *Interpretative phenomenological analysis: Theory, method and research*. Thousand Oaks, California: Sage.

Tsang, S., Royse, C., & Terkawi, A. (2017). Guidelines for developing, translating, and validating a questionnaire in perioperative and pain medicine. *Saudi Journal Of Anaesthesia*, *11*(5), 80. http://dx.doi.org/10.4103/sja.sja_203_17

Verburg, R., & Andriessen, J. (2006). The assessment of communities of practice. *Knowledge And Process Management*, *13*(1), 13-25. http://dx.doi.org/10.1002/kpm.241

Wenger, E., McDermott, R. and Snyder, W. (2002). *Cultivating communities of practice*. Boston, Mass.: Harvard Business School Press.

Wenger, E., Trayner, B., and de Laat, M. (2011). Promoting and assessing value creation in communities and networks: a conceptual framework. Rapport 18, Ruud de Moor Centrum, Open University of the Netherlands.

World Health Organization. (2014). *Advancing food safety initiative: strategic plan for food safety including foodborne zoonoses 2013-2022*. Retrieved from: http://www.who.int/foodsafety/publications/strategic-plan/en/.

World Health Organization. (2015). *Global burden of foodborne disease report*. Retrieved from: http://apps.who.int/iris/bitstream/10665/200046/1/WHO_FOS_15.02_eng.pdf?ua=1.

For peer review only

World Health Organization. (2016). *INFOSAN Activity Report 2014/2015*. Retrieved from: http://apps.who.int/iris/bitstream/10665/246204/1/9789241510592-eng.pdf.

World Health Organization. (2018). *Process of translation and adaptation of instruments*. Retrieved from: http://www.who.int/substance_abuse/research_tools/translation/en/.

For peer review only

Supplementary File 1 – Information sheet and permission form for the Director of the Department of Food Safety and Zoonoses at the World Health Organization

Seeking your authorization to conduct a mixed-methods exploration into the experience of members of the International Food Safety Authorities Network (INFOSAN)

Dear Dr Kazuaki Miyagishima – Director, Department of Food Safety and Zoonoses, WHO,

As you know, since 2015 I have been pursuing my PhD in Public Health at Lancaster University as documented in the WHO Performance Management and Development System. For the research phase of my studies I am proposing to examine the experiences of members of the International Food Safety Authorities Network (INFOSAN) and I am seeking your permission to do so. Attached you can find the full research proposal and below some summary details about the study. At the bottom of this form is a section for your signature should you choose to authorize the study to be carried out as described.

What is the study about?
The purpose of this study is to interrogate INFOSAN in order to describe and explore the experiences of members and better understand the role of the network in mitigating the burden of foodborne illness around the world. The study will examine access to and usage of the INFOSAN Community Website, explore barriers and facilitators to active participation in INFOSAN, determine perceptions about the utility of INFOSAN to mitigate foodborne illness, and scrutinize if and how participation in this network creates value for members.

Why have I been approached?
You have been approached because the study requires information from registered INFOSAN members and I am seeking your authorization to contact them for this purpose.

Do INFOSAN members have to take part?
No. It is completely up to each member to decide whether or not to take part. The study is designed in three phases: Phase 1 will examine access and usage patterns of the INFOSAN Community Website; Phase 2 will involve the completion of an online survey; and Phase 3 will involve individual interviews conducted online using WebEx. Taking part in one phase of the study does not require participation in the other phases.

What will members be asked to do if they take part?
Participation in Phase 1 of the study requires no further action from members. Data on access and usage of the INFOSAN Community Website has already been collected as part of the ongoing monitoring and evaluation activities of the INFOSAN Secretariat in accordance with the Terms and Conditions of Use of the INFOSAN Community Website. Any members not wishing to have their website access and usage data utilized for the purpose of this particular study will be requested to make this known by email to the researcher within a defined two-week period. Those who are recruited in Phase 1 will have a further two weeks to opt out of the study, after which time this will no longer be possible due to aggregation of data.

Participation in Phase 2 of the study requires the completion of an online survey of approximately 30-45 minutes which will explore questions about experience as an INFOSAN member. Once the survey has been submitted, it will not be possible to opt out because survey responses are anonymous.

Participation in Phase 3 of the study will involve a one-on-one individual interview conducted online using WebEx with the researcher. The interview will last approximately 45-60 minutes and will explore in more detail their experiences as an INFOSAN member. Those who are recruited for Phase 3 of the study will have two weeks to opt out after which time this will no longer be possible.

Continued on next page…

Continued from previous page…

Will collected data be identifiable?
No. The information provided by participants will be reported anonymously. Any direct quotes from participants will be reported anonymously. Quantitative data will be reported by region so that no country-specific details will be reported. The data collected for this study will be stored securely and only the researcher conducting this study will have access to this data:

- Audio recordings will be deleted once the thesis has been examined and accepted
- The files on the computer will be encrypted (that is no-one other than the researcher will be able to access them) and the computer itself password protected for the duration of the study
- At the end of the study, all related electronic information will be archived for 10 years in secure encrypted storage on the Lancaster University server. At the end of this period, they will be destroyed.
- The typed version of your interview will be made anonymous by removing any identifying information including participant names. Anonymised direct quotations from your interview may be used in the reports or publications from the study, and neither participant name nor your country will be attached to them.
- All personal data will be confidential and will be kept separately from interview responses.

There are some limits to confidentiality: if what is said in the interview makes me think that a participant, or someone else, is at significant risk of harm, I will have to break confidentiality and speak to a member of staff about this. If possible, I will tell you if I have to do this.

What will happen to the results?
The results will be summarised and reported in a PhD thesis and may be submitted for publication in relevant academic conferences and journals, pending necessary clearance from WHO. Only those data collected with the expressed consent of participants is utilized and reported on for the purposes of this study. INFOSAN Members will also be invited to participate in webinars after each phase to learn about the results and next steps.

Are there any risks?
There are no risks anticipated with participating in this study. However, if a participant experiences any distress following participation they will be encouraged to inform the researcher and contact the resources provided in advance. Neither participating nor abstaining will impact their future treatment as an INFOSAN member or the technical support provided to them or their country by the WHO. This will be made clear to all INFOSAN members.

Are there any benefits to taking part?
Although members may find participating interesting, there are no direct benefits in taking part.

Who has reviewed the project?
This study will be reviewed and approved by the Ethics Review Committee at the World Health Organization and the Faculty of Health and Medicine Research Ethics Committee at Lancaster University.

Kind regards,

Carmen Savelli

Subject to clearance by the Ethics Review Committee at the World Health Organization and the Faculty of Health and Medicine Research Ethics Committee at Lancaster University, I, Kazuaki Miyagishima, Director, Department Food Safety and Zoonoses at WHO, authorize the conduct of the proposed research as part of his PhD studies at Lancaster University as described above and in the accompanying proposal, titled, 'A mixed-methods exploration into the experience of members of the International Food Safety Authorities Network (INFOSAN)' as prepared by Carmen Savelli.

Signature

19/VI/2018
Date

Supplementary File 2 – Information Email #1: Introductory information about the study (including the overall Participant Information Sheet) and invitation to attend information webinar #1

To be sent by the INFOSAN Secretariat (infosan@who.int) on behalf of the researcher (cc: c.savelli@lancaster.ac.uk)

Health & Medicine | Lancaster University

Information Email #1

A mixed-methods exploration into the experience of members of the International Food Safety Authorities Network (INFOSAN): Introductory Information

---

Dear INFOSAN Member,

My name is Carmen Savelli and I am conducting this study as a student in the PhD Public Health programme at Lancaster University, Lancaster, United Kingdom. You may also know me as one of the Technical Officers working as the INFOSAN Secretariat at the World Health Organization (WHO) in Geneva, Switzerland.

What is the study about?
The purpose of this study is to interrogate INFOSAN in order to describe and explore the experiences of members and better understand the role of the network in mitigating the burden of foodborne illness around the world. The study will examine access to and usage of the INFOSAN Community Website, explore barriers and facilitators to active participation in INFOSAN, determine perceptions about the utility of INFOSAN to mitigate foodborne illness, and scrutinize if and how participation in this network creates value for members.

Why have I been approached?
You have been approached because the study requires information from registered INFOSAN members.

Do I have to take part?
No. It is completely up to you to decide whether or not you take part. The study is designed in three phases: Phase 1 will examine access and usage patterns of the INFOSAN Community Website; Phase 2 will involve the completion of an online survey; and Phase 3 will involve individual interviews conducted online using WebEx. Taking part in one phase of the study does not require your participation in the other phases.

What will I be asked to do if I take part?
Participation in Phase 1 of the study requires no further action on your part. Data on access and usage of the INFOSAN Community Website has already been collected as part of the ongoing monitoring and evaluation activities of the INFOSAN Secretariat in accordance with the Terms and Conditions of Use of the INFOSAN Community Website. **Any members not wishing to have their website access and usage data utilized for the purpose of this particular study should make this known by email to the researcher within the next two weeks (c.savelli@lancaster.ac.uk).** Those who are recruited in Phase 1 will have a further two weeks to opt out of the study, after which time this will no longer be possible due to aggregation of data.

Participation in Phase 2 of the study requires the completion of an online survey of approximately 30-45 minutes which will explore questions about your experience as an INFOSAN member. Once the survey has been submitted, it will not be possible to opt out because survey responses are anonymous.

Participation in Phase 3 of the study will involve a one-on-one individual interview conducted online using WebEx with the researcher. The interview will last approximately 45-60 minutes and will explore in more detail your experiences as an INFOSAN Member. Those who are recruited for Phase 3 of the study will have two weeks to opt out after which time this will no longer be possible.

Continued on next page…

Continued from previous page…

Will my data be Identifiable?
No. The information you provide will be reported anonymously. Any direct quotes from participants in Phase 3 will be reported anonymously. Quantitative data will be reported by region so that no country-specific details will be reported. The data collected for this study will be stored securely and only the researchers conducting this study will have access to this data:

- Audio recordings will be deleted once the thesis has been examined and accepted
- The files on the computer will be encrypted (that is no-one other than the researcher will be able to access them) and the computer itself password protected for the duration of the study
- At the end of the study, all related electronic information will be archived for 10 years in secure encrypted storage on the Lancaster University server. At the end of this period, they will be destroyed.
- The typed version of your interview will be made anonymous by removing any identifying information including your name. Anonymised direct quotations from your interview may be used in the reports or publications from the study, and neither your name nor your country will be attached to them.
- All your personal data will be confidential and will be kept separately from your interview responses (if you participate in Phase 3). However, there are some limits to confidentiality in Phase 3: if what is said in the interview makes me think that you, or someone else, is at significant risk of harm, I will have to break confidentiality and speak to a member of staff about this. If possible, I will tell you if I have to do this. In addition, whilst every effort will be made, it is not possible for the researcher to ensure confidentiality of your participation in Phase 3 if you choose to connect to the researcher by WebEx on your work premises during the working day. If you volunteer to participate in Phase 3, you are encouraged to connect by WebEx in a private room to conduct your interview, where no one else can see your computer screen or hear your conversation.

For further information about how Lancaster University processes personal data for research purposes and your data rights please visit our webpage: www.lancaster.ac.uk/research/data-protection

What will happen to the results?
The results will be summarised and reported in a PhD thesis and may be submitted for publication in relevant academic or professional conferences and journals or other media including books or websites, pending necessary clearance from WHO. Only those data collected with the expressed consent of participants is utilized and reported on for the purposes of this study. INFOSAN Members will also be invited to participate in webinars after each phase to learn about the results and next steps.

Are there any risks?
There are no risks anticipated with participating in this study. However, if you experience any distress following participation you are encouraged to inform the researcher and contact the resources provided at the end of this sheet. Neither participating nor abstaining will impact your future treatment as an INFOSAN member or the technical support provided to you or your country by the WHO.

Are there any benefits to taking part?
Although you may find participating interesting, there are no direct benefits in taking part. However, some participants may find it a positive experience to reflect on their role as an INFOSAN member as well as their contributions to a safer food supply, and to participate in research which they may feel is of relevance to them, their institution, their country or the global community.

Who has reviewed the project?
This study has been reviewed and approved by the Ethics Review Committee at the World Health Organization and the Faculty of Health and Medicine Research Ethics Committee at Lancaster University.

Where can I obtain further information about the study if I need it?
If you have any questions about the study, please contact the researcher:

Carmen Savelli
c.savelli@lancaster.ac.uk
+41 22 791 3234

Or his primary supervisor in the Department of Health Research at Lancaster University:

Dr Céu Mateus
c.mateus@lancaster.ac.uk
+44 1524 593182

Continued on next page…

Continued from previous page…

Or attend the upcoming webinar:

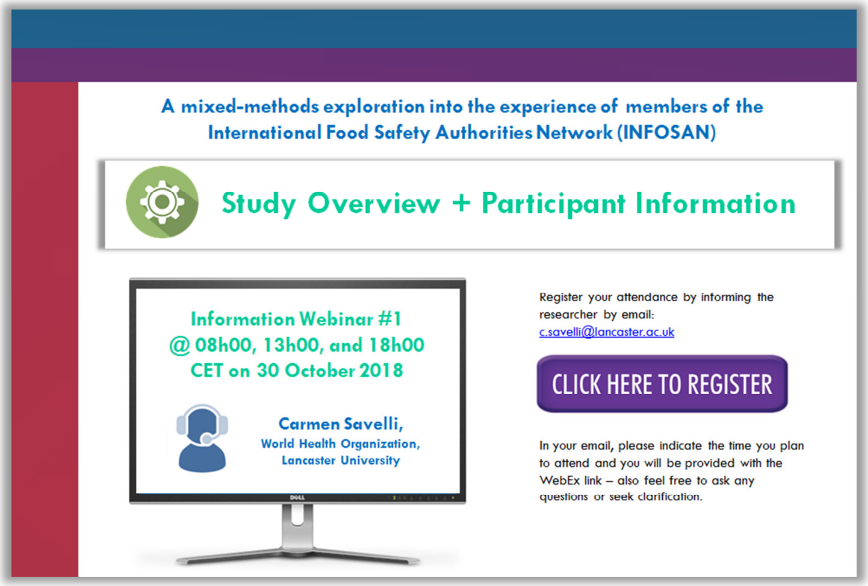

Complaints
If you wish to make a complaint or raise concerns about any aspect of this study and do not want to speak to the researcher or his supervisor, you can contact:

Professor Catherine Walshe
Head of the Division of Health Research
+44 (0)1524 510124
c.walshe@lancaster.ac.uk
Lancaster University
Lancaster
LA1 4YW

If you wish to speak to someone outside of the Public Health Doctorate Programme, you may also contact:

Professor Roger Pickup Tel: +44 (0)1524 593746
Associate Dean for Research Email: r.pickup@lancaster.ac.uk
Faculty of Health and Medicine
(Division of Biomedical and Life Sciences)
Lancaster University
Lancaster
LA1 4YG

Thank you for taking the time to read this information.

Resources in the event of distress
Should you feel distressed either as a result of taking part, or in the future, the following resource may be of assistance:
https://online.supportgroups.com/

Kind regards,

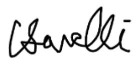

Carmen Savelli

Supplementary File 3 – Terms and Conditions of Use of the INFOSAN Community Website

The following disclaimer text is displayed on the online registration form of the ICW (https://extranet.who.int/infosan/registration) and must be accepted by all INFOSAN members to in order to complete registration.

Please take note of the following terms of use and disclaimers. By accessing the International Food Safety Authorities Network (INFOSAN) Community Website, the user agrees to accept these terms of use and disclaimers. The Food and Agriculture Organization of the United Nations (FAO) and the World Health Organization (WHO) reserve the right to update these terms of use and disclaimers without notice.

The INFOSAN Community Website is intended for the use of members of INFOSAN for the purpose of managing information about food safety risks in an interactive and collaborative manner. The content within the INFOSAN Community Website is intended for the internal use of registered INFOSAN members only and may not be disclosed, shared, distributed or transmitted to persons outside the Network, or duplicated, republished, reused for presentation or used in any other way, including for advertising or commercial purposes, without the prior written consent from FAO and WHO.

Although all reasonable precautions have been taken to verify the content contained on the INFOSAN Community Website, FAO and WHO make no warranties or representations regarding the content, presentation, appearance, completeness or accuracy and shall not be held liable for any damages whatsoever as a result of its use or application. The responsibility for the interpretation and use of the content lies with the reader and/or user. FAO and WHO reserve the right to make updates and changes without notice and accept no liability for any errors or omissions in this regard.

FAO and WHO accept no responsibility whatsoever for any inaccurate advice or information that is provided by sources reached via links or references to the content of the INFOSAN Community Website.

The INFOSAN Community Website may contain links to other Internet pages. With regard to these third-party websites, FAO and WHO have no control whatsoever over the content of the linked pages. FAO and WHO therefore shall not be held responsible whatsoever for the content of a page to be reached via such a link and shall not be held liable for any damages arising from their use. FAO and WHO reserve the right to make changes or additions, without prior notification, to the links provided.

The views expressed on the INFOSAN Community Website are those of the authors and do not necessarily reflect those of FAO and WHO.

The designations employed and the presentation of content on the INFOSAN Community Website, including maps and other illustrative materials, do not imply the expression of any opinion whatsoever on the part of FAO or WHO concerning the legal status of any country, territory, city or area, or of its authorities, or concerning the delineation of frontiers and borders.

The mention of specific companies or of certain manufacturers' products does not imply that they are endorsed or recommended by FAO or WHO in preference to others of a similar nature that are not mentioned.

Utilization of website analytics tools or other methods will be applied periodically to summarize members' access to and usage of the INFOSAN Community Website for monitoring, evaluation and research purposes.

[ ] Accept Terms & Conditions of Use

For peer review only

Supplementary File 4 – INFOSAN Community Website Online Registration Form

Please enter your primary e-mail address to be used for communication with the INFOSAN Secretariat. You will also use this e-mail address to log in to the INFOSAN Community Website.

Account information

E-mail: *

** Indicates a mandatory field*

Type of member: *

- FAO Regional Officer
- WHO Collaborating Centre
- FAO Staff
- IHR Regional Focal Point
- INFOSAN Advisory Group Member
- INFOSAN Emergency Contact Point
- INFOSAN Focal Point
- INFOSAN Secretariat
- RASFF Contact Point
- Regional Authority
- WHO Regional Advisor
- WHO Staff

Update My Profile

Title ? :

First Name: *

Last name: *

Sex:

--

Profile picture ? :

Browse... No file selected. Upload

Maximum file size: *100 MB*
Allowed extensions: *png gif jpg jpeg*

Languages spoken ? :

Language of correspondence ? :

--

Mailing Adress

Mailing Address: *

Mail Box:

Postal Code:

City: *

State / Province:

Country: *

Contact Details

Business Phone Number: *

Secondary Email:

Website:

Mobile Telephone:

Fax:

Professional Profile

Institution: *

Position:

Government Sector: *

- Agriculture
- Food Safety
- Human Health
- Trade and Commerce
- Animal Health
- Other

Primary Functions: *

- Risk assessment
- Risk communication/information
- Risk management
- Other

Areas of Scientific Expertise: *

- Biotechnology
- Food science and Technology
- Chemistry
- Microbiology
- Toxicology
- Foodborne Disease Surveillance
- Food Safety
- Epidemiology
- Animal Health
- Outbreak Investigation
- Public Health
- Emergency Management
- Other

Letter of Designation

Terms and Conditions of Use

Please take note of the following terms of use and disclaimers. By accessing the International Food Safety Authorities Network (INFOSAN) Community Website, the user agrees to accept these terms of use and disclaimers. The Food and Agriculture Organization of the United Nations (FAO) and the World Health Organization (WHO) reserve the right to update these terms of use and disclaimers without notice.

The INFOSAN Community Website is intended for the use of members of INFOSAN for the purpose of managing information about food safety risks in an interactive and collaborative manner. The content within the INFOSAN Community Website is intended for the internal use of registered INFOSAN members only and may not be disclosed, shared, distributed or transmitted to persons outside the Network, or duplicated, republished, reused for presentation or used in any other way, including for advertising or commercial purposes, without the prior written consent from FAO and WHO.

Accept Terms & Conditions of Use *

EEC7e

What code is in the image?: *

Enter the characters shown in the image.

Submit Registration Form to the INFOSAN Secretariat

Supplementary File 5 – Information Email #2: Results from Phase 1 and invitation to attend Webinar #2 (includes Phase 2 Participant Information)

To be sent by the INFOSAN Secretariat (infosan@who.int) on behalf of the researcher (cc: c.savelli@lancaster.ac.uk)

Health & Medicine | Lancaster University

Information Email #2

A mixed-methods exploration into the experience of members of the International Food Safety Authorities Network (INFOSAN): Phase 1 results and information about Phase 2

Dear INFOSAN Member,

My name is Carmen Savelli and in August 2018 I contacted you to let you know that I was conducting this study as a student in the PhD Public Health programme at Lancaster University, Lancaster, United Kingdom. You may also know me as one of the Technical Officers working as the INFOSAN Secretariat at the World Health Organization (WHO) in Geneva, Switzerland. I am writing again to inform you of the progress made so far, including results from Phase 1 and provide some additional information for participants in Phase 2.

What is the overall study about?
The purpose of this study is to interrogate INFOSAN in order to describe and explore the experiences of members and better understand the role of the network in mitigating the burden of foodborne illness around the world. The study will examine access to and usage of the INFOSAN Community Website, explore barriers and facilitators to active participation in INFOSAN, determine perceptions about the utility of INFOSAN to mitigate foodborne illness, and scrutinize if and how participation in this network creates value for members.

The study is designed in three phases: Phase 1 is complete and has examined access and usage patterns of the INFOSAN Community Website; Phase 2 will involve the completion of an online survey; and Phase 3 will involve individual interviews conducted online using WebEx. Taking part in one phase of the study does not require your participation in the other phases.

What are the results from Phase 1?

To be inserted

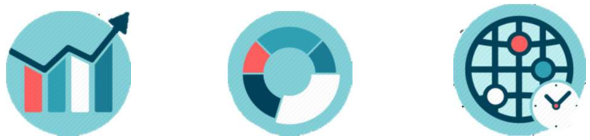

Why have I been approached about Phase 2 of this study?
You have been approached because the study requires information from registered INFOSAN members.

Do I have to take part in Phase 2?
No. It is completely up to you to decide whether or not you take part. Once the survey has been submitted, it will not be possible to opt out because survey responses are anonymous.

Continued on next page…

Continued from previous page…

What will I be asked to do if I take part?
Participation in Phase 2 of the study requires the completion of an online survey of approximately 30-45 minutes which will explore questions about your experience as an INFOSAN member. All INFOSAN Members will receive a separate invitation by email(Information Email #3) to provide consent and take the survey.

Will my data be Identifiable?
No. The information you provide will be reported anonymously. Quantitative data will be reported by region so that no country-specific details will be reported. The data collected for this study will be stored securely and only the researchers conducting this study will have access to this data:

- The files on the computer will be encrypted (that is no-one other than the researcher will be able to access them) and the computer itself password protected for the duration of the study
- At the end of the study, all related electronic information will be archived for 10 years in secure encrypted storage on the Lancaster University server. At the end of this period, they will be destroyed.

For further information about how Lancaster University processes personal data for research purposes and your data rights please visit our webpage: www.lancaster.ac.uk/research/data-protection

What will happen to the results?
The results will be summarised and reported in a PhD thesis and may be submitted for publication in relevant academic or professional conferences and journals or other media including books or websites, pending necessary clearance from WHO. Only those data collected with the expressed consent of participants is utilized and reported on for the purposes of this study. INFOSAN Members will also be invited to participate in a webinar after Phase 2 to learn about the results and next steps.

Are there any risks?
There are no risks anticipated with participating in this study. However, if you experience any distress following participation you are encouraged to inform the researcher and contact the resources provided at the end of this sheet. Neither participating nor abstaining will impact your future treatment as an INFOSAN member or the technical support provided to you or your country by the WHO.

Are there any benefits to taking part?
Although you may find participating interesting, there are no direct benefits in taking part. However, some participants may find it a positive experience to reflect on their role as an INFOSAN member as well as their contributions to a safer food supply, and to participate in research which they may feel is of relevance to them, their institution, their country or the global community.

Who has reviewed the project?
This study has been reviewed and approved by the Ethics Review Committee at the World Health Organization and the Faculty of Health and Medicine Research Ethics Committee at Lancaster University.

Where can I obtain further information about the study if I need it?
If you have any questions about the study, please contact the researcher:

Carmen Savelli
c.savelli@lancaster.ac.uk
+41 22 791 3234

Or his primary supervisor in the Department of Health Research at Lancaster University:

Dr Céu Mateus
c.mateus@lancaster.ac.uk
+44 1524 593182

Or attend the upcoming webinar:

Continued on next page…

Continued from previous page…

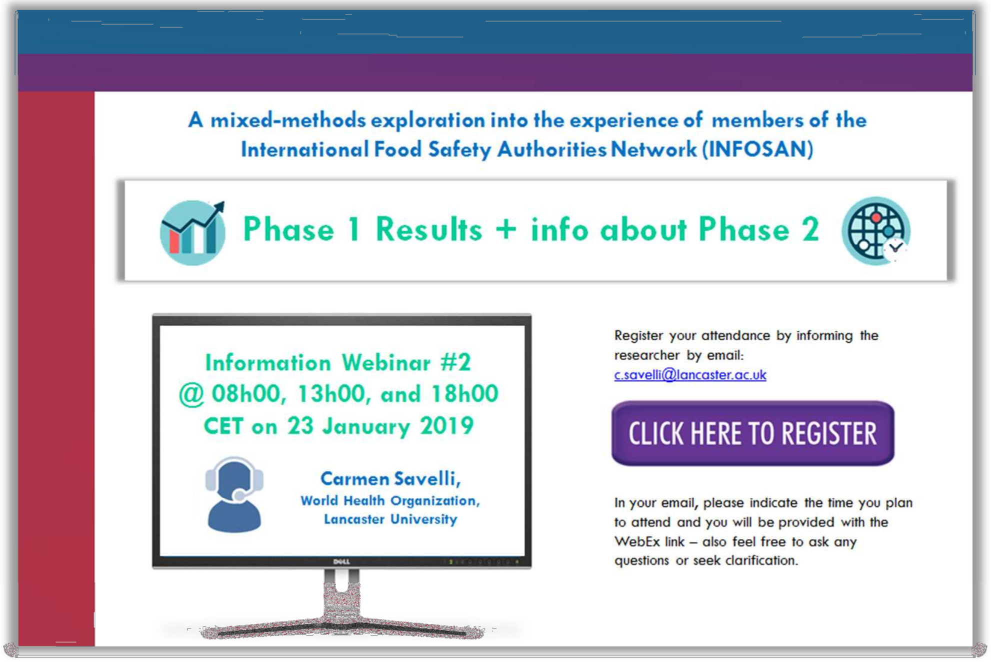

Complaints
If you wish to make a complaint or raise concerns about any aspect of this study and do not want to speak to the researcher or his supervisor, you can contact:

Professor Catherine Walshe
Head of the Division of Health Research
+44 (0)1524 510124
c.walshe@lancaster.ac.uk
Lancaster University
Lancaster
LA1 4YW

If you wish to speak to someone outside of the Public Health Doctorate Programme, you may also contact:

Professor Roger Pickup Tel: +44 (0)1524 593746
Associate Dean for Research Email: r.pickup@lancaster.ac.uk
Faculty of Health and Medicine
(Division of Biomedical and Life Sciences)
Lancaster University
Lancaster
LA1 4YG

Thank you for taking the time to read this information.

Resources in the event of distress
Should you feel distressed either as a result of taking part, or in the future, the following resource may be of assistance:
https://online.supportgroups.com/

Kind regards,

CSavelli

Carmen Savelli

Supplementary File 6 – Information Email #3: Invitation to participate in Phase 2 including consent information and link to online survey

To be sent by the INFOSAN Secretariat (infosan@who.int) on behalf of the researcher (cc: c.savelli@lancaster.ac.uk)

Health & Medicine | Lancaster University

Information Email #3

A mixed-methods exploration into the experience of members of the International Food Safety Authorities Network (INFOSAN): Invitation to participate in Phase 2

Dear INFOSAN member,

You are invited to participate in Phase 2 of our study to explore the experiences of members of the International Food Safety Authorities Network (INFOSAN).

Phase 2 of the study involves completing an online survey that should take approximately 30-45 minutes of your time. Complete background details are available in the Participant Information Sheet, shared with you previously and also attached for ease of reference.

By proceeding to the survey you confirm that:

- ✓ You have read the information sheet and understand what is expected of you within this study;
- ✓ You confirm that you understand that any responses/information you give will remain anonymous;
- ✓ Your participation is voluntary;
- ✓ You consent for the information you provide to be discussed with my supervisor at Lancaster University;
- ✓ You consent to Lancaster University keeping the anonymised data for a period of 10 years after the study has finished;
- ✓ By clicking on the button below, you consent to taking part in the current study.

If you have any questions about this survey or other aspects of the study, please do not hesitate to contact me at any time: c.savelli@lancaster.ac.uk

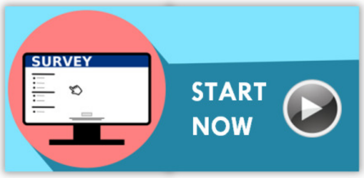

Kind regards,

Carmen Savelli

Supplementary File 7 – Draft questionnaire for Phase 2

Most of the following questions have been taken directly from the Community Assessment Tool (Verburg and Andriessen, 2006). Those questions in written in blue text are supplementary questions, added to the questionnaire in order to address INFOSAN-specific items. In addition, the likert-type scale response anchors have been added as per Vagias and Wade (2006).

PERSONAL DATA

Age ___________

Gender ___________

Country ___________

**A) INTRODUCTION**
Are you a member of more than one food-safety related community of practice?<br><br>If yes, which one(s): ____

**How long (approximately) have you been a member of INFOSAN?**					
	Weeks	Months	Years	Don't know	Prefer not to answer
Specify number in corresponding box	#	#	#		

	Focal Point	Emergency Contact Point	Don't know	Prefer not to answer
**What type of member are you?**				

**B) GOALS**
**How important are the following goals for you personally as a member of this community? That means, for you personally, what are the most important reasons for participating in INFOSAN?**

For peer review only

	Not Important At All	Of Little Importance	Of Average Importance	Very Important	Absolutely Essential	Don't know	Prefer not to answer
– Hearing about new knowledge and experiences from other community members							
– Developing together new ideas for the community							
– Having nice meetings, fun and non-work related activities							
– Developing standards, methods and best practices							
– Making useful contacts/ networking							
– Improving the level of expertise of other members							
– Staying up to date in the topic of the community (i.e. food safety issues)							
– Saving time in finding all kinds of information							
– To advance in my career							
– Helping newcomers in the community							

C) ACTIVITIES

How often do the following activities happen in your community?

	Never	Almost Never	Occasionally	Frequently	Very Frequently	Don't know	Prefer not to answer
– Meetings with discussions							
– Talks about experiences							
– Presentations by members							
– Presentations by non-members							
– Workshops							
– Team-building activities							
– Members writing reports or other publications together							
– Brainstorming							
– Writing project proposals							
– Exchanging e-mails to find solutions to problems							

To what extent would you prefer a change in these activities?

	Decrease frequency greatly	Decrease frequency slightly	No change	Increase Frequency slightly	Increase frequency greatly	Don't know	Prefer not to answer

For peer review only

– Meetings with discussions							
– Talks about experiences							
– Presentations by members							
– Presentations by non-members							
– Workshops							
– Team building activities (such as informal outings)							
– Brainstorming							
– Writing project proposals							
– Members writing publications together							
– Exchanging e-mails to find solutions to problems							

	Very poor	Poor	Acceptable	Good	Very Good	Don't know	Prefer not to answer
**How would you rate the way people interact in this community?**							

Does your community have the following types of meetings?

	Never	Almost Never	Occasionally	Frequently	Very Frequently	Don't know	Prefer not to answer
– Face to face meetings							
– Virtual meetings (e.g. video conferences, telephone conferences, via the internet)							

D) PARTICIPATION

	Not at all	Almost never	Occasionally	Very	Extremely	Don't know	Prefer not to answer
**How actively do you participate in the community?**							

**How often do you have the following types of contact with other members of your community outside of community meetings?**							
	Never	Almost Never	Occasionally	Frequently	Very Frequently	Don't know	Prefer not to answer
– Email exchanges							
– Phone calls							
– Face to face meetings							
– Virtual meetings (e.g. video conferences, telephone conferences, via the internet)							
– Instant chats on the INFOSAN Community Website							
– Discussion forum on the INFOSAN Community Website							

On average, how many hours do you spend on the community per month? _________

How many members do you know by face? _____________

With how many members have you had personal contact? _______________

INFOSAN has an important mandate, to communicate between national government authorities around the world to keep the global food supply safe. However, some members are more active than others. The following section pertains to potential barriers to participation in INFOSAN.

**To what extent do you agree that the following factors limit your participation in INFOSAN Activities?**							
	Strongly Disagree	Disagree	Undecided	Agree	Strongly Agree	Don't know	Prefer not to answer
My role as an INFOSAN member is unclear to me							
My country has limited capacity and/or infrastructure dedicated to addressing food safety events							
My country has insufficient funds dedicated to monitoring and/or responding to food safety events.							
My country lacks human resources and/or expertise dedicated to addressing food safety events.							
My country's national food control system is either underdeveloped or non-existent							

For peer review only

My country's national-level Food Safety Emergency Response Plan is either underdeveloped or non-existent							
There are difficulties in identifying contaminated food and/or ingredients within my country							
There are challenges in conducting food safety risk assessments within my country							
Personnel in my country are not trained to respond to food safety events							
There is a need for a simpler and more standardized way to share information between national authorities within my country							
There is a lack of coordination and/or communication between national authorities within my country							
Legal implications at the national level hinder prompt information-sharing with the INFOSAN Secretariat and INFOSAN member countries							
12. There are political constraints at the national level (e.g. the government does not prioritize food safety, the Emergency Contact Point must reflect national views when communicating with the INFOSAN Secretariat, etc.)							
There are concerns in my country that reporting a food safety event with potential international implications might negatively impact the country's economy or competitive interests (e.g. trade, tourism, etc.)							
There are language barriers.							
There is a lack of trust between INFOSAN members							
There are fears that the confidential nature of information shared may not be respected by parties involved (e.g. the information may not be safeguarded, may be disseminated inappropriately, etc.)							

E) COORDINATION

How active is the co-ordinator/ facilitator of your community (i.e. the INFOSAN Secretariat) in the following activities?

	Never	Almost Never	Occasionally	Frequently	Very Frequently	Don't know	Prefer not to answer
– Organising meetings							
– Stimulating members to participate in the community							
– Sharing his/ her own expertise with the community members							
– Co-ordinating activities on the INFOSAN Community Website							
– Making external contacts							
– Connecting the community members with each other							

– Promoting the community publicly							
– Alerting members to interesting external activities (e.g. conferences)							

To what extent do you agree that the following statements about the INFOSAN Secretariat?

	Strongly Disagree	Disagree	Neither Agree nor disagree	Agree	Strongly Agree	Don't know	Prefer not to answer
I fully understand the role of the INFOSAN Secretariat.							
Information communicated by the INFOSAN Secretariat is always sufficiently prompt.							
Information communicated by the INFOSAN Secretariat is always accurate.							

	Very dissatisfied	Dissatisfied	Neutral	Satisfied	Very Satisfied	Don't know	Prefer not to answer
**How satisfied are you with the co-ordination of INFOSAN?**							

F) INFORMATION AND COMMUNICATION TECHNOLOGY SUPPORT

How useful do you think the following means of communication are (or might be) particularly for the work of INFOSAN?

	Not useful at all	Not very useful	No opinion	Very Useful	Essential	Don't know	Prefer not to answer
– Scheduled face to face meetings							
– Informal face to face encounters							
– E-mail							
– Telephone conferences							
– Fax							
– Video connection							
– Chat/ shared white board/ application sharing system							
– Special discussion list / newsgroup for the community							

For peer review only

– Written memos or reports							
– Group calendar/ shared project planning tool							
– Document sharing tool							
– Groupware to work jointly on documents (co-editing)							
– A database/ knowledge system in which you are expected to fill in your project experiences and relevant knowledge							

The INFOSAN Community Website was launched in 2012 and all members of INFOSAN are registered on there. This online portal is the gateway through which all members can communicate with each other and with the INFOSAN Secretariat.

**Regarding the INFOSAN Community Website:**							
	Never	Every few months	Monthly	Weekly	Daily	Don't know	Prefer not to answer
How often do you access the INFOSAN Community Website?							

**What do you normally use the INFOSAN Community Website for? (check all that apply)**	
	To read about Alerts issued by the INFOSAN Secretariat
	To read INFOSAN Documents
	To read publications and newsletters from FAO/WHO
	To participate in the Discussion Forum
	To find other INFOSAN members' contact details
	To discuss with other INFOSAN members using the "Chat" function on the INFOSAN Community Website
	To participate in or manage Groups
	Other; Please specify
	Don't know
	Prefer not to answer

**To what extent do you agree that the following statements about the INFOSAN Community Website?**							
	Strongly Disagree	Disagree	Neither Agree nor disagree	Agree	Strongly Agree	Don't know	Prefer not to answer
The INFOSAN Community Website an important and supportive tool for the network							
The volume of information posted on the INFOSAN							

For peer review only

Community Website by the **Secretariat** is sufficient							
The volume of information posted on the INFOSAN Community Website by the **members** is sufficient.							
A mobile friendly version of the INFOSAN Community Website would improve participation							
The INFOSAN Community Website provides a single integrated point of access to a variety of relevant food safety emergency information for INFOSAN members around the world.							
The INFOSAN Community Website creates and maintains knowledge directories about portal generated content including details about food safety events and emergencies.							
The INFOSAN Community Website facilitates information sharing and provides collaborative features that help to foster the Community of Practice among INFOSAN members							
The INFOSAN Community Website facilitates information sharing and provides collaborative features that help to foster the Community of Practice among INFOSAN members							

G) ORGANIZATIONAL SUPPORT							
	Never	Almost Never	Occasionally	Frequently	Very Frequently	Don't know	Prefer not to answer
Does your organisation allocate time for you to participate in INFOSAN?							
	Strongly discouraged	Discouraged	Neither discouraged or encouraged	Encouraged	Strongly Encouraged	Don't know	Prefer not to answer
All in all how encouraged do you feel by your organisation to participate actively in INFOSAN?							

	Not at all	To a small extent	To a moderate extent	To a great extent	To a very great extent	Don't know	Prefer not to answer

Would you like to have more time available for activities concerning INFOSAN?							

H) VALUE OF INFOSAN AS A COMMUNITY OF PRACTICE

How important are the following ways for you to find new information (e.g. for solving problems, for doing projects, for keeping up to date in your field)?

	Not important at all	Not very important	Important	Very important	Extremely important	Don't know	Prefer not to answer
– Through INFOSAN							
– Through individual colleagues							
– Via the internet							
– Asking an expert							
– Reading publications or reports							
– Through database consulting							
– Other	explain						

To what extent would you personally like INFOSAN to continue?

	Not at all	To a small extent	To a moderate extent	To a great extent	To a very great extent	Don't know	Prefer not to answer

Please indicate the extent to which you agree with the following statements.

	Strongly Disagree	Disagree	Neither Agree nor disagree	Agree	Strongly Agree	Don't know	Prefer not to answer

For peer review only

– Some knowledge is so valuable to me that I would not easily share it with others							
– The most important thing that happens in our community is that members find solutions to problems in their work							
– Knowledge is now so developed in the community that it can be turned into innovative ideas							
– Thanks to the combination of the different skills and views in our community many new ideas have been developed							
– People in the community are reluctant to talk about things that have gone wrong in their projects/ work							
– Newcomers have much to learn before they are at the same level as the existing members							
– It is sometimes difficult to learn from each other in the community because we have very different backgrounds							
– Knowledge is now so developed in the community that it can be written up for e.g. manuals, training courses, best practices							
– Most members in this community have developed a strong common understanding and identity over time							

**How often does it happen that:**							
	Never	Almost Never	Occasionally	Frequently	Very Frequently	Don't know	Prefer not to answer
You have some knowledge or experience that you prefer NOT to share with members of the community							
You hear experiences and other things that you already know							

For peer review only

INFOSAN has been in operation since 2004, and this study is the first of its kind to try and determine if the network has value to members in a robust and rigorous way.

**Please indicate the extent to which you agree with the following statements.**							
	Strongly Disagree	Disagree	Neither Agree no disagree	Agree	Strongly Agree	Don't know	Prefer not to answer
Participating in INFOSAN has been a valuable							

experience for me							
Participating in INFOSAN has connected me with other food safety professionals around the world.							
Participating in INFOSAN has prevented foodborne illnesses in other countries							
Participating in INFOSAN makes me feel a part of a global community of practice of food safety professions.							

Participating in INFOSAN has provided me with information that was used in my country to **implement risk management measures**.

[ ] Yes [ ] No [ ] Don't know

If Yes, during how many different food safety events? ☐1 ☐2-5 ☐ 6-10 ☐11-20 ☐ >20

If Yes, can you name an example? ____

Participating in INFOSAN has **prevented foodborne illnesses** in my country.

[ ] Yes [ ] No [ ] Don't know

If Yes, during how many different food safety events? ☐1 ☐2-5 ☐ 6-10 ☐11-20 ☐ >20

If Yes, can you name an example? ____

I) RESULTS							
**To what extent do you think the members of INFOSAN . . .**							
	Not at all	Almost never	Somewhat	Mostly	Completely	Don't know	Prefer not to answer
– Trust each other							
– Feel a sense of loyalty to INFOSAN							
– Have a good common understanding							
– Feel a sense of belonging to INFOSAN							

**To what extent do you think INFOSAN has. . .**

For peer review only

	Not at all	Almost never	Seldom	Frequently	Constantly	Don't know	Prefer not to answer
– Contributed to cost savings for your organisation							
– Contributed new ideas to your organisation<br>– Made a real contribution to the effectiveness of your organisation							
– Contributed to developing new methods or approaches for your organisation							
– Contributed to the documentation of information, such as knowledge systems, manuals, training instructions or best practices							
– A good reputation in your organisation							

**To what extent do you think the members are . . .**							
	Never	Rarely	Sometimes	Often	Always	Don't know	Prefer not to answer
– Reluctant to share their knowledge with other members							
– Generally very enthusiastic and motivated to participate							

**Through being a member of INFOSAN, to what extent…**							
	Not at all	Slightly	Somewhat	Moderately	Extremely	Don't know	Prefer not to answer
– Have you learned a lot about your subject area?							
– Have you made useful new contacts?							
– Have you been able to solve problems in your work?							
– Have your career prospects improved?							
– Have you been better able to find all kinds of information?							
– Have you been able to work more efficiently?							
– Have you been better able to keep up to date in your field?							
– Has your reputation and visibility in the organisation improved?							

For peer review only

– Have you been able to transfer what you heard in the community to your project team(s)or department?							

All in all do you like being part of the community?

Yes [ ] **No [ ]** **Don't know [ ]** **Prefer not to answer [ ]**

**In your opinion, in what areas does the community need improvement? (check all that apply)**	
	– In the area of the tools that support community activities
	– The overall organization of the community
	– The way INFOSAN is supported by FAO/WHO
	– The co-ordination of INFOSAN
	– Other; Please specify:
	– Don't know
	– Prefer not to answer

Preface: INFOSAN aims to prevent the international spread of contaminated food and foodborne disease and strengthen food safety systems globally. The main objectives are to: 1) Promote the rapid exchange of information during food safety incidents/emergencies; 2) Share information on important food safety issues of global interest; 3) Promote partnership and collaboration between countries; and 4) Help countries strengthen their capacity to manage food safety emergencies.

Please indicate the extent to which you agree with the following statements.

	Strongly Disagree	Disagree	Neither Agree nor disagree	Agree	Strongly Agree	Don't know	Prefer not to answer
The objectives of INFOSAN are still valid							
The activities and outputs of INFOSAN are consistent with the overall goal and the attainment of its objectives							
The activities and outputs of the program are consistent with the intended impacts and effects							
The objectives of INFOSAN have been achieved							
INFOSAN activities are cost-efficient							
INFOSAN objectives are achieved in a timely manner							
INFOSAN activities are implemented in the most efficient way compared to alternatives							
Because of INFOSAN, illnesses have been prevented.							
Because of INFOSAN, lives have been saved.							
INFOSAN operates in a sustainable way.							

For peer review only

INFOSAN promotes the rapid exchange of information during food safety incidents/emergencies							
INFOSAN shares information on important food safety issues of global interest							
INFOSAN promotes partnership and collaboration between countries and networks							
INFOSAN helps countries strengthen their capacity to manage food safety emergencies							
INFOSAN has improved the safety of the global food supply.							
INFOSAN has reduced foodborne illness globally							
Information communicated by INFOSAN **members** is always sufficiently prompt							
Information communicated by INFOSAN **members** is always accurate							

For peer review only

Supplementary File 8 – INFOSAN Advisory Group Members

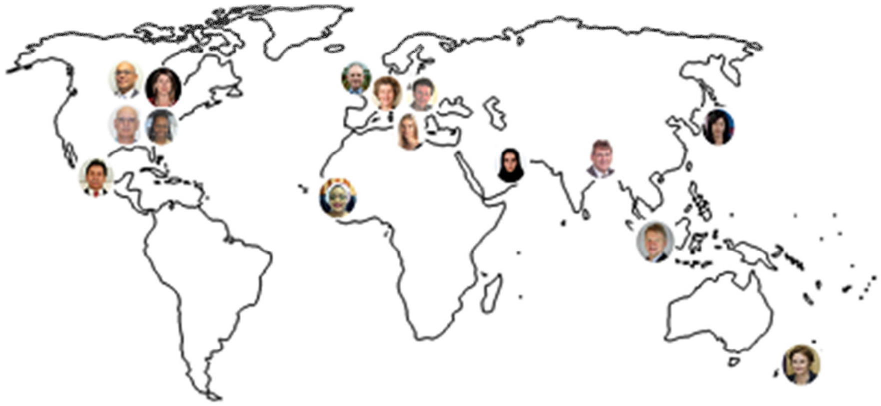

1. Dr Gillian Mylrea, World Organisation for Animal Health (OIE), France
2. Professor Alan Reilly, University College Dublin, Ireland
3. Ms Jenny Bishop, Ministry for Primary Industries, New Zealand
4. Dr Hugo Fragoso Sanchez, Agri-food Safety, Aquaculture and Fisheries of the National Health Service, Food Safety and Quality (SENASICA), Mexico
5. Dr Zainab Jallow, Food Safety and Quality Authority, The Gambia
6. Dr Peter Gerner-Smidt, Centers for Disease Control and Prevention (CDC), USA
7. Ms Isabelle Laberge, Canadian Food Inspection Agency, Canada
8. Mr Jan Baele, European Commission, Health and Food Safety, Belgium
9. Dr Caroline Merten, European Food Safety Authority, Italy
10. Dr Mariam Harib Al Yousuf Alsuwaidi, Abu Dhabi Food Control Authority, UAE
11. Ms Camille Brewer, Food and Drug Administration (FDA), USA
12. Mr Mark Samadhin, Public Health Agency of Canada, Canada
13. Dr Jørgen Schlundt, Nanyang Technological University, Singapore
14. Mr Terry Donohoe, Formerly Food Standards Agency (FSA), United Kingdom

For additional information about the INFOSAN Advisory Group, see details online: http://www.who.int/foodsafety/areas_work/infosan/INFOSAN-AG/en/

For peer review only

Supplementary File 9 - FAO/WHO Regional Food Safety Advisors/Officers

<u>WHO Regional Food Safety Advisors</u>

African Region
Ms Lusubilo Mwamakamba, Burkina Faso

Region of the Americas
Dr Enrique Perez Gutierrez, USA

Eastern Mediterranean Region
Mr Soren Madsen, Jordan

European Region
Dr Dina Pfeifer, Denmark

South East Asian Region
Dr Gyanendra Gongal, India

Western Pacific Region
Mr Peter Sousa Hoejskov, Philippines

<u>FAO Regional Food Safety Officers</u>

Near East and North Africa
Dr Fatima Hachem, Egypt

Asia
Dr Sridhar Dharmapuri, Thailand

Pacific
Ms Ann Hayman, Fiji

Latin America and the Caribbean
Dr Marisa Caipo, Chile

Europe and Central Asia
Ms Mary Kenny, Hungary

Africa
Dr Blaise Ouattara, Ghana

For peer review only

Supplementary File 10 – Expansion and Adaptation of Questionnaire

Verburg and Andriessen (2006)	1. Questionnaire in English from the Community Assessment Toolkit (CAT)
INFOSAN Advisory Group (Appendix 8)	2. Supplemental questions reviewed for content validity by a panel of experts consisting of Members of the INFOSAN Advisory Group
C. Savelli, Technical Officer, INFOSAN Secretariat, Department of Food Safety and Zoonoses (FOS)	3. Supplemental questions revised based on feedback prior to pilot testing
Regional Food Safety Officers and Advisors from FAO and WHO (Appendix 9)	4. Regional Food Safety Officers and Advisors from FAO and WHO pilot test the supplemental questions
C. Savelli	5. Revision prior to finalization in English
French translation: Roxanne Delagrave, WHO Intern, FOS<br>Spanish translation: Raúl Acevedo, WHO Intern, FOS	6. Forward translations of CAT questionnaire and supplemental questions into French and Spanish
**French Panel:**<br>▪ RC - PhD Candidate, formerly INFOSAN Secretariat at WHO<br>▪ CM – MD - WHO Implementation Research Scientist<br>▪ PBE - PhD, WHO, INFOSAN Secretariat<br>▪ RD - WHO Intern, FOS<br>**Spanish Panel**:<br>▪ EP – PhD, DVM, WHO Regional Food Safety Advisor<br>▪ SR – DVM, WHO Regional Food Safety Advisor<br>▪ JM – WHO, Technical Officer, FOS<br>▪ RG, WHO Intern, FOS	7. Review of entire questionnaire by expert panels
C. Savelli	8. Revision as required
WHO Translation services	9. Backward translations into English
C. Savelli	10. Revision as required
WHO Interns	11. Pilot test
C. Savelli	12. Revision as required to finalized version

Supplementary File 11 – Information Email #4: Results from Phase 2 and invitation to attend Webinar #3 (includes Phase 3 Participant Information)

To be sent by the INFOSAN Secretariat (infosan@who.int) on behalf of the researcher (cc: c.savelli@lancaster.ac.uk)

Health & Medicine | Lancaster University

Information Email #4

A mixed-methods exploration into the experience of members of the International Food Safety Authorities Network (INFOSAN): Phase 2 results and information about Phase 3

Dear INFOSAN Member,

My name is Carmen Savelli and in August 2018 I first contacted you to let you know that I was conducting this study as a student in the PhD Public Health programme at Lancaster University, Lancaster, United Kingdom. You may also know me as one of the Technical Officers working as the INFOSAN Secretariat at the World Health Organization (WHO) in Geneva, Switzerland. I am writing again to inform you of the progress made so far, including results from Phase 2 and provide some additional information for participants in Phase 3.

What is the overall study about?

The purpose of this study is to interrogate INFOSAN in order to describe and explore the experiences of members and better understand the role of the network in mitigating the burden of foodborne illness around the world. The study will examine access to and usage of the INFOSAN Community Website, explore barriers and facilitators to active participation in INFOSAN, determine perceptions about the utility of INFOSAN to mitigate foodborne illness, and scrutinize if and how participation in this network creates value for members.

The study is designed in three phases: Phase 1 is complete and has examined access and usage patterns of the INFOSAN Community Website; Phase 2 is complete and involved the completion of an online survey by INFOSAN members; and Phase 3 will involve individual interviews conducted online using WebEx. Taking part in one phase of the study does not require your participation in the other phases.

What are the results from Phase2?

To be inserted

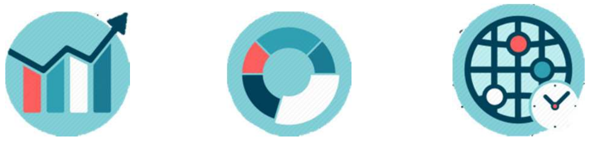

Why have I been approached about Phase 3 of this study?

You have been approached because the study requires information from registered INFOSAN members.

Do I have to take part in Phase 3?

No. It is completely up to you to decide whether or not you take part. Between 6 and 12 volunteers will be selected for Phase 3. If more than 2 people from a single WHO region indicate their interest in participating in Phase 3, the two people who have been members the longest will be selected and notified by email from the researcher, providing they are not from the same country.

Continued on next page…

Continued from previous page…

What will I be asked to do if I take part?

Participation in Phase 3 of the study will involve a one-on-one individual interview conducted online using WebEx with the researcher. The interview will last approximately 45-60 minutes and will explore in more detail your experiences as an INFOSAN Member.

To volunteer for Phase 3, please express your interest by contacting the researcher: c.savelli@lancaster.ac.uk

Will my data be Identifiable?

No. The information you provide will be reported anonymously. Any direct quotes from participants in Phase 3 will be reported anonymously. Quantitative data will be reported by region so that no country-specific details will be reported. The data collected for this study will be stored securely and only the researchers conducting this study will have access to this data:

- Audio recordings will be deleted once the thesis has been examined and accepted
- The files on the computer will be encrypted (that is no-one other than the researcher will be able to access them) and the computer itself password protected for the duration of the study
- At the end of the study, all related electronic information will be archived for 10 years in secure encrypted storage on the Lancaster University server. At the end of this period, they will be destroyed.
- The typed version of your interview will be made anonymous by removing any identifying information including your name. Anonymised direct quotations from your interview may be used in the reports or publications from the study, and neither your name nor your country will be attached to them.
- All your personal data will be confidential and will be kept separately from your interview responses. There are some limits to confidentiality: if what is said in the interview makes me think that you, or someone else, is at significant risk of harm, I will have to break confidentiality and speak to a member of staff about this. If possible, I will tell you if I have to do this. In addition, whilst every effort will be made, it is not possible for the researcher to ensure confidentiality of your participation in Phase 3 if you choose to connect to the researcher by WebEx on your work premises during the working day. If you volunteer to participate in Phase 3, you are encouraged to connect by WebEx in a private room to conduct your interview, where no one else can see your computer screen or hear your conversation.

For further information about how Lancaster University processes personal data for research purposes and your data rights please visit our webpage: www.lancaster.ac.uk/research/data-protection

What will happen to the results?

The results will be summarised and reported in a PhD thesis and may be submitted for publication in relevant academic or professional conferences and journals or other media including books or websites, pending necessary clearance from WHO. Only those data collected with the expressed consent of participants is utilized and reported on for the purposes of this study. INFOSAN Members will also be invited to participate in webinars after each phase to learn about the results and next steps.

Are there any risks?

There are no risks anticipated with participating in this study. However, if you experience any distress following participation you are encouraged to inform the researcher and contact the resources provided at the end of this sheet. Neither participating nor abstaining will impact your future treatment as an INFOSAN member or the technical support provided to you or your country by the WHO.

Are there any benefits to taking part?

Although you may find participating interesting, there are no direct benefits in taking part. However, some participants may find it a positive experience to reflect on their role as an INFOSAN member as well as their contributions to a safer food supply, and to participate in research which they may feel is of relevance to them, their institution, their country or the global community.

Who has reviewed the project?

This study has been reviewed and approved by the Ethics Review Committee at the World Health Organization and the Faculty of Health and Medicine Research Ethics Committee at Lancaster University.

Where can I obtain further information about the study if I need it?

If you have any questions about the study, please contact the researcher:

Carmen Savelli
c.savelli@lancaster.ac.uk
+41 22 791 3234

Or his supervisor in the Department of Health Research at Lancaster University:

Dr Céu Mateus
c.mateus@lancaster.ac.uk

Continued on next page…

Continued from previous page…

Or attend the upcoming webinar:

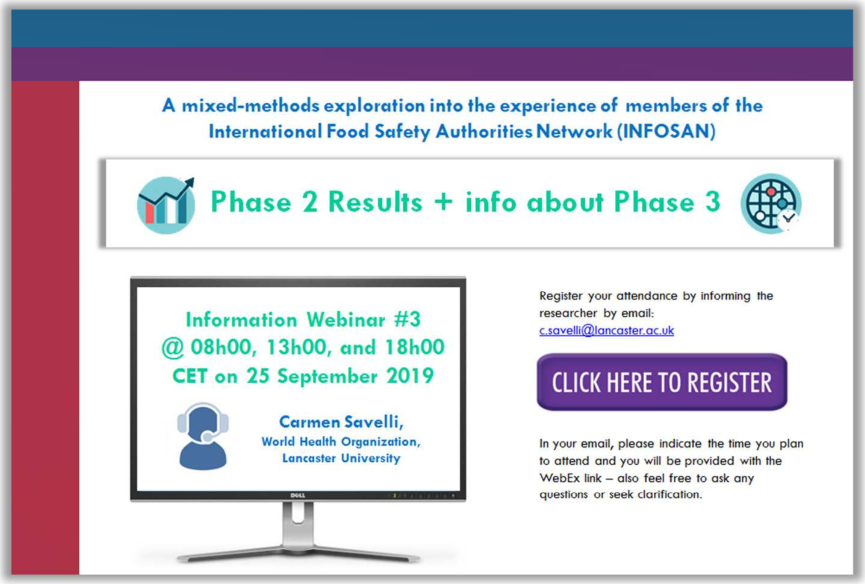

Complaints
If you wish to make a complaint or raise concerns about any aspect of this study and do not want to speak to the researcher or his supervisor, you can contact:

Professor Catherine Walshe
Head of the Division of Health Research
+44 (0)1524 510124
c.walshe@lancaster.ac.uk

Lancaster University
Lancaster
LA1 4YW

If you wish to speak to someone outside of the Public Health Doctorate Programme, you may also contact:

Professor Roger Pickup Tel: +44 (0)1524 593746
Associate Dean for Research Email: r.pickup@lancaster.ac.uk
Faculty of Health and Medicine
(Division of Biomedical and Life Sciences)
Lancaster University
Lancaster
LA1 4YG

Thank you for taking the time to read this information.

Resources in the event of distress
Should you feel distressed either as a result of taking part, or in the future, the following resource may be of assistance:
https://online.supportgroups.com/

Kind regards,

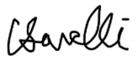

Carmen Savelli

Supplementary File 12 – Email to volunteers for Phase 3 that were not selected

To be sent from the researcher: c.savelli@lancaster.ac.uk

Health & Medicine | Lancaster University

Dear <<Name>>,

Many thanks for your interest in participating in Phase 3 of our study to explore the experiences of members of the International Food Safety Authorities Network (INFOSAN).

We have seen an overwhelmingly positive response to our call for volunteers to fill a limited number of interview slots. Unfortunately, due to resource restrictions including time restraints we will not be able to include you in this phase of the study.

Volunteers were selected on the basis of geographic location (maximum of two participants per WHO region) and length of membership to INFOSAN. If more than two members volunteered from the same region, the two who have been members of INFOSAN the longest were selected. This is what happened in your case.

Please be reminded that your active participation in the INFOSAN community is valued and appreciated and we hope you continue to engage in the important activities of the Network in the future.

If you have any additional information you wish to share regarding your experience as an INFOSAN member, you are always welcome to contact the INFOSAN Secretariat at WHO directly (infosan@who.int) outside of the context of this study.

Kind regards,

CSavelli

Carmen Savelli

Supplementary File 13 – Consent form for volunteers participating in Phase 3

To be sent from the researcher: c.savelli@lancaster.ac.uk

Health & Medicine | Lancaster University

A mixed-methods exploration into the experience of members of the International Food Safety Authorities Network (INFOSAN): Phase 3 consent form

Many thanks for your interest in participating in Phase 3 of our study to explore the experiences of members of the International Food Safety Authorities Network (INFOSAN).

Consent Form

Before you consent to participating in the study we ask that you re-read the participant information (contained in Information Email #4 and attached for ease of reference) and mark each box below with your initials if you agree. If you have any questions or queries before signing the consent form please contact the researcher, Carmen Savelli (c.savelli@lancaster.ac.uk or +41 (0) 79 9456 320).

Initial here

1. I confirm that I have read the participant information and fully understand what is expected of me within this study ☐
2. I confirm that I have had the opportunity to ask any questions and to have them answered. ☐
3. I understand that my interview will be audio recorded and then made into an anonymised written transcript. ☐
4. I understand that audio recordings will be kept until the research project has been examined. ☐
5. I understand that my participation is voluntary and that I am free to withdraw at any time without giving any reason. ☐
6. I understand that once my data have been anonymised and incorporated into themes it might not be possible for it to be withdrawn, though every attempt will be made to extract my data, up to the point of publication. ☐
7. I understand that the information from my interview will be pooled with other participants' responses, anonymised and may be published. ☐
8. I consent to information and quotations from my interview being used in reports, conferences and training events. ☐
9. I understand that the researcher will discuss data with their supervisor as needed. ☐
10. I understand that any information I give will remain confidential and anonymous unless it is thought that there is a risk of harm to myself or others, in which case the principal investigator will need to share this information with their research supervisor. ☐ ☐
11. I consent to Lancaster University keeping written transcriptions of the interview for 10 years after the study has finished. ☐
12. I consent to take part in the above study. ☐

Name of Participant__________________ Signature____________________ Date ___________

Name of Researcher __________________Signature ____________________Date ___________

Supplementary File 14 – Interview Guide for Phase 3

The semi-structured format of the interviews will be conversational in style, allowing the researcher and participant to engage in a dialogue where questions can be modified depending on responses. However, the following questions can serve to start discussions within the bounds of the four themes:

Theme 1) Use of the ICW to support network activities

Preface: The INFOSAN Community Website was launched in 2012 and all members of INFOSAN are registered on there. This online portal is the gateway through which all members can communicate with each other and with the INFOSAN Secretariat.

Questions about experiences:

- How often do you access the INFOSAN Community Website?
- What do you normally use it for?
- Do you think it is supporting the activities of INFOSAN?
- Do you think it could be improved and how?
- Do you think the INFOSAN Secretariat should be posting more frequently on the website?
- What kind of information do you think the INFOSAN Secretariat should be posting there?
- How do you think we might be able to get more members engaged on the website, e.g. involved in discussions, sharing documents, etc.?
- What new features would you like to see?
- Do you think a mobile version of the website would improve participation?

Theme 2) Barriers and facilitators to active participation in INFOSAN

Preface: INFOSAN has an important mandate, to communicate between national government authorities around the world to keep the global food supply safe. However, we know that some members are more active than others. Let's discuss some of the potential barriers to and facilitators to participation.

Questions about experiences:

- Is limited capacity in your country a barrier to participation (i.e. Limited capacity/infrastructure dedicated to addressing food safety; insufficient funds; human resources/expertise; national food control system underdeveloped) and if so, how?
- Is lack of training a barrier (Laboratory analysis; food safety risk assessment; outbreak investigation), and if so, how?
- Does a lack of national food safety standards pose a barrier, and if so, how?
- What is coordination between national authorities like at the national level?
- Does a lack of coordination pose a barrier, and if so, how?
- Are their legal constraints that bar your participation in INFOSAN (e.g. legal implications hinder prompt information sharing; lack of food safety legislation; lack of cooperation from industry, etc.), and if so, how?
- Is food safety a priority in your country? If not, does this pose a barrier, and if so, how?

For peer review only

- Are their perceptions that participating in INFOSAN may have a negative impact on the economy (i.e. through damages to trade or tourism sectors)?
- Is the mandate of INFOSAN clear? If not, how could it be clarified?
- If more members understood their roles and responsibilities better, would that facilitate participations? What would be the best way to do this?
- Have you ever attended any INFOSAN training either online or in person? What did you think about the training? How could the training be improved?
- Do you think language barriers restrict active participation in INFOSAN , and if yes, how so?
- Do you have concerns over confidentiality that limit what you share with INFOSAN?
- How could these concerns be addressed to help facilitate your improved participation?
- Are there any obvious weaknesses of INFOSAN, which if rectified could significantly improve the functioning and thereby the impact of INFOSAN?

Theme 3) Perceptions of network achievements and attainment of objectives (including reducing foodborne illness)

Preface: INFOSAN aims to prevent the international spread of contaminated food and foodborne disease and strengthen food safety systems globally, by: 1) Promoting the rapid exchange of information during food safety incidents/emergencies; 2) Sharing information on important food safety issues of global interest; 3) Promoting partnership and collaboration between countries; and 4) Helping countries strengthen their capacity to manage food safety risks "from farm to table".

Questions about experiences:

- How useful has INFOSAN been in promoting the rapid exchange of information during food safety incidents/emergencies?
- Have you been involved in such exchanges? What was that like? Did you get the information you needed quickly enough to respond and implement risk management measures?
- What have your experiences been like when dealing with the INFOSAN Secretariat?
- Does INFOSAN share information on important food safety issues of global interest that you have found useful?
- Have you shared information with other network members?
- What have your experiences been like in terms of using information you received through INFOSAN or sharing information with others in the network?
- Has INFOSAN promoted partnership and collaboration between countries and networks? If yes, how so? Was it useful?
- What has been your experience when interacting with other INFOSAN members during a food safety emergency?
- Has INFOSAN helped countries strengthen their capacity to manage food safety risks "from farm to table"? If yes, how so?
- How could INFOSAN improve the way it functions in order to better achieve its objectives?

Relevance:

- To what extent are the objectives of INFOSAN still valid?

For peer review only

- Are the activities and outputs of INFOSAN consistent with the overall goal and the attainment of its objectives?
- Are the activities and outputs of INFOSAN consistent with the intended impacts and effects?

Effectiveness:

- To what extent have the objectives been achieved or are likely to be achieved?
- What are the major factors influencing the achievement or non-achievement of the objectives?

Efficiency:

- Were INFOSAN activities cost-efficient?
- Were objectives achieved in a timely manner?
- Were INFOSAN activities delivered in the most efficient way compared to alternatives?

Impact:

- What has been the biggest impact that INFOSAN has made?
- What real difference has INFOSAN made to members? To the safety of the global food supply? To the improvement of global food safety systems?
- How have populations' health been affected?

Sustainability:

- What are the major factors which influence the achievement or non-achievement of the sustainability of INFOSAN?

Theme 4) Assessing value of INFOSAN

Preface: INFOSAN has been in operation since 2004, and this study is the first of its kind to try and determine if the network has value to members in a robust and rigorous way.

Questions about experiences:

- What has participation in INFOSAN been a valuable experience for you? How so?
- Have you been able to connect with other food safety professionals to exchange information related to food safety?
- Has information received through INFOSAN led you to implement risk management measures in your country to prevent foodborne illness? Can you tell me about some specific events?
- Do you believe that participation in INFOSAN has reduced foodborne illness in your country? How so? How do you think this could be measured?
- Do you think participation in INFOSAN will reduce foodborne illness in your country the future? Globally?
- Does the utilization of the INFOSAN Community Website add value to the network activities?
- Do you feel like you are part of a global community? Is that something you value?

Is there anything else about your experience as an INFOSAN Member that you would like to share with me at this time?

For peer review only

Supplementary File 15 – Information Email #5 – Results from Phase 3 and study conclusion (including invitation to attend Webinar #4)

To be sent by the INFOSAN Secretariat (infosan@who.int) on behalf of the researcher (cc: c.savelli@lancaster.ac.uk)

Health & Medicine | Lancaster University

Information Email #5

A mixed-methods exploration into the experience of members of the International Food Safety Authorities Network (INFOSAN): Phase 3 results and study conclusion

---

Dear INFOSAN Member,

My name is Carmen Savelli and in May 2018 I first contacted you to let you know that I was conducting this study as a student in the PhD Public Health programme at Lancaster University, Lancaster, United Kingdom. You may also know me as one of the Technical Officers working as the INFOSAN Secretariat at the World Health Organization (WHO) in Geneva, Switzerland. The study has now concluded.

What was the overall study about?
The purpose of this study is to interrogate INFOSAN in order to describe and explore the experiences of members and better understand the role of the network in mitigating the burden of foodborne illness around the world. The study will examine access to and usage of the INFOSAN Community Website, explore barriers and facilitators to active participation in INFOSAN, determine perceptions about the utility of INFOSAN to mitigate foodborne illness, and scrutinize if and how participation in this network creates value for members.

The study was designed in three phases: Phase 1 has examined access and usage patterns of the INFOSAN Community Website; Phase 2 involved the completion of an online survey by INFOSAN members; and Phase 3 involved individual interviews conducted online using WebEx.

What are the results from Phase 3?

To be inserted

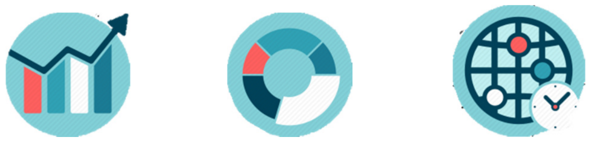

Continued on next page…

Continued from previous page…

How can I find out more about the results from Phase 3 and the overall study conclusion?
Attend the upcoming Webinar:

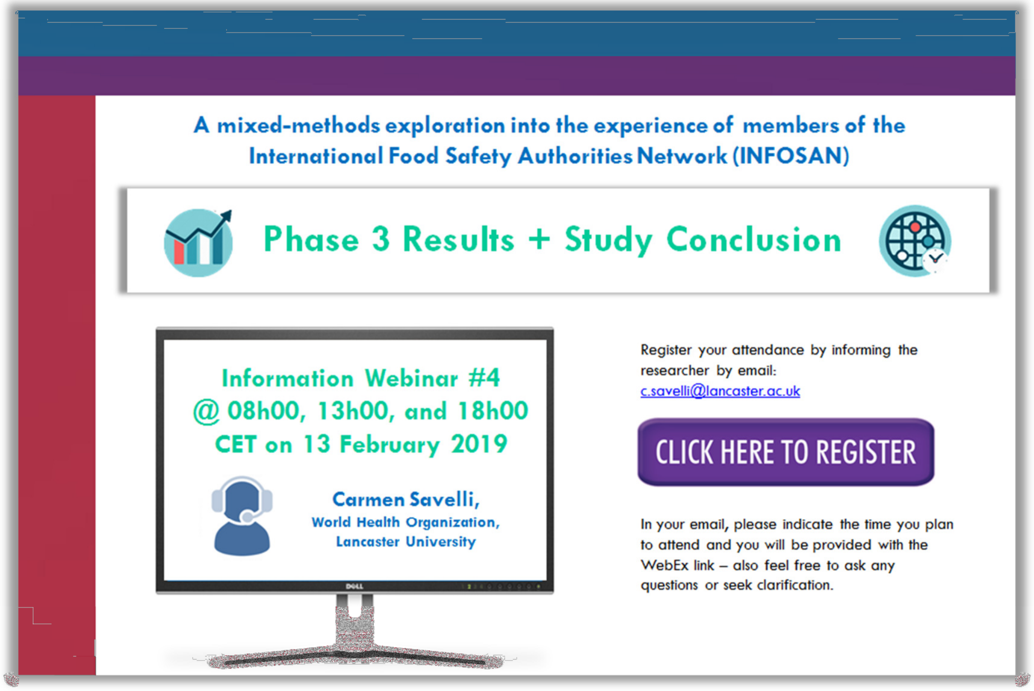

What are the next steps?

The results of this study have been shared with the INFOSAN Secretariat and other senior staff at the World Health Organization for their consideration. Improving the capacity of INFOSAN members to better prevent, detect, and respond to food safety emergencies is a priority.

Any forthcoming publications linked to the study results will be announced by email to the INFOSAN members by the INFOSAN Secretariat.

If you have any additional feedback about this study you are welcome to contact the researcher (c.savelli@lancaster.ac.uk).

If you have other feedback about INFOSAN in general that you wish to share with WHO, please contact the INFOSAN Secretariat directly (infosan@who.int).

Many thanks to all INFOSAN members who contributed time and data to this study. Your participation has been invaluable and is sincerely appreciated.

Kind regards,

Carmen Savelli

Supplementary File 16 - Scientific Review Committee Members

1. **Prof Alan Reilly**, BSc, MTech, CBiol, MIBiol, FIFSTI
   Adjunct Professor, University College Dublin, Institute of Food and Health, Ireland
   → INFOSAN Advisory Group Member
   → Formerly Chief Executive at the Food Safety Authority of Ireland

2. **Dr Jorgen Schlundt**, PhD
   Director and Professor, Nanyang Technological University Food Technology Centre, Singapore
   → INFOSAN Advisory Group Member
   → Formerly Director of the Department of Food Safety and Zoonoses, WHO HQ

3. **Dr Robert Verburg,** PhD
   Associate professor, Faculty of Technology, Policy and Management, Delft University of Technology, Netherlands
   → Developer of the Community Assessment Tool (CAT)

For peer review only

BMJ Open

A mixed-methods exploration into the experience of members of the FAO/WHO International Food Safety Authorities Network (INFOSAN): Study Protocol

Journal:	*BMJ Open*
Manuscript ID	bmjopen-2018-027091.R1
Article Type:	Protocol
Date Submitted by the Author:	25-Feb-2019
Complete List of Authors:	Savelli, Carmen; Organisation mondiale de la Sante, Food Safety and Zoonoses; Lancaster University Faculty of Health and Medicine, Health Research<br>Mateus, Ceu; University of Lancaster, Division of Health Research
**Primary Subject Heading**:	Global health
Secondary Subject Heading:	Global health
Keywords:	Information management < BIOTECHNOLOGY & BIOINFORMATICS, Information technology < BIOTECHNOLOGY & BIOINFORMATICS, World Wide Web technology < BIOTECHNOLOGY & BIOINFORMATICS

SCHOLARONE™
Manuscripts

Title: A mixed-methods exploration into the experience of members of the FAO/WHO International Food Safety Authorities Network (INFOSAN): study protocol

Authors: Carmen Joseph Savelli[1,2], Céu Mateus[2]

1) World Health Organization, Geneva, Switzerland
2) Lancaster University, Lancaster, United Kingdom

Corresponding Author: Carmen Joseph Savelli
World Health Organization
Food Safety and Zoonoses
Avenue Appia 20
1211, Geneva, Switzerland
savellic@who.int
+41 22 79 13234

Key words: international cooperation, foodborne diseases, food safety, networking, World Health Organization

Word Count: 6030

For peer review only

Abstract

Introduction: The International Food Safety Authorities Network (INFOSAN) is a global network of national food safety authorities from 188 countries, managed jointly by FAO and WHO, which facilitates the rapid exchange of information during food safety related events. The proposed research will interrogate INFOSAN in order to describe and explore the experiences of members and better understand the role of the network in mitigating the burden of foodborne illness around the world.

Methods: Examined through a community of practice (CoP) lens, a three-phase research design will combine quantitative and qualitative methods (including website analytics in phase 1, online survey administration in phase 1, and semi-structured interviews in phase 3) to elicit a broad and deep understanding of the network operation and member experiences.

Analysis: In phases 1 and 2, quantitative data collected from the INFOSAN Community Website and the online questionnaires will be analysed using descriptive summary statistics. In Phase 3, Interpretative Phenomenological Analysis (IPA) will be utilized to engage in a dialogue with study participants to explore and describe their lived experiences regarding participation in activities related to INFOSAN. An important aspect of the overall analysis will be triangulation of the information collected from each phase including quantitative indicators and qualitative value stories in order to provide a robust understanding of member experience.

Ethics: This study has undergone ethical review and has received approval from Lancaster University's Faculty of Health and Medicine Research Ethics Committee as well as the World Health Organization's Ethics Review Committee.

Dissemination: Findings from the study will be disseminated as a PhD thesis submitted to Lancaster University. In addition, results of the research shall be submitted for publication to relevant academic or professional conferences and journals or other media including books or websites.

Strengths and limitations of this study

- This study represents the first ever to explore and describe the experiences of all INFOSAN members with respect to their participation in network activities as a means to improve global food safety and prevent foodborne illness, using a range of data collection methods.
- The mixed-method approach will strengthen the credibility of the findings and provide a more complete view and deeper understanding of the experiences of members.
- The main limitation of this study is that the online survey will only be available in English, French and Spanish and interviews will only be conducted in English due to limited resources of the study team.

1. Introduction

In 2015, the WHO reported estimates of the global burden of foodborne diseases for the first time. Together, 31 foodborne hazards are estimated to cause 600-million cases of foodborne disease and 420,000 deaths annually, worldwide[1]. Foodborne diseases are preventable, but ensuring a safe national food-supply requires a robust food control system and coordination among different government sectors responsible for human health, animal health, agriculture, trade and others. In

For peer review only

addition, as a global commodity, food produced in one country can readily cause international outbreaks if contaminated food is exported abroad. Channels of communication on matters of food safety must therefore be well established within and between countries in order to prevent national and international food safety emergencies.[2] It is for these reasons why the WHO launched INFOSAN in 2004. Today, the overall aim of INFOSAN is to halt the international spread of contaminated food, prevent foodborne disease outbreaks, and strengthen food safety systems globally[3]. The four main objectives of INFOSAN are to: 1) Promote the rapid exchange of information during food safety related events; 2) Share information on important food safety related issues of global interest; 3) Promote partnerships and collaboration between countries, and between networks; and 4) Help countries strengthen their capacity to manage food safety emergencies. Using the INFOSAN Community Website (ICW; a secure, online portal), INFOSAN members from around the world exchange information on urgent food safety issues and emerging trends of potential global interest. The ICW also provides a virtual environment to share lessons-learned and allows members to pose questions to one another for the purpose of building knowledge related to food safety.[3]

INFOSAN members have a common identity that is defined by a shared domain of interest. By joining the network, each has committed to taking actions that contribute to a safer global food supply by engaging in joint activities and discussions to facilitate knowledge transfer and exchange (KTE). Common responsibilities are also shared by members, as defined by the INFOSAN Secretariat. Combined, these common responsibilities and activities create a sense of community, and are undertaken with the intention of facilitating the application of best practices to improve food safety. In addition, INFOSAN members are each practitioners in their respective countries, some as food regulators, or risk analysts, or epidemiologists or other professionals. While their focus may be different, the uniting factor is that their practice, in some respect, aims to make contributions towards the reduction of foodborne illness. It is the shared domain, community, and practice which allows for INFOSAN to be understood as a Community of Practice (CoP).[4] A CoP is a group of people sharing a particular concern, problem or passion for an area and who deepen their knowledge and expertise by learning from one another and interacting on a regular basis.[4] Such interactions may occur in person or through technology-mediated means, as with INFOSAN.

For peer review only

A growing body of research suggests that KTE can be effectively fostered within CoPs, leading to the uptake and application of best practices by individuals and teams in various sectors including health, business, and beyond.[5] Multiple systematic reviews[6, 7, 8,9] suggest that fostering a virtual or electronic CoP among professionals in public health is useful for encouraging KTE which translates into adoption of evidence-based best practices, and by extension, improved public health. Rajic et al.[10] have described the benefits of facilitating KTE among food safety professionals working at the intersection of agriculture and health. Together, the literature suggests that a CoP like INFOSAN, connecting food safety and public health professionals from around the world, is an appropriate tool to facilitate KTE in this area. However, while INFOSAN has been operating for nearly 15 years to facilitate the aforementioned activities among its members, it has never been characterized or examined as a functional CoP and its value, as understood from the perspective of its members, has never been determined in a systematic or rigorous way. Furthermore, a paucity of research has been conducted to investigate the attributes and effectiveness of specific tools or CoPs like INFOSAN to facilitate cross-border communication during international food safety events. To date, most of the publications mentioning such tools focus on summarising a particular incident response, rather than explicitly examining the tools that were utilised. However, such reports of international food safety events commonly conclude with recommendations to better utilise existing international networks and communication tools to improve and expedite information exchange.[11, 12, 13, 14, 15, 16, 17] In addition, several published studies have specified the important role that INFOSAN has played in facilitating rapid international communication between government officials that led to the timely implementation of risk management measures during a food safety emergency.[18, 19, 20]

3. Research Aim

The overall aim is to explore and describe the experiences of INFOSAN members with respect to their participation in network activities as a means to improve global food safety and prevent foodborne illness.

4. Research Objectives

1) Assess the functioning of INFOSAN as a community of practice by obtaining systematic insights into the characteristics, performance and opinions of members.

For peer review only - http://bmjopen.bmj.com/site/about/guidelines.xhtml

2) Gain a broad and deep understanding of members' perceptions of the utility of INFOSAN as a global communication tool for KTE and the prevention of foodborne illness in their respective country.
3) Determine if participation in INFOSAN creates value for members and explore the mechanisms through which this may occur.

5. Main research questions

1) How is the ICW being used to support the network activities?
2) What are the barriers and enablers to active participation in INFOSAN?
3) Do members of INFOSAN believe that participation in the network has prevented (or will prevent) foodborne illness in their country?
4) Does participation in INFOSAN create value for members and if so, through what mechanisms does this occur?

6. Methodology and methods

6.1. Philosophical underpinnings

The theoretical framework for exploring experiences in CoPs proposed by Wenger et al. [21] (2011) is utilized for this study and has its roots in critical realism. Critical realism is a philosophical perspective that accepts the existence of stable and enduring features of reality independently of one's ability to perceive them and therefore should be measured as a sum of different perspectives.[22] This concept represents the ontological perspective of critical realism (where ontology should be understood as the philosophical study of being). Furthermore, critical realism asserts that social phenomena and their meanings are continually being created by social actors (i.e. the philosophical concept of constructivism) and may differ depending on one's perspective (i.e. the philosophical concept of relativism). Constructivism and relativism represent the epistemic orientation of critical realism (where epistemology is the philosophical study of knowledge and how one knows).[23]

The importance of utilizing quantitative indicators together with qualitative narratives from different perspectives in a community is a concept described by Wenger et al.[21] in order to understand

For peer review only

what kind of experiences create value within CoPs. Quantitative indicators can be readily obtained from most virtual CoPs by measuring the number of unique users logging in to a web portal over time or the number of postings within a discussion forum, for example. However, Wenger et al.[21] suggest that relying on such indicators alone would require too many assumptions to be made to accurately find meaning in experiences and determine value. Such indicators should rather serve as a point of reference from which value-creation stories can be elicited from members directly in order to provide a more robust understanding of experience. Likewise, examining only qualitative narratives from members would ignore the opportunity to crosscheck information with indicators to determine how perceptions and actions correspond. It is this acknowledgement of multiple perspectives of reality which demonstrates congruence with critical realism. In taking this approach, discrepancies and correspondences can be observed and both grounded narratives and aspirational narratives become illuminated (where grounded narratives are those for which value-creation stories are supported by quantitative indicators and aspirational narratives are not).[21]

With an understanding of INFOSAN as a functional CoP, and with an appreciation for the critical realist perspective that underpins its examination, it is incumbent upon the researcher of the proposed study to take a mixed-method approach in order to strengthen the credibility of the findings and provide a more complete view and deeper understanding of the experiences of members. The utilization of such an approach for the investigation of experiences of online community members is strongly supported by a growing body of literature.[24, 25, 26]

6.2. Setting

The study will be conducted by the researcher from within the Department of Food Safety and Zoonoses (FOS) at the headquarters of the WHO in Geneva, Switzerland (where the INFOSAN Secretariat is based), however the true setting is global since INFOSAN membership spans 188 countries.

6.3 General comments on sampling

For each phase of the research, participants will only include registered INFOSAN members. These individuals have been officially designated by their national government and are all registered

For peer review only

on the ICW (N=500). INFOSAN membership includes both men and women in approximately equal proportions.

6.4 General comments on recruitment

Written permission to conduct the proposed research on INFOSAN has been received from the Director of FOS at the WHO, where the INFOSAN Secretariat is based. To announce the launch of the study, all members of INFOSAN will receive by email, written introductory information about the proposed research (Information Email #1; supplementary file #1), including an invitation to attend an online seminar (i.e. webinar), delivered by the researcher, to find out more information about the study and ask any questions or seek clarification. This webinar, like all future webinars discussed in this proposal, will be scheduled at three different times to allow INFOSAN members across different time zones to participate. The webinar will be delivered using the secure online conferencing tool, WebEx. The live webinars will not be recorded, but a recording will be made by the researcher alone and will be made available to INFOSAN members to view in case they would like to do so at their convenience.

6.5. Phase 1: Analysis of INFOSAN Community Website access and usage

6.5.1. Sampling, recruitment and consent

Information Email #1 will explain the three different phases of the proposed research, and remind INFOSAN members that data analysed in Phase 1 of the study will be extracted from the ICW in accordance with the Terms and Conditions of Use that each member consented to when they registered online (supplementary file #2). The relevant text within the Terms and Conditions of Use, reads as follows: "Utilization of website analytics tools or other methods will be applied periodically to summarize members' access to and usage of the INFOSAN Community Website for monitoring, evaluation and research purposes." As such, data pertaining to all members of INFOSAN with respect to their access to and usage of the ICW, will be utilised in Phase 1 of the study unless a member expressly indicates their wish to be excluded. Information Email #1 (and related webinars) will ensure that INFOSAN members understand that any members not wishing to have their website access and usage data utilised for the purpose of this study will have two weeks to make this indication by email to the researcher. All those members who do not otherwise object will be recruited for Phase 1. Those

For peer review only

who are recruited in Phase 1 will have a further two weeks to opt out of the study, after which time this will no longer be possible due to aggregation of the data.

6.5.2. Data collection and analysis

Access to the ICW is granted to the researcher in his capacity as a staff member at WHO and approval for use in this research has been granted by the Director of FOS, WHO. Data from the ICW will be collected retrospectively for the period between February 2012 (when the website was launched) and December 2018. Information from all recruited members concerning the following variables will be downloaded from the website, anonymised and exported into Microsoft Excel and SPSS for analysis: type of member, sex, country, languages spoken, government sector, primary function (i.e. risk assessment, risk communication, or risk management), and areas of scientific expertise. These data have all been automatically collected and stored in the internal website database at the time of member registration. Additional data about length of membership, last access to the website, and discussion thread initiations, responses and views will also be collected and exported for analysis. Once collected, all anonymised data will be analysed using descriptive summary statistics, which will allow for stratification by a number of variables including type of member, geographic region, and length of membership. Analysing these data will provide an objective, foundational layer of information about the experiences of all members and will be triangulated with data from Phases 2 and 3 to determine if members' reported attitudes and experiences reflect their online behaviours. In addition, triangulated data will also allow for INFOSAN to be described with respect to its stage of community development according to Wenger et al.[4], taking into account its structuring characteristics as described by Dubé, Bourhis and Jacob.[27]

6.6. Phase 2: Administration of an online survey

6.6.1. Sampling, recruitment and consent

Upon conclusion of data collection and analysis in Phase 1, all members of INFOSAN will receive Information Email #2 (supplementary file #3) which will include indicative results from Phase 1 and an invitation to attend a webinar, delivered by the researcher, to learn more about the results from Phase 1 and to provide further details about Phase 2. Immediately following the webinar, all INFOSAN members (N=500) will receive Information Email #3 (supplementary file #4), including

For peer review only

the invitation to participate in Phase 2 of the study and containing a link to an online survey. Only those who express consent will be recruited as participants for Phase 2. No consent form will be collected because that would compromise anonymity, however, the email will explain that by clicking on the link, the individual confirms that they have read the introductory information and understand what is expected of them as a participant in this phase of the study (full details in supplementary file #4).

6.6.2. Data collection and analysis

Recruited participants will be requested to complete an online questionnaire that will take between 30 and 45 minutes. Responses to the questionnaire are intended to provide systematic insights into the characteristics, performance and opinions of INFOSAN members and contribute to a broader understanding of their experiences. The questionnaire will consist of questions from the Community Assessment Toolkit (CAT)[27] as well as a supplemental set of questions, tailored specifically to INFOSAN members. During the development of the CAT, Verburg and Andriessen[27] demonstrated that the methodology for its development was based on dominant theories of CoPs and group dynamics. The CAT was pilot tested and studied with seven CoPs (n=271) to enable reliability tests and scale analysis. Cronbach's alpha for the scales ranged from .59 to .91 and as the items referred to quite separate goals, Verburg and Andriessen[27] reported these values acceptable. Utilizing the CAT in this study will enable future comparative research between CoPs that have been assessed with the same tool (e.g. Roberts[25]). However, given the unique nature of INFOSAN and the specific objectives of this study, it is also necessary to develop a short set of supplemental questions to examine the experiences of INFOSAN members that are unique to this particular CoP. A preliminary set of supplemental questions has been inserted to the appropriate sections of the CAT questionnaire in supplementary file #5. These supplemental questions will be reviewed for content validity by a panel of experts consisting members of the INFOSAN Advisory Group since they are familiar with the constructs that the supplemental questions are designed to measure. The expert panel will judge whether the supplemental questions adequately measure the construct they are intended to assess, and whether these supplemental questions are indeed sufficient to measure the domain of interest. A Content Validity Index (CVI) will be computed for each supplementary item and items with a CVI of

For peer review only

0.78 or higher for three or more experts will be considered evidence of good content validity.[28] Based on feedback from the expert panel, the supplemental questions will be revised prior to pilot testing. After incorporating any suggestions from INFOSAN Advisory Group members, FAO/WHO Regional Food Safety Advisors/Officers will be requested to pilot test the supplemental questions. As a measure of reliability, internal consistency will be estimated using the coefficient alpha, also known as Cronbach's alpha. Determining the internal consistency will reflect the extent to which the questionnaire items are inter-correlated, or whether they are consistent in measurement of the same construct.[29]

Because the questionnaire (including the CAT questions and the supplementary questions) will be disseminated to INFOSAN members in 188 countries, it will be adapted from English into French and Spanish in order to encourage a higher response rate. The aim of the adaptation process is to achieve different language versions of the English instrument that are conceptually equivalent in both French and Spanish. The instrument should be equally natural and acceptable and should practically perform in the same way, with a focus on cross-cultural and conceptual equivalence, rather than on linguistic/literal equivalence. A well-established method to achieve this goal is to use forward-translations and back-translations.[30] This method has been refined over the course of several WHO studies to result in the following process which will be undertaken to adapt the questionnaires into French and Spanish.[30] First, one native Spanish speaker and one native French speaker from the Department of Food Safety and Zoonoses at WHO will conduct forward translations of the entire questionnaire. These translators will be health professionals, familiar with the terminology of the area covered by the instrument. Next, two bilingual expert panels will be convened to identify and resolve the inadequate expressions/concepts of the translation, as well as any discrepancies between the forward translation and the English versions of the questions. Next, using the same approach as outlined in the first step, the questionnaire will then be translated back to English by an independent translator (from central translation services at WHO), whose mother tongue is English and who has no knowledge of the questionnaire. As in the forward-translation, emphasis in the back-translation should be on conceptual and cultural equivalence and not linguistic equivalence. Discrepancies should be discussed with the researcher and further work (forward translations, discussion by the bilingual

For peer review only

expert panel, etc.) will be iterated as many times as needed until a satisfactory version is reached. This version will then be pre-tested with a group of 20 interns at WHO including 10 native French speakers and 10 native Spanish speakers. A call for volunteers will be disseminated through the WHO intern mailing list. Pre-test respondents will be administered the questionnaire and then systematically debriefed. This debriefing will ask respondents what they thought the questions were asking, whether they could repeat the questions in their own words, and what came to their minds when they heard a particular phrase or term. The debriefing will take the form of a focus group, organized at WHO. The adaptation process followed will be traceable through a number of documents including: 1) initial forward versions; 2) a summary of recommendations by the expert panels; 3) the back-translations; 4) a summary of problems found during the pre-testing of the instrument and the modifications proposed; 5) the final version; and 6) a description of the samples used in this process (i.e. the composition of the expert panel and the pre-test respondent samples). The process of expanding and adapting the questionnaire is summarized in supplementary file #6.

Quantitative data collected from the online questionnaires will be analysed using descriptive summary statistics, allowing for stratification by a number of variables including type of member, geographic region, length of membership, etc. A variety of techniques for univariate, bivariate and multivariate analysis using SPSS will be employed in order to examine patterns and relationships between variables. Depending on the response rate to the survey, the researcher may need to adjust for non-response bias in order to generalise the results to the entire network.[31] The researcher is a native English speaker with a working knowledge of French and basic knowledge of Spanish. As such, for the very few instances where an open text response is an open, answers provided in French or Spanish will be translated by the researcher with the aid of Google translate, if necessary.

6.7. Phase 3: Semi-structured interviews

6.7.1. Sampling, recruitment and consent

Upon conclusion of data collection and analysis in Phase 2, all members of INFOSAN will receive Information Email #4 (supplementary file #7) which will include indicative results from Phase 2 and an invitation to attend a webinar, delivered by the researcher, to learn more about the results from Phase 2 and to provide further details about Phase 3. The email and webinar will also include a

For peer review only

request that members interested in participating in Phase 3 indicate this by emailing the researcher. The online survey in Phase 2 will also serve as a recruitment tool for Phase 3 since a concluding statement on the survey will indicate that members interested in participating in Phase 3 of the research should contact the researcher separately by email.

A minimum of six and a maximum of 12 participants will be included in the study sample, aiming for two participants from each of the six geographic regions delineated by WHO. The purpose is not to be representative of the entire network, however, because INFOSAN is global, including participants from each region may reveal a richer pool of experience than if all members were selected from a single region. In addition, a sample size within this range should allow for the examination of similarities and differences between individuals, without producing an overwhelmingly large amount of qualitative data that cannot be managed within the confines of the study timeline. This sample of participants will be restricted to those INFOSAN members who have been registered members for a minimum of two years at the time of their interview to ensure they have a reasonable level of experience with the network from which to draw. The sample will be limited to those members who speak English due to limited funding for research conduct (including for translation and interpretation) and limited time for collecting and analysing data in other languages. In the event that at least one member per WHO region does not volunteer after receiving Information Email #4, follow-up emails will be sent to those members from the regions where volunteers are still needed, to indicate as such.

If more than two people from a single WHO region indicate their interest in participating in Phase 3, the two people who have been members the longest will be selected and notified by email from the researcher, providing they are not from the same country. Any individuals who volunteer for Phase 3 but who are not selected will be emailed individually by the researcher and provided with an explanation about how the selection was made (supplementary file #8).

Prior to commencing their interview, INFOSAN members who volunteer and are recruited for Phase 3 of the study will need to have read, signed, and returned by email to the researcher, the Phase 3 consent form as detailed in supplementary file #9. Immediately before the interview, the researcher

For peer review only

will ensure the participants are aware they can choose to withdraw from the study at any time, for any reason, before or during their interview and withdraw their data up to two weeks after their interview.

6.7.2. Data Collection and Analysis

Recruited participants will be requested to participate in a semi-structured interview conducted online using the secure tool, WebEx, because participants are anticipated to be located in various countries around the world (no face-to-face interviews will be conducted). The interviews will be scheduled to last between 45 minutes and one hour. The same four themes explored in Phase 2 will be explored during the interviews, and the discussion will focus on answering the related questions of 'how?' and 'why?' instead of just 'what?' as elaborated in the interview guide in supplementary file #10 (results from Phase 1 and/or 2 may lead to the addition or deletion of certain questions from the current interview guide). The researcher will follow Interpretative Phenomenological Analysis (IPA) to engage in a dialogue with study participants to explore and describe their lived experiences regarding participation in activities related to INFOSAN. Such a method requires a flexible data collection instrument[32] and therefore, the semi-structured format of the interviews will be conversational in style, allowing the researcher and participant to engage in a dialogue where questions can be modified depending on responses. This format also enables the researcher to prompt further elaboration in certain areas of interest identified by participants, allowing for more flexibility than a structured interview.[32] The interviews will be audio recorded and then transcribed by the researcher. Audio recordings will be made using WebEx and immediately downloaded, password protected and encrypted on a laptop. Audio recorded data will be anonymised as far as is possible (given the nature of audio data) by saving the file with an de-identified tag (e.g. Participant #1 Region X). Prior to recording, the researcher will remind the participant that they can refrain from using names of people and places to the extent possible when answering questions, to assist with anonymization. Once recording has started, the researcher will not use the participant's name during the interview. Transcripts will be anonymized by replacing identifying names of people or places with a de-identified tag (e.g. Participant #1 Region X). Analysis will involve multiple readings of the interview transcripts, coupled with note taking, followed by transformation of notes into emergent themes, and finally, seeking relationships and clustering themes.[32]

For peer review only

An important aspect of the overall analysis will be triangulation of the information collected from each phase including quantitative indicators and qualitative value stories. Anonymised information and quotations from participant interviews will be reported, representing the limits to confidentiality. The combined strengths of quantitative and qualitative methods can contribute to improved study validity, credibility, and overall integrity and provide a broad and deep understanding of members' experiences.[31, 33, 34] The indicative results from Phase 3, along with a triangulated analysis of data from all three phases will be communicated to INFOSAN members through Information Email #5 (supplementary file #11) and a final webinar, delivered by the researcher.

7. Patient and Public Involvement

Patients were not involved in the design of this study. The first author has had conversations with several INFOSAN members in finalizing this study design. These conversations have followed a presentation of the research design at a regional meeting of INFOSAN members in Miami, USA in November 2017 where INFOSAN members from approximately 30 countries were present. In addition, a more detailed presentation of the study design was delivered at a meeting of 8 INFOSAN members in Geneva, Switzerland in December 2017, where further discussion has contributed to the finalization of the study design, including the overall aim, objectives and research questions. The questionnaire will also be vetted by several target participants (selected because they are also part of the INFOSAN Advisory Group) as described in supplementary file #6. The results of each phase of the study will be communicated to study participants via email and through webinars delivered by the first author.

8. – Ethical issues

8.1. – Data management and storage

In accordance with the General Data Protection Regulation (GDPR) and the (UK) Data Protection Act 2018, all electronic data will be anonymised and stored onto encrypted password-protected storage devices, including data keys and a laptop computer) provided by the WHO. These encrypted files will utilise meaningful file names and version numbers and will be accompanied by a

For peer review only

Readme file. Personal identifiers will be kept separately from anonymised data in either encrypted computer storage (for electronic files) or in a locked filing cabinet (for paper files) and destroyed following the completion and acceptance of the PhD thesis. Only the researcher and his supervisors will have access to the anonymised data for the duration of the study. Electronic copies of transcripts will be transferred in person by the researcher from WHO headquarters to Lancaster University on encrypted, password-protected storage devices and then archived for 10 years in secure encrypted storage on the Lancaster University server, after which time they will be destroyed. Storage and destruction of data will be done by Lancaster University's designated Data Protection Officer. The researcher may need to modify the aforementioned arrangements related to data management and storage and privacy protection measures to ensure that provisions of the GDPR are accommodated in case such provisions are supplemented, interpreted or clarified while the study is being conducted.

8.2. – Conducting insider research

By the nature of this work-based research project, the researcher is an insider, investigating an issue that examines, in broad terms, the operation of an organizational programme. The researcher is therefore an agent of his organization as a technical officer at WHO and also an agent of Lancaster University as a PhD student. As such, the ethical considerations for the design of this research project have been carefully made from the insider researcher perspective.

In addition to approval being granted by the Director of FOS to conduct this research, the proposed study has been subject to scrutiny and approval by the Faculty of Health and Medicine Research Ethics Committee at Lancaster University and the WHO Research Ethics Board before it could commence. This process involved technical review by an external scientific committee of experts. In addition, the conduct of the research will be governed by the WHO Code of Ethics and Professional Conduct and the WHO Code of Conduct for Responsible Research which both emphasise the need for all research to be conducted with integrity, accountability, independence, impartiality, respect, and professional commitment.

While there are several positive aspects to being an insider researcher (e.g. informed perspective, ability to implement study recommendations directly, etc.), the potential conflicts of interest must be carefully considered, acknowledged and addressed. As members of INFOSAN are

For peer review only

familiar with the researcher in his role at WHO, he will need to ensure transparency and clarity that the proposed research is being conducted as part of a PhD study. It will also be necessary to ensure that despite having access to additional data or information, that only those data collected with the expressed consent of participants is utilised and reported on for the purposes of this study. INFOSAN members must be assured that neither their participation nor abstention will impact their future treatment as an INFOSAN member or the technical support provided to them or their agency by the WHO.

Acknowledging the role of the researcher as an insider is congruent with the methodology used to conduct this research on INFOSAN through a CoP lens. IPA as a research methodology is concerned with carefully detailing the lived experience of individuals. As explained by Guldberg and Mackness[26], utilizing IPA to understand experience aligns with a CoP lens since the focus of analysis is on the interpretations of members and the values they attribute to them. In addition, IPA acknowledges and embraces the role of the researcher's interpretation and understanding of members' lived experiences. The researcher, through his insider role on the INFOSAN Secretariat, plays an important part in connecting, communicating and facilitating interaction among members, and therefore his familiarity with the participants and ability to provide expertise in the interpretation and understanding of members' experiences should be considered an asset.

While the role of insider researcher can present some potential conflicts of interest, transparency in process, due permissions from senior WHO staff, and assurances given to INFOSAN members, should ensure this research is conducted to the highest ethical standard. In addition, several techniques well-known to insider researchers will be employed to aid in accurately understanding and documenting the experiences of INFOSAN members, including the practice of reflexivity. The practice of reflexivity will involve active engagement of the self and questioning of the researcher's own perceptions in order to expose their contextualized nature.[35] A diary will be kept by the researcher during data collection and analysis to document reflexivity including predictions of outcomes for each phase of the study and how they relate to one another. The diary will not include any identifying information related to participants to ensure anonymity of participants and any reflections on interviewed participants will reference a de-identified tag (e.g. Participant #1 Region

For peer review only

X). The diary will be stored in a locked cabinet in the main researcher's office when not being used in case it contains any sensitive information.

9. Dissemination plans

Results from this study can be utilized directly by the INFOSAN Secretariat at WHO in order to plan future interventions intended to encourage and support active participation in this important global network. In addition, results from this study may also contribute new knowledge regarding the coordination of international virtual networks and communities of practice in the realm of global health. The researcher will therefore disseminate findings from the study in the following ways: 1) via webinars, open to all INFOSAN members following each of the three phases of the study; 2) as an oral presentation to staff within FOS, WHO; and 3) as a PhD thesis submitted to Lancaster University. In addition, results of the research shall be submitted for publication to relevant academic or professional conferences and journals or other media including books or websites.

Authors' contributions: CS conceived the original idea, designed the study, drafted the manuscript and approved the final document. CM drafted the manuscript and approved the final document. CS is a staff member of the World Health Organization. The authors alone are responsible for the views expressed in this publication and they do not necessarily represent the views, decisions or policies of the World Health Organization.

Funding statement: This study is funded by the World Health Organization.

Competing interests: None

For peer review only

References

1. World Health Organization. (2015). *Global burden of foodborne disease report*. Retrieved from: http://apps.who.int/iris/bitstream/10665/200046/1/WHO_FOS_15.02_eng.pdf?ua=1.

2. World Health Organization. (2014). *Advancing food safety initiative: strategic plan for food safety including foodborne zoonoses 2013-2022*. Retrieved from: http://www.who.int/foodsafety/publications/strategic-plan/en/.

3. World Health Organization. (2016). *INFOSAN Activity Report 2014/2015*. Retrieved from: http://apps.who.int/iris/bitstream/10665/246204/1/9789241510592-eng.pdf.

4. Wenger, E., McDermott, R. and Snyder, W. (2002). *Cultivating communities of practice*. Boston, Mass.: Harvard Business School Press.

5. Ho, K., Jarvis-Selinger, S., Norman, C., Li, L., Olatunbosun, T., Cressman, C., & Nguyen, A. (2010). Electronic communities of practice: Guidelines from a project. *Journal Of Continuing Education In The Health Professions*, *30*(2), 139-143. http://dx.doi.org/10.1002/chp.20071

6. Kothari, A., Hovanec, N., Hastie, R., & Sibbald, S. (2011). Lessons from the business sector for successful knowledge management in health care: A systematic review. *BMC Health Services Research*, *11*(1). http://dx.doi.org/10.1186/1472-6963-11-173

7. Mairs, K., McNeil, H., McLeod, J., Prorok, J., & Stolee, P. (2013). Online strategies to facilitate health-related knowledge transfer: a systematic search and review. *Health Information & Libraries Journal*, *30*(4), 261-277. http://dx.doi.org/10.1111/hir.12048

8. Quinn, E., Huckel-Schneider, C., Campbell, D., Seale, H., & Milat, A. (2014). How can knowledge exchange portals assist in knowledge management for evidence-informed decision making in public health?. *BMC Public Health*, *14*(1). http://dx.doi.org/10.1186/1471-2458-14-443

9. Barbour, L., Armstrong, R., Condron, P., & Palermo, C. (2018). Communities of practice to improve public health outcomes: a systematic review. Journal Of Knowledge Management, 22(2), 326-343. doi: 10.1108/jkm-03-2017-0111

10. Rajić, A., Young, I., & McEwen, S. (2013). Improving the Utilization of Research Knowledge in Agri-food Public Health: A Mixed-Method Review of Knowledge Translation and Transfer. *Foodborne Pathogens And Disease*, *10*(5), 397-412. http://dx.doi.org/10.1089/fpd.2012.1349

11. Einöder-Moreno, M., Lange, H., Grepp, M., Osborg, E., Vainio, K., & Vold, L. (2016). Non-heat-treated frozen raspberries the most likely vehicle of a norovirus outbreak in Oslo, Norway, November 2013. *Epidemiology And Infection*, *144*(13), 2765-2772. http://dx.doi.org/10.1017/s0950268816000194

12. Guzman-Herrador, B., Nilsen, E., Cudjoe, K., Jensvoll, L., Kvamme, J., & Lindegård Aanstad, A. et al. (2013). A Shigella sonnei outbreak traced to imported basil – the importance of good typing tools and produce traceability systems, Norway, 2011. *Eurosurveillance*, *18*(49), 20650. http://dx.doi.org/10.2807/1560-7917.es2013.18.49.20650

13. Inns, T., Ashton, P., Herrera-Leon, S., Lighthill, J., Foulkes, S., & Jombart, T. et al. (2016). Prospective use of whole genome sequencing (WGS) detected a multi-country outbreak of Salmonella Enteritidis. *Epidemiology And Infection*, *145*(02), 289-298. http://dx.doi.org/10.1017/s0950268816001941

For peer review only

14. Knoblauch, A., Bratschi, M., Zuske, M., Althaus, D., Stephan, R., & Hächler, H. et al. (2015). Cross-border outbreak of Salmonella enterica ssp. enterica serovar Bovismorbificans: multiple approaches for an outbreak investigation in Germany and Switzerland. *Swiss Medical Weekly*. http://dx.doi.org/10.4414/smw.2015.14182

15. Nygård, K., Lassen, J., Vold, L., Andersson, Y., Fisher, I., & Löfdahl, S. et al. (2008). Outbreak of Salmonella Thompson Infections Linked to Imported Rucola Lettuce. *Foodborne Pathogens And Disease*, *5*(2), 165-173. http://dx.doi.org/10.1089/fpd.2007.0053

16. Pezzoli, L., Elson, R., Little, C., Yip, H., Fisher, I., & Yishai, R. et al. (2008). Packed with Salmonella —Investigation of an International Outbreak of Salmonella Senftenberg Infection Linked to Contamination of Prepacked Basil in 2007. *Foodborne Pathogens And Disease*, *5*(5), 661-668. http://dx.doi.org/10.1089/fpd.2008.0103

17. Rebolledo, J., Garvey, P., Ryan, A., O'donnell, J., Cormican, M., & Jackson, S. et al. (2013). International outbreak investigation of Salmonella Heidelberg associated with in-flight catering. *Epidemiology And Infection*, *142*(04), 833-842. http://dx.doi.org/10.1017/s0950268813001714

18. Acciari, V., Iannetti, L., Gattuso, A., Sonnessa, M., Scavia, G., Montagna, C., et al. (2015). Tracing sources of Listeria contamination in traditional Italian cheese associated with a US outbreak: investigations in Italy. *Epidemiology And Infection*, *144*(13), 2719-2727. http://dx.doi.org/10.1017/s095026881500254x

19. Gossner, C., Schlundt, J., Ben Embarek, P., Hird, S., Lo-Fo-Wong, D., & Beltran, J. et al. (2009). The Melamine Incident: Implications for International Food and Feed Safety. *Environmental Health Perspectives*, *117*(12), 1803-1808. http://dx.doi.org/10.1289/ehp.0900949

20. Khardori, N. (2012). A Multistate Outbreak of Hepatitis A Associated With Semidried Tomatoes in Australia, 2009. *Yearbook Of Medicine*, *2012*, 101-102. http://dx.doi.org/10.1016/j.ymed.2012.08.004

21. Wenger, E., Trayner, B., and de Laat, M. (2011). Promoting and assessing value creation in communities and networks: a conceptual framework. Rapport 18, Ruud de Moor Centrum, Open University of the Netherlands.

22. Fade, S. (2004). Using interpretative phenomenological analysis for public health nutrition and dietetic research: a practical guide. *Proceedings Of The Nutrition Society*, *63*(04), 647-653. http://dx.doi.org/10.1079/pns2004398

23. Maxwell, J. (2012). *A realist approach for qualitative research*. Thousand Oaks, CA: SAGE.

24. de Laat, M., & Lally, V. (2003). Complexity, theory and praxis: Researching collaborative learning and tutoring processes in a networked learning community. *Instructional Science*, 31, pp. 7-39.

25. Roberts, G. (2015). Communities of practice: Exploring enablers and barriers with school health clinicians. *Canadian Journal Of Occupational Therapy*, *82*(5), 294-306. http://dx.doi.org/10.1177/0008417415576776

26. Guldberg, K., & Mackness, J. (2009). Foundations of communities of practice: enablers and barriers to participation. *Journal Of Computer Assisted Learning*, *25*(6), 528-538. http://dx.doi.org/10.1111/j.1365-2729.2009.00327.x

27. Verburg, R., & Andriessen, J. (2006). The assessment of communities of practice. *Knowledge And Process Management*, *13*(1), 13-25. http://dx.doi.org/10.1002/kpm.241

For peer review only

28. Dube, L., Bourhis, A., & Jacob, R. (2006). Towards a Typology of Virtual Communities of Practice. Interdisciplinary Journal Of Information, Knowledge, And Management, 1, 069-093. doi: 10.28945/115

28. Polit, D., Beck, C., & Owen, S. (2007). Is the CVI an acceptable indicator of content validity? Appraisal and recommendations. *Research In Nursing & Health*, *30*(4), 459-467. http://dx.doi.org/10.1002/nur.20199

29. Tsang, S., Royse, C., & Terkawi, A. (2017). Guidelines for developing, translating, and validating a questionnaire in perioperative and pain medicine. *Saudi Journal Of Anaesthesia*, *11*(5), 80. http://dx.doi.org/10.4103/sja.sja_203_17

30. World Health Organization. (2018). *Process of translation and adaptation of instruments*. Retrieved from: http://www.who.int/substance_abuse/research_tools/translation/en/.

31. Bryman, A. (2012). *Social research methods (4th Edition)*. Oxford: Oxford University Press.

32. Smith, J, Flowers, P, & Larkin, M. (2009). *Interpretative phenomenological analysis: Theory, method and research*. Thousand Oaks, California: Sage.

33. Allsop, J. (2013). *Researching health: qualitative, quantitative and mixed methods*. Sage.

34. Creswell, JW. (2009). *Research Design: Qualitative, Quantitative and Mixed Methods Approaches*. Sage.

35. Greene, M. (2014). On the inside looking in: Methodological insights and challenges in conducting qualitative insider research. *The Qualitative Report*, 19 (15), 1-13.

For peer review only

Supplementary File 1 – Information Email #1: Introductory information about the study (including the overall Participant Information Sheet) and invitation to attend information webinar #1

To be sent by the INFOSAN Secretariat (infosan@who.int) on behalf of the researcher (cc: c.savelli@lancaster.ac.uk)

Health & Medicine | Lancaster University

Information Email #1

A mixed-methods exploration into the experience of members of the International Food Safety Authorities Network (INFOSAN): Introductory Information

---

Dear INFOSAN Member,

My name is Carmen Savelli and I am conducting this study as a student in the PhD Public Health programme at Lancaster University, Lancaster, United Kingdom. You may also know me as one of the Technical Officers working as the INFOSAN Secretariat at the World Health Organization (WHO) in Geneva, Switzerland.

What is the study about?
The purpose of this study is to interrogate INFOSAN in order to describe and explore the experiences of members and better understand the role of the network in mitigating the burden of foodborne illness around the world. The study will examine access to and usage of the INFOSAN Community Website, explore barriers and facilitators to active participation in INFOSAN, determine perceptions about the utility of INFOSAN to mitigate foodborne illness, and scrutinize if and how participation in this network creates value for members.

Why have I been approached?
You have been approached because the study requires information from registered INFOSAN members.

Do I have to take part?
No. It is completely up to you to decide whether or not you take part. The study is designed in three phases: Phase 1 will examine access and usage patterns of the INFOSAN Community Website; Phase 2 will involve the completion of an online survey; and Phase 3 will involve individual interviews conducted online using WebEx. Taking part in one phase of the study does not require your participation in the other phases.

What will I be asked to do if I take part?
Participation in Phase 1 of the study requires no further action on your part. Data on access and usage of the INFOSAN Community Website has already been collected as part of the ongoing monitoring and evaluation activities of the INFOSAN Secretariat in accordance with the Terms and Conditions of Use of the INFOSAN Community Website. **Any members not wishing to have their website access and usage data utilized for the purpose of this particular study should make this known by email to the researcher within the next two weeks (c.savelli@lancaster.ac.uk).** Those who are recruited in Phase 1 will have a further two weeks to opt out of the study, after which time this will no longer be possible due to aggregation of data.

Participation in Phase 2 of the study requires the completion of an online survey of approximately 30-45 minutes which will explore questions about your experience as an INFOSAN member. Once the survey has been submitted, it will not be possible to opt out because survey responses are anonymous.

Participation in Phase 3 of the study will involve a one-on-one individual interview conducted online using WebEx with the researcher. The interview will last approximately 45-60 minutes and will explore in more detail your experiences as an INFOSAN Member. Those who are recruited for Phase 3 of the study will have two weeks to opt out after which time this will no longer be possible.

Continued on next page…

Continued from previous page…

Will my data be Identifiable?

No. The information you provide will be reported anonymously. Any direct quotes from participants in Phase 3 will be reported anonymously. Quantitative data will be reported by region so that no country-specific details will be reported. The data collected for this study will be stored securely and only the researchers conducting this study will have access to this data:

- Audio recordings will be deleted once the thesis has been examined and accepted
- The files on the computer will be encrypted (that is no-one other than the researcher will be able to access them) and the computer itself password protected for the duration of the study
- At the end of the study, all related electronic information will be archived for 10 years in secure encrypted storage on the Lancaster University server. At the end of this period, they will be destroyed.
- The typed version of your interview will be made anonymous by removing any identifying information including your name. Anonymised direct quotations from your interview may be used in the reports or publications from the study, and neither your name nor your country will be attached to them.
- All your personal data will be confidential and will be kept separately from your interview responses (if you participate in Phase 3). However, there are some limits to confidentiality in Phase 3: if what is said in the interview makes me think that you, or someone else, is at significant risk of harm, I will have to break confidentiality and speak to a member of staff about this. If possible, I will tell you if I have to do this. In addition, whilst every effort will be made, it is not possible for the researcher to ensure confidentiality of your participation in Phase 3 if you choose to connect to the researcher by WebEx on your work premises during the working day. If you volunteer to participate in Phase 3, you are encouraged to connect by WebEx in a private room to conduct your interview, where no one else can see your computer screen or hear your conversation.

For further information about how Lancaster University processes personal data for research purposes and your data rights please visit our webpage: www.lancaster.ac.uk/research/data-protection

What will happen to the results?

The results will be summarised and reported in a PhD thesis and may be submitted for publication in relevant academic or professional conferences and journals or other media including books or websites, pending necessary clearance from WHO. Only those data collected with the expressed consent of participants is utilized and reported on for the purposes of this study. INFOSAN Members will also be invited to participate in webinars after each phase to learn about the results and next steps.

Are there any risks?

There are no risks anticipated with participating in this study. However, if you experience any distress following participation you are encouraged to inform the researcher and contact the resources provided at the end of this sheet. Neither participating nor abstaining will impact your future treatment as an INFOSAN member or the technical support provided to you or your country by the WHO.

Are there any benefits to taking part?

Although you may find participating interesting, there are no direct benefits in taking part. However, some participants may find it a positive experience to reflect on their role as an INFOSAN member as well as their contributions to a safer food supply, and to participate in research which they may feel is of relevance to them, their institution, their country or the global community.

Who has reviewed the project?

This study has been reviewed and approved by the Ethics Review Committee at the World Health Organization and the Faculty of Health and Medicine Research Ethics Committee at Lancaster University.

Where can I obtain further information about the study if I need it?

If you have any questions about the study, please contact the researcher:

Carmen Savelli
c.savelli@lancaster.ac.uk
+41 22 791 3234

Or his primary supervisor in the Department of Health Research at Lancaster University:

Dr Céu Mateus
c.mateus@lancaster.ac.uk
+44 1524 593182

Continued on next page…

Continued from previous page…

Or attend the upcoming webinar:

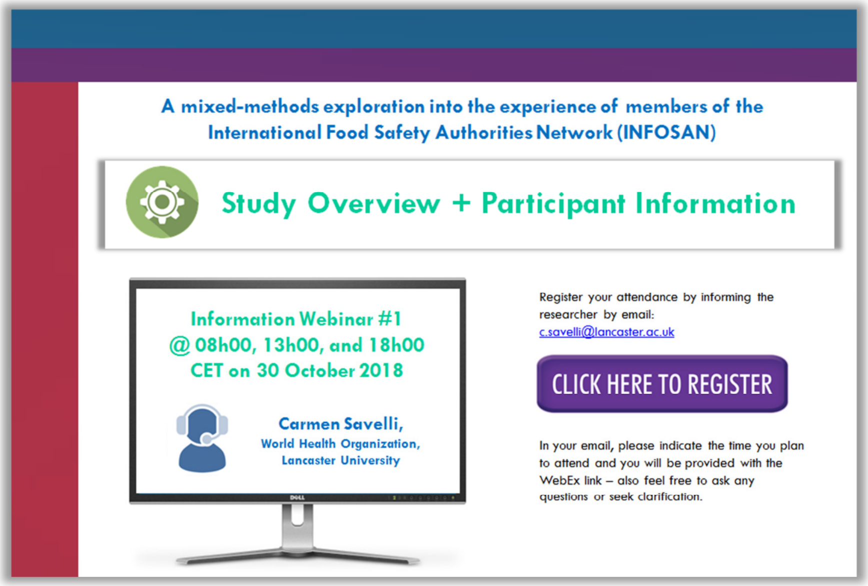

Complaints
If you wish to make a complaint or raise concerns about any aspect of this study and do not want to speak to the researcher or his supervisor, you can contact:

Professor Catherine Walshe
Head of the Division of Health Research
+44 (0)1524 510124
c.walshe@lancaster.ac.uk
Lancaster University
Lancaster
LA1 4YW

If you wish to speak to someone outside of the Public Health Doctorate Programme, you may also contact:

Professor Roger Pickup Tel: +44 (0)1524 593746
Associate Dean for Research Email: r.pickup@lancaster.ac.uk
Faculty of Health and Medicine
(Division of Biomedical and Life Sciences)
Lancaster University
Lancaster
LA1 4YG

Thank you for taking the time to read this information.

Resources in the event of distress
Should you feel distressed either as a result of taking part, or in the future, the following resource may be of assistance:
https://online.supportgroups.com/

Kind regards,

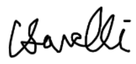

Carmen Savelli

Supplementary File 2 – Terms and Conditions of Use of the INFOSAN Community Website

The following disclaimer text is displayed on the online registration form of the ICW (https://extranet.who.int/infosan/registration) and must be accepted by all INFOSAN members to in order to complete registration.

Please take note of the following terms of use and disclaimers. By accessing the International Food Safety Authorities Network (INFOSAN) Community Website, the user agrees to accept these terms of use and disclaimers. The Food and Agriculture Organization of the United Nations (FAO) and the World Health Organization (WHO) reserve the right to update these terms of use and disclaimers without notice.

The INFOSAN Community Website is intended for the use of members of INFOSAN for the purpose of managing information about food safety risks in an interactive and collaborative manner. The content within the INFOSAN Community Website is intended for the internal use of registered INFOSAN members only and may not be disclosed, shared, distributed or transmitted to persons outside the Network, or duplicated, republished, reused for presentation or used in any other way, including for advertising or commercial purposes, without the prior written consent from FAO and WHO.

Although all reasonable precautions have been taken to verify the content contained on the INFOSAN Community Website, FAO and WHO make no warranties or representations regarding the content, presentation, appearance, completeness or accuracy and shall not be held liable for any damages whatsoever as a result of its use or application. The responsibility for the interpretation and use of the content lies with the reader and/or user. FAO and WHO reserve the right to make updates and changes without notice and accept no liability for any errors or omissions in this regard.

FAO and WHO accept no responsibility whatsoever for any inaccurate advice or information that is provided by sources reached via links or references to the content of the INFOSAN Community Website.

The INFOSAN Community Website may contain links to other Internet pages. With regard to these third-party websites, FAO and WHO have no control whatsoever over the content of the linked pages. FAO and WHO therefore shall not be held responsible whatsoever for the content of a page to be reached via such a link and shall not be held liable for any damages arising from their use. FAO and WHO reserve the right to make changes or additions, without prior notification, to the links provided.

The views expressed on the INFOSAN Community Website are those of the authors and do not necessarily reflect those of FAO and WHO.

The designations employed and the presentation of content on the INFOSAN Community Website, including maps and other illustrative materials, do not imply the expression of any opinion whatsoever on the part of FAO or WHO concerning the legal status of any country, territory, city or area, or of its authorities, or concerning the delineation of frontiers and borders.

The mention of specific companies or of certain manufacturers' products does not imply that they are endorsed or recommended by FAO or WHO in preference to others of a similar nature that are not mentioned.

Utilization of website analytics tools or other methods will be applied periodically to summarize members' access to and usage of the INFOSAN Community Website for monitoring, evaluation and research purposes.

[ ] Accept Terms & Conditions of Use

For peer review only

Supplementary File 3 – Information Email #2: Results from Phase 1 and invitation to attend Webinar #2 (includes Phase 2 Participant Information)

To be sent by the INFOSAN Secretariat (infosan@who.int) on behalf of the researcher (cc: c.savelli@lancaster.ac.uk)

Health & Medicine | Lancaster University

Information Email #2

A mixed-methods exploration into the experience of members of the International Food Safety Authorities Network (INFOSAN): Phase 1 results and information about Phase 2

Dear INFOSAN Member,

My name is Carmen Savelli and in August 2018 I contacted you to let you know that I was conducting this study as a student in the PhD Public Health programme at Lancaster University, Lancaster, United Kingdom. You may also know me as one of the Technical Officers working as the INFOSAN Secretariat at the World Health Organization (WHO) in Geneva, Switzerland. I am writing again to inform you of the progress made so far, including results from Phase 1 and provide some additional information for participants in Phase 2.

What is the overall study about?

The purpose of this study is to interrogate INFOSAN in order to describe and explore the experiences of members and better understand the role of the network in mitigating the burden of foodborne illness around the world. The study will examine access to and usage of the INFOSAN Community Website, explore barriers and facilitators to active participation in INFOSAN, determine perceptions about the utility of INFOSAN to mitigate foodborne illness, and scrutinize if and how participation in this network creates value for members.

The study is designed in three phases: Phase 1 is complete and has examined access and usage patterns of the INFOSAN Community Website; Phase 2 will involve the completion of an online survey; and Phase 3 will involve individual interviews conducted online using WebEx. Taking part in one phase of the study does not require your participation in the other phases.

What are the results from Phase 1?

To be inserted

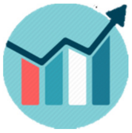

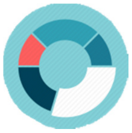

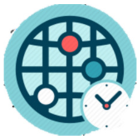

Why have I been approached about Phase 2 of this study?

You have been approached because the study requires information from registered INFOSAN members.

Do I have to take part in Phase 2?

No. It is completely up to you to decide whether or not you take part. Once the survey has been submitted, it will not be possible to opt out because survey responses are anonymous.

Continued on next page…

Continued from previous page…

What will I be asked to do if I take part?

Participation in Phase 2 of the study requires the completion of an online survey of approximately 30-45 minutes which will explore questions about your experience as an INFOSAN member. All INFOSAN Members will receive a separate invitation by email(Information Email #3) to provide consent and take the survey.

Will my data be Identifiable?

No. The information you provide will be reported anonymously. Quantitative data will be reported by region so that no country-specific details will be reported. The data collected for this study will be stored securely and only the researchers conducting this study will have access to this data:

- The files on the computer will be encrypted (that is no-one other than the researcher will be able to access them) and the computer itself password protected for the duration of the study
- At the end of the study, all related electronic information will be archived for 10 years in secure encrypted storage on the Lancaster University server. At the end of this period, they will be destroyed.

For further information about how Lancaster University processes personal data for research purposes and your data rights please visit our webpage: www.lancaster.ac.uk/research/data-protection

What will happen to the results?

The results will be summarised and reported in a PhD thesis and may be submitted for publication in relevant academic or professional conferences and journals or other media including books or websites, pending necessary clearance from WHO. Only those data collected with the expressed consent of participants is utilized and reported on for the purposes of this study. INFOSAN Members will also be invited to participate in a webinar after Phase 2 to learn about the results and next steps.

Are there any risks?

There are no risks anticipated with participating in this study. However, if you experience any distress following participation you are encouraged to inform the researcher and contact the resources provided at the end of this sheet. Neither participating nor abstaining will impact your future treatment as an INFOSAN member or the technical support provided to you or your country by the WHO.

Are there any benefits to taking part?

Although you may find participating interesting, there are no direct benefits in taking part. However, some participants may find it a positive experience to reflect on their role as an INFOSAN member as well as their contributions to a safer food supply, and to participate in research which they may feel is of relevance to them, their institution, their country or the global community.

Who has reviewed the project?

This study has been reviewed and approved by the Ethics Review Committee at the World Health Organization and the Faculty of Health and Medicine Research Ethics Committee at Lancaster University.

Where can I obtain further information about the study if I need it?

If you have any questions about the study, please contact the researcher:

Carmen Savelli
c.savelli@lancaster.ac.uk
+41 22 791 3234

Or his primary supervisor in the Department of Health Research at Lancaster University:

Dr Céu Mateus
c.mateus@lancaster.ac.uk
+44 1524 593182

Or attend the upcoming webinar:

Continued on next page…

Continued from previous page…

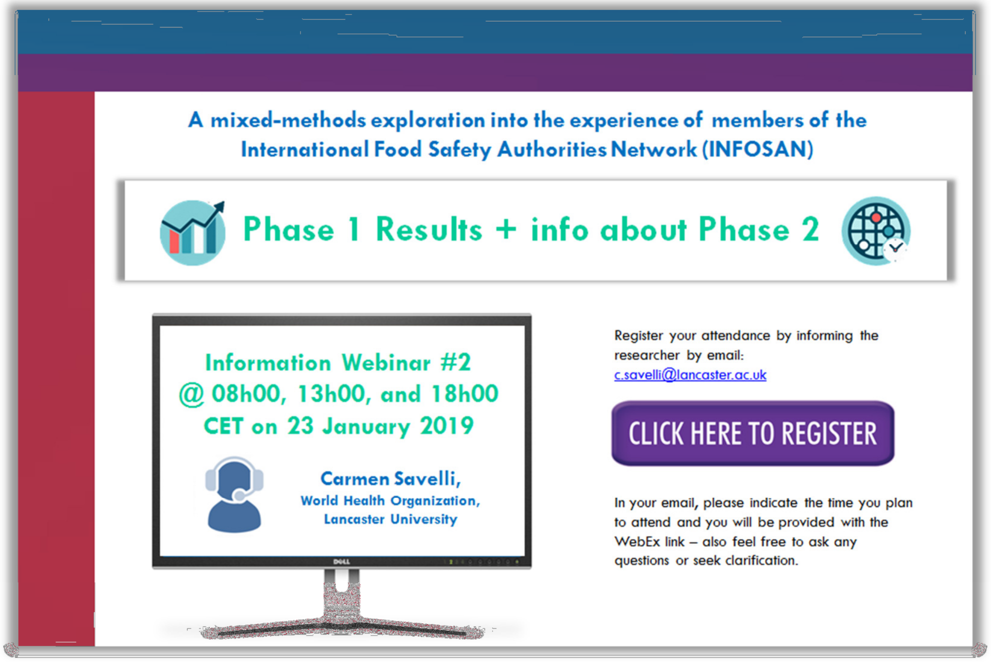

Complaints
If you wish to make a complaint or raise concerns about any aspect of this study and do not want to speak to the researcher or his supervisor, you can contact:

Professor Catherine Walshe
Head of the Division of Health Research
+44 (0)1524 510124
c.walshe@lancaster.ac.uk
Lancaster University
Lancaster
LA1 4YW

If you wish to speak to someone outside of the Public Health Doctorate Programme, you may also contact:

Professor Roger Pickup Tel: +44 (0)1524 593746
Associate Dean for Research Email: r.pickup@lancaster.ac.uk
Faculty of Health and Medicine
(Division of Biomedical and Life Sciences)
Lancaster University
Lancaster
LA1 4YG

Thank you for taking the time to read this information.

Resources in the event of distress
Should you feel distressed either as a result of taking part, or in the future, the following resource may be of assistance:
https://online.supportgroups.com/

Kind regards,

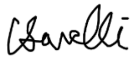

Carmen Savelli

Supplementary File 4 – Information Email #3: Invitation to participate in Phase 2 including consent information and link to online survey

To be sent by the INFOSAN Secretariat (infosan@who.int) on behalf of the researcher (cc: c.savelli@lancaster.ac.uk)

Health & Medicine | Lancaster University

Information Email #3

A mixed-methods exploration into the experience of members of the International Food Safety Authorities Network (INFOSAN): Invitation to participate in Phase 2

Dear INFOSAN member,

You are invited to participate in Phase 2 of our study to explore the experiences of members of the International Food Safety Authorities Network (INFOSAN).

Phase 2 of the study involves completing an online survey that should take approximately 30-45 minutes of your time. Complete background details are available in the Participant Information Sheet, shared with you previously and also attached for ease of reference.

By proceeding to the survey you confirm that:

- ✓ You have read the information sheet and understand what is expected of you within this study;
- ✓ You confirm that you understand that any responses/information you give will remain anonymous;
- ✓ Your participation is voluntary;
- ✓ You consent for the information you provide to be discussed with my supervisor at Lancaster University;
- ✓ You consent to Lancaster University keeping the anonymised data for a period of 10 years after the study has finished;
- ✓ By clicking on the button below, you consent to taking part in the current study.

If you have any questions about this survey or other aspects of the study, please do not hesitate to contact me at any time: c.savelli@lancaster.ac.uk

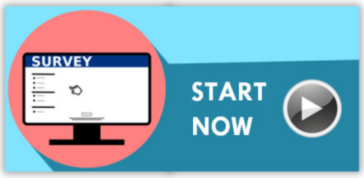

Kind regards,

Carmen Savelli

Supplementary File 5 – Draft questionnaire for Phase 2

Most of the following questions have been taken directly from the Community Assessment Tool (Verburg and Andriessen, 2006). Those questions in written in blue text are supplementary questions, added to the questionnaire in order to address INFOSAN-specific items. In addition, the likert-type scale response anchors have been added as per Vagias and Wade (2006).

PERSONAL DATA

Age ___________

Gender ___________

Country ___________

**A) INTRODUCTION**
Are you a member of more than one food-safety related community of practice?<br><br>If yes, which one(s): ____

**How long (approximately) have you been a member of INFOSAN?**					
	Weeks	Months	Years	Don't know	Prefer not to answer
Specify number in corresponding box	#	#	#		

	Focal Point	Emergency Contact Point	Don't know	Prefer not to answer
**What type of member are you?**				

**B) GOALS**
**How important are the following goals for you personally as a member of this community? That means, for you personally, what are the most important reasons for participating in INFOSAN?**

For peer review only

	Not Important At All	Of Little Importance	Of Average Importance	Very Important	Absolutely Essential	Don't know	Prefer not to answer
– Hearing about new knowledge and experiences from other community members							
– Developing together new ideas for the community							
– Having nice meetings, fun and non-work related activities							
– Developing standards, methods and best practices							
– Making useful contacts/ networking							
– Improving the level of expertise of other members							
– Staying up to date in the topic of the community (i.e. food safety issues)							
– Saving time in finding all kinds of information							
– To advance in my career							
– Helping newcomers in the community							

C) ACTIVITIES

How often do the following activities happen in your community?

	Never	Almost Never	Occasionally	Frequently	Very Frequently	Don't know	Prefer not to answer
– Meetings with discussions							
– Talks about experiences							
– Presentations by members							
– Presentations by non-members							
– Workshops							
– Team-building activities							
– Members writing reports or other publications together							
– Brainstorming							
– Writing project proposals							
– Exchanging e-mails to find solutions to problems							

To what extent would you prefer a change in these activities?

	Decrease frequency greatly	Decrease frequency slightly	No change	Increase Frequency slightly	Increase frequency greatly	Don't know	Prefer not to answer

For peer review only

– Meetings with discussions							
– Talks about experiences							
– Presentations by members							
– Presentations by non-members							
– Workshops							
– Team building activities (such as informal outings)							
– Brainstorming							
– Writing project proposals							
– Members writing publications together							
– Exchanging e-mails to find solutions to problems							

	Very poor	Poor	Acceptable	Good	Very Good	Don't know	Prefer not to answer
**How would you rate the way people interact in this community?**							

Does your community have the following types of meetings?

	Never	Almost Never	Occasionally	Frequently	Very Frequently	Don't know	Prefer not to answer
– Face to face meetings							
– Virtual meetings (e.g. video conferences, telephone conferences, via the internet)							

D) PARTICIPATION

	Not at all	Almost never	Occasionally	Very	Extremely	Don't know	Prefer not to answer
**How actively do you participate in the community?**							

For peer review only

How often do you have the following types of contact with other members of your community outside of community meetings?							
	Never	Almost Never	Occasionally	Frequently	Very Frequently	Don't know	Prefer not to answer
– Email exchanges							
– Phone calls							
– Face to face meetings							
– Virtual meetings (e.g. video conferences, telephone conferences, via the internet)							
– Instant chats on the INFOSAN Community Website							
– Discussion forum on the INFOSAN Community Website							

On average, how many hours do you spend on the community per month? _________

How many members do you know by face? ______________

With how many members have you had personal contact? ________________

INFOSAN has an important mandate, to communicate between national government authorities around the world to keep the global food supply safe. However, some members are more active than others. The following section pertains to potential barriers to participation in INFOSAN.

To what extent do you agree that the following factors limit your participation in INFOSAN Activities?							
	Strongly Disagree	Disagree	Undecided	Agree	Strongly Agree	Don't know	Prefer not to answer
My role as an INFOSAN member is unclear to me							
My country has limited capacity and/or infrastructure dedicated to addressing food safety events							
My country has insufficient funds dedicated to monitoring and/or responding to food safety events.							
My country lacks human resources and/or expertise dedicated to addressing food safety events.							
My country's national food control system is either underdeveloped or non-existent							

For peer review only

My country's national-level Food Safety Emergency Response Plan is either underdeveloped or non-existent							
There are difficulties in identifying contaminated food and/or ingredients within my country							
There are challenges in conducting food safety risk assessments within my country							
Personnel in my country are not trained to respond to food safety events							
There is a need for a simpler and more standardized way to share information between national authorities within my country							
There is a lack of coordination and/or communication between national authorities within my country							
Legal implications at the national level hinder prompt information-sharing with the INFOSAN Secretariat and INFOSAN member countries							
12. There are political constraints at the national level (e.g. the government does not prioritize food safety, the Emergency Contact Point must reflect national views when communicating with the INFOSAN Secretariat, etc.)							
There are concerns in my country that reporting a food safety event with potential international implications might negatively impact the country's economy or competitive interests (e.g. trade, tourism, etc.)							
There are language barriers.							
There is a lack of trust between INFOSAN members							
There are fears that the confidential nature of information shared may not be respected by parties involved (e.g. the information may not be safeguarded, may be disseminated inappropriately, etc.)							

E) COORDINATION

How active is the co-ordinator/ facilitator of your community (i.e. the INFOSAN Secretariat) in the following activities?

	Never	Almost Never	Occasionally	Frequently	Very Frequently	Don't know	Prefer not to answer
– Organising meetings							
– Stimulating members to participate in the community							
– Sharing his/ her own expertise with the community members							
– Co-ordinating activities on the INFOSAN Community Website							
– Making external contacts							
– Connecting the community members with each other							

For peer review only

– Promoting the community publicly							
– Alerting members to interesting external activities (e.g. conferences)							

To what extent do you agree that the following statements about the INFOSAN Secretariat?

	Strongly Disagree	Disagree	Neither Agree nor disagree	Agree	Strongly Agree	Don't know	Prefer not to answer
I fully understand the role of the INFOSAN Secretariat.							
Information communicated by the INFOSAN Secretariat is always sufficiently prompt.							
Information communicated by the INFOSAN Secretariat is always accurate.							

	Very dissatisfied	Dissatisfied	Neutral	Satisfied	Very Satisfied	Don't know	Prefer not to answer
**How satisfied are you with the co-ordination of INFOSAN?**							

F) INFORMATION AND COMMUNICATION TECHNOLOGY SUPPORT

How useful do you think the following means of communication are (or might be) particularly for the work of INFOSAN?

	Not useful at all	Not very useful	No opinion	Very Useful	Essential	Don't know	Prefer not to answer
– Scheduled face to face meetings							
– Informal face to face encounters							
– E-mail							
– Telephone conferences							
– Fax							
– Video connection							
– Chat/ shared white board/ application sharing system							
– Special discussion list / newsgroup for the community							

For peer review only

– Written memos or reports							
– Group calendar/ shared project planning tool							
– Document sharing tool							
– Groupware to work jointly on documents (co-editing)							
– A database/ knowledge system in which you are expected to fill in your project experiences and relevant knowledge							

The INFOSAN Community Website was launched in 2012 and all members of INFOSAN are registered on there. This online portal is the gateway through which all members can communicate with each other and with the INFOSAN Secretariat.

**Regarding the INFOSAN Community Website:**							
	Never	Every few months	Monthly	Weekly	Daily	Don't know	Prefer not to answer
How often do you access the INFOSAN Community Website?							

**What do you normally use the INFOSAN Community Website for? (check all that apply)**	
	To read about Alerts issued by the INFOSAN Secretariat
	To read INFOSAN Documents
	To read publications and newsletters from FAO/WHO
	To participate in the Discussion Forum
	To find other INFOSAN members' contact details
	To discuss with other INFOSAN members using the "Chat" function on the INFOSAN Community Website
	To participate in or manage Groups
	Other; Please specify
	Don't know
	Prefer not to answer

**To what extent do you agree that the following statements about the INFOSAN Community Website?**							
	Strongly Disagree	Disagree	Neither Agree nor disagree	Agree	Strongly Agree	Don't know	Prefer not to answer
The INFOSAN Community Website an important and supportive tool for the network							
The volume of information posted on the INFOSAN							

For peer review only

Community Website by the **Secretariat** is sufficient							
The volume of information posted on the INFOSAN Community Website by the **members** is sufficient.							
A mobile friendly version of the INFOSAN Community Website would improve participation							
The INFOSAN Community Website provides a single integrated point of access to a variety of relevant food safety emergency information for INFOSAN members around the world.							
The INFOSAN Community Website creates and maintains knowledge directories about portal generated content including details about food safety events and emergencies.							
The INFOSAN Community Website facilitates information sharing and provides collaborative features that help to foster the Community of Practice among INFOSAN members							
The INFOSAN Community Website facilitates information sharing and provides collaborative features that help to foster the Community of Practice among INFOSAN members							

**G) ORGANIZATIONAL SUPPORT**							
	Never	Almost Never	Occasionally	Frequently	Very Frequently	Don't know	Prefer not to answer
Does your organisation allocate time for you to participate in INFOSAN?							
	Strongly discouraged	Discouraged	Neither discouraged or encouraged	Encouraged	Strongly Encouraged	Don't know	Prefer not to answer
All in all how encouraged do you feel by your organisation to participate actively in INFOSAN?							

	Not at all	To a small extent	To a moderate extent	To a great extent	To a very great extent	Don't know	Prefer not to answer

For peer review only

Would you like to have more time available for activities concerning INFOSAN?							

H) VALUE OF INFOSAN AS A COMMUNITY OF PRACTICE

How important are the following ways for you to find new information (e.g. for solving problems, for doing projects, for keeping up to date in your field)?

	Not important at all	Not very important	Important	Very important	Extremely important	Don't know	Prefer not to answer
– Through INFOSAN							
– Through individual colleagues							
– Via the internet							
– Asking an expert							
– Reading publications or reports							
– Through database consulting							
– Other	explain						

To what extent would you personally like INFOSAN to continue?

	Not at all	To a small extent	To a moderate extent	To a great extent	To a very great extent	Don't know	Prefer not to answer

Please indicate the extent to which you agree with the following statements.

	Strongly Disagree	Disagree	Neither Agree nor disagree	Agree	Strongly Agree	Don't know	Prefer not to answer

– Some knowledge is so valuable to me that I would not easily share it with others							
– The most important thing that happens in our community is that members find solutions to problems in their work							
– Knowledge is now so developed in the community that it can be turned into innovative ideas							
– Thanks to the combination of the different skills and views in our community many new ideas have been developed							
– People in the community are reluctant to talk about things that have gone wrong in their projects/ work							
– Newcomers have much to learn before they are at the same level as the existing members							
– It is sometimes difficult to learn from each other in the community because we have very different backgrounds							
– Knowledge is now so developed in the community that it can be written up for e.g. manuals, training courses, best practices							
– Most members in this community have developed a strong common understanding and identity over time							

How often does it happen that:

	Never	Almost Never	Occasionally	Frequently	Very Frequently	Don't know	Prefer not to answer
You have some knowledge or experience that you prefer NOT to share with members of the community							
You hear experiences and other things that you already know							

INFOSAN has been in operation since 2004, and this study is the first of its kind to try and determine if the network has value to members in a robust and rigorous way.

Please indicate the extent to which you agree with the following statements.

	Strongly Disagree	Disagree	Neither Agree no disagree	Agree	Strongly Agree	Don't know	Prefer not to answer
Participating in INFOSAN has been a valuable							

For peer review only

experience for me							
Participating in INFOSAN has connected me with other food safety professionals around the world.							
Participating in INFOSAN has prevented foodborne illnesses in other countries							
Participating in INFOSAN makes me feel a part of a global community of practice of food safety professions.							

Participating in INFOSAN has provided me with information that was used in my country to **implement risk management measures**.

[ ] Yes [ ] No [ ] Don't know

If Yes, during how many different food safety events? ☐1 ☐2-5 ☐ 6-10 ☐11-20 ☐ >20

If Yes, can you name an example? ____

Participating in INFOSAN has **prevented foodborne illnesses** in my country.

[ ] Yes [ ] No [ ] Don't know

If Yes, during how many different food safety events? ☐1 ☐2-5 ☐ 6-10 ☐11-20 ☐ >20

If Yes, can you name an example? ____

**I) RESULTS**							
**To what extent do you think the members of INFOSAN . . .**							
	Not at all	Almost never	Somewhat	Mostly	Completely	Don't know	Prefer not to answer
– Trust each other							
– Feel a sense of loyalty to INFOSAN							
– Have a good common understanding							
– Feel a sense of belonging to INFOSAN							

**To what extent do you think INFOSAN has. . .**

For peer review only

	Not at all	Almost never	Seldom	Frequently	Constantly	Don't know	Prefer not to answer
– Contributed to cost savings for your organisation							
– Contributed new ideas to your organisation<br>– Made a real contribution to the effectiveness of your organisation							
– Contributed to developing new methods or approaches for your organisation							
– Contributed to the documentation of information, such as knowledge systems, manuals, training instructions or best practices							
– A good reputation in your organisation							

**To what extent do you think the members are . . .**							
	Never	Rarely	Sometimes	Often	Always	Don't know	Prefer not to answer
– Reluctant to share their knowledge with other members							
– Generally very enthusiastic and motivated to participate							

**Through being a member of INFOSAN, to what extent…**							
	Not at all	Slightly	Somewhat	Moderately	Extremely	Don't know	Prefer not to answer
– Have you learned a lot about your subject area?							
– Have you made useful new contacts?							
– Have you been able to solve problems in your work?							
– Have your career prospects improved?							
– Have you been better able to find all kinds of information?							
– Have you been able to work more efficiently?							
– Have you been better able to keep up to date in your field?							
– Has your reputation and visibility in the organisation improved?							

– Have you been able to transfer what you heard in the community to your project team(s)or department?							

All in all do you like being part of the community?

Yes [ ] **No [ ]** **Don't know [ ]** **Prefer not to answer [ ]**

**In your opinion, in what areas does the community need improvement? (check all that apply)**	
	– In the area of the tools that support community activities
	– The overall organization of the community
	– The way INFOSAN is supported by FAO/WHO
	– The co-ordination of INFOSAN
	– Other; Please specify:
	– Don't know
	– Prefer not to answer

Preface: INFOSAN aims to prevent the international spread of contaminated food and foodborne disease and strengthen food safety systems globally. The main objectives are to: 1) Promote the rapid exchange of information during food safety incidents/emergencies; 2) Share information on important food safety issues of global interest; 3) Promote partnership and collaboration between countries; and 4) Help countries strengthen their capacity to manage food safety emergencies.

Please indicate the extent to which you agree with the following statements.

	Strongly Disagree	Disagree	Neither Agree nor disagree	Agree	Strongly Agree	Don't know	Prefer not to answer
The objectives of INFOSAN are still valid							
The activities and outputs of INFOSAN are consistent with the overall goal and the attainment of its objectives							
The activities and outputs of the program are consistent with the intended impacts and effects							
The objectives of INFOSAN have been achieved							
INFOSAN activities are cost-efficient							
INFOSAN objectives are achieved in a timely manner							
INFOSAN activities are implemented in the most efficient way compared to alternatives							
Because of INFOSAN, illnesses have been prevented.							
Because of INFOSAN, lives have been saved.							
INFOSAN operates in a sustainable way.							

For peer review only

INFOSAN promotes the rapid exchange of information during food safety incidents/emergencies							
INFOSAN shares information on important food safety issues of global interest							
INFOSAN promotes partnership and collaboration between countries and networks							
INFOSAN helps countries strengthen their capacity to manage food safety emergencies							
INFOSAN has improved the safety of the global food supply.							
INFOSAN has reduced foodborne illness globally							
Information communicated by INFOSAN **members** is always sufficiently prompt							
Information communicated by INFOSAN **members** is always accurate							

For peer review only

Supplementary File 6 – Expansion and Adaptation of Questionnaire

Contributor	Step
Verburg and Andriessen (2006)	1. Questionnaire in English from the Community Assessment Toolkit (CAT)
INFOSAN Advisory Group (Appendix 8)	2. Supplemental questions reviewed for content validity by a panel of experts consisting of Members of the INFOSAN Advisory Group
C. Savelli, Technical Officer, INFOSAN Secretariat, Department of Food Safety and Zoonoses (FOS)	3. Supplemental questions revised based on feedback prior to pilot testing
Regional Food Safety Officers and Advisors from FAO and WHO (Appendix 9)	4. Regional Food Safety Officers and Advisors from FAO and WHO pilot test the supplemental questions
C. Savelli	5. Revision prior to finalization in English
French translation: RD, WHO Intern, FOS<br>Spanish translation: RG, WHO Consultant, FOS	6. Forward translations of CAT questionnaire and supplemental questions into French and Spanish
**French Panel:**<br>▪ RC - PhD Candidate, formerly INFOSAN Secretariat at WHO<br>▪ CM – MD - WHO Implementation Research Scientist<br>▪ PBE - PhD, WHO, INFOSAN Secretariat<br>▪ RD - WHO Intern, FOS<br>**Spanish Panel**:<br>▪ EP – PhD, DVM, WHO Regional Food Safety Advisor<br>▪ SR – DVM, WHO Regional Food Safety Advisor<br>▪ JM – WHO, Technical Officer, FOS<br>▪ RG, WHO Consultant, FOS	7. Review of entire questionnaire by expert panels
C. Savelli	8. Revision as required
WHO Translation services	9. Backward translations into English
C. Savelli	10. Revision as required
WHO Interns	11. Pilot test
C. Savelli	12. Revision as required to finalized version

Supplementary File 7 – Information Email #4: Results from Phase 2 and invitation to attend Webinar #3 (includes Phase 3 Participant Information)

To be sent by the INFOSAN Secretariat (infosan@who.int) on behalf of the researcher (cc: c.savelli@lancaster.ac.uk)

Health & Medicine | Lancaster University

Information Email #4

A mixed-methods exploration into the experience of members of the International Food Safety Authorities Network (INFOSAN): Phase 2 results and information about Phase 3

Dear INFOSAN Member,

My name is Carmen Savelli and in August 2018 I first contacted you to let you know that I was conducting this study as a student in the PhD Public Health programme at Lancaster University, Lancaster, United Kingdom. You may also know me as one of the Technical Officers working as the INFOSAN Secretariat at the World Health Organization (WHO) in Geneva, Switzerland. I am writing again to inform you of the progress made so far, including results from Phase 2 and provide some additional information for participants in Phase 3.

What is the overall study about?

The purpose of this study is to interrogate INFOSAN in order to describe and explore the experiences of members and better understand the role of the network in mitigating the burden of foodborne illness around the world. The study will examine access to and usage of the INFOSAN Community Website, explore barriers and facilitators to active participation in INFOSAN, determine perceptions about the utility of INFOSAN to mitigate foodborne illness, and scrutinize if and how participation in this network creates value for members.

The study is designed in three phases: Phase 1 is complete and has examined access and usage patterns of the INFOSAN Community Website; Phase 2 is complete and involved the completion of an online survey by INFOSAN members; and Phase 3 will involve individual interviews conducted online using WebEx. Taking part in one phase of the study does not require your participation in the other phases.

What are the results from Phase2?

To be inserted

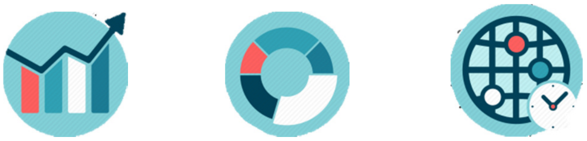

Why have I been approached about Phase 3 of this study?

You have been approached because the study requires information from registered INFOSAN members.

Do I have to take part in Phase 3?

No. It is completely up to you to decide whether or not you take part. Between 6 and 12 volunteers will be selected for Phase 3. If more than 2 people from a single WHO region indicate their interest in participating in Phase 3, the two people who have been members the longest will be selected and notified by email from the researcher, providing they are not from the same country.

Continued on next page…

Continued from previous page…

What will I be asked to do if I take part?

Participation in Phase 3 of the study will involve a one-on-one individual interview conducted online using WebEx with the researcher. The interview will last approximately 45-60 minutes and will explore in more detail your experiences as an INFOSAN Member.

To volunteer for Phase 3, please express your interest by contacting the researcher: c.savelli@lancaster.ac.uk

Will my data be Identifiable?

No. The information you provide will be reported anonymously. Any direct quotes from participants in Phase 3 will be reported anonymously. Quantitative data will be reported by region so that no country-specific details will be reported. The data collected for this study will be stored securely and only the researchers conducting this study will have access to this data:

- Audio recordings will be deleted once the thesis has been examined and accepted
- The files on the computer will be encrypted (that is no-one other than the researcher will be able to access them) and the computer itself password protected for the duration of the study
- At the end of the study, all related electronic information will be archived for 10 years in secure encrypted storage on the Lancaster University server. At the end of this period, they will be destroyed.
- The typed version of your interview will be made anonymous by removing any identifying information including your name. Anonymised direct quotations from your interview may be used in the reports or publications from the study, and neither your name nor your country will be attached to them.
- All your personal data will be confidential and will be kept separately from your interview responses. There are some limits to confidentiality: if what is said in the interview makes me think that you, or someone else, is at significant risk of harm, I will have to break confidentiality and speak to a member of staff about this. If possible, I will tell you if I have to do this. In addition, whilst every effort will be made, it is not possible for the researcher to ensure confidentiality of your participation in Phase 3 if you choose to connect to the researcher by WebEx on your work premises during the working day. If you volunteer to participate in Phase 3, you are encouraged to connect by WebEx in a private room to conduct your interview, where no one else can see your computer screen or hear your conversation.

For further information about how Lancaster University processes personal data for research purposes and your data rights please visit our webpage: www.lancaster.ac.uk/research/data-protection

What will happen to the results?

The results will be summarised and reported in a PhD thesis and may be submitted for publication in relevant academic or professional conferences and journals or other media including books or websites, pending necessary clearance from WHO. Only those data collected with the expressed consent of participants is utilized and reported on for the purposes of this study. INFOSAN Members will also be invited to participate in webinars after each phase to learn about the results and next steps.

Are there any risks?

There are no risks anticipated with participating in this study. However, if you experience any distress following participation you are encouraged to inform the researcher and contact the resources provided at the end of this sheet. Neither participating nor abstaining will impact your future treatment as an INFOSAN member or the technical support provided to you or your country by the WHO.

Are there any benefits to taking part?

Although you may find participating interesting, there are no direct benefits in taking part. However, some participants may find it a positive experience to reflect on their role as an INFOSAN member as well as their contributions to a safer food supply, and to participate in research which they may feel is of relevance to them, their institution, their country or the global community.

Who has reviewed the project?

This study has been reviewed and approved by the Ethics Review Committee at the World Health Organization and the Faculty of Health and Medicine Research Ethics Committee at Lancaster University.

Where can I obtain further information about the study if I need it?

If you have any questions about the study, please contact the researcher:

Carmen Savelli
c.savelli@lancaster.ac.uk
+41 22 791 3234

Or his supervisor in the Department of Health Research at Lancaster University:

Dr Céu Mateus
c.mateus@lancaster.ac.uk

Continued on next page…

Continued from previous page…

Or attend the upcoming webinar:

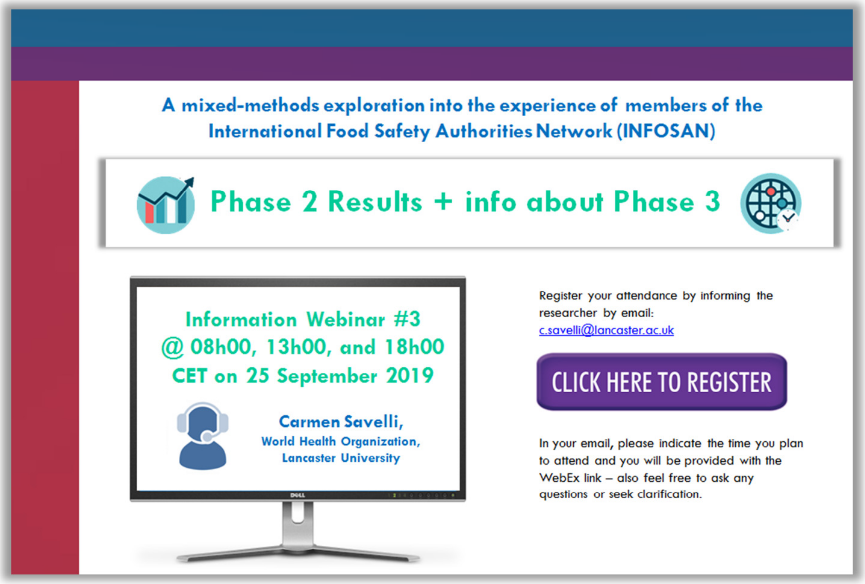

Complaints
If you wish to make a complaint or raise concerns about any aspect of this study and do not want to speak to the researcher or his supervisor, you can contact:

Professor Catherine Walshe
Head of the Division of Health Research
+44 (0)1524 510124
c.walshe@lancaster.ac.uk

Lancaster University
Lancaster
LA1 4YW

If you wish to speak to someone outside of the Public Health Doctorate Programme, you may also contact:

Professor Roger Pickup Tel: +44 (0)1524 593746
Associate Dean for Research Email: r.pickup@lancaster.ac.uk
Faculty of Health and Medicine
(Division of Biomedical and Life Sciences)
Lancaster University
Lancaster
LA1 4YG

Thank you for taking the time to read this information.

Resources in the event of distress
Should you feel distressed either as a result of taking part, or in the future, the following resource may be of assistance:
https://online.supportgroups.com/

Kind regards,

Carmen Savelli

Supplementary File 8 – Email to volunteers for Phase 3 that were not selected

To be sent from the researcher: c.savelli@lancaster.ac.uk

Health & Medicine | Lancaster University

Dear <<Name>>,

Many thanks for your interest in participating in Phase 3 of our study to explore the experiences of members of the International Food Safety Authorities Network (INFOSAN).

We have seen an overwhelmingly positive response to our call for volunteers to fill a limited number of interview slots. Unfortunately, due to resource restrictions including time restraints we will not be able to include you in this phase of the study.

Volunteers were selected on the basis of geographic location (maximum of two participants per WHO region) and length of membership to INFOSAN. If more than two members volunteered from the same region, the two who have been members of INFOSAN the longest were selected. This is what happened in your case.

Please be reminded that your active participation in the INFOSAN community is valued and appreciated and we hope you continue to engage in the important activities of the Network in the future.

If you have any additional information you wish to share regarding your experience as an INFOSAN member, you are always welcome to contact the INFOSAN Secretariat at WHO directly (infosan@who.int) outside of the context of this study.

Kind regards,

CSavelli

Carmen Savelli

Supplementary File 9 – Consent form for volunteers participating in Phase 3

To be sent from the researcher: c.savelli@lancaster.ac.uk

Health & Medicine | Lancaster University

A mixed-methods exploration into the experience of members of the International Food Safety Authorities Network (INFOSAN): Phase 3 consent form

Many thanks for your interest in participating in Phase 3 of our study to explore the experiences of members of the International Food Safety Authorities Network (INFOSAN).

Consent Form

Before you consent to participating in the study we ask that you re-read the participant information (contained in Information Email #4 and attached for ease of reference) and mark each box below with your initials if you agree. If you have any questions or queries before signing the consent form please contact the researcher, Carmen Savelli (c.savelli@lancaster.ac.uk or +41 (0) 79 9456 320).

Initial here

1. I confirm that I have read the participant information and fully understand what is expected of me within this study ☐
2. I confirm that I have had the opportunity to ask any questions and to have them answered. ☐
3. I understand that my interview will be audio recorded and then made into an anonymised written transcript. ☐
4. I understand that audio recordings will be kept until the research project has been examined. ☐
5. I understand that my participation is voluntary and that I am free to withdraw at any time without giving any reason. ☐
6. I understand that once my data have been anonymised and incorporated into themes it might not be possible for it to be withdrawn, though every attempt will be made to extract my data, up to the point of publication. ☐
7. I understand that the information from my interview will be pooled with other participants' responses, anonymised and may be published. ☐
8. I consent to information and quotations from my interview being used in reports, conferences and training events. ☐
9. I understand that the researcher will discuss data with their supervisor as needed. ☐
10. I understand that any information I give will remain confidential and anonymous unless it is thought that there is a risk of harm to myself or others, in which case the principal investigator will need to share this information with their research supervisor. ☐ ☐
11. I consent to Lancaster University keeping written transcriptions of the interview for 10 years after the study has finished. ☐
12. I consent to take part in the above study. ☐

Name of Participant__________________ Signature____________________ Date ___________

Name of Researcher __________________Signature ____________________Date ___________

Supplementary File 10 – Interview Guide for Phase 3

The semi-structured format of the interviews will be conversational in style, allowing the researcher and participant to engage in a dialogue where questions can be modified depending on responses. However, the following questions can serve to start discussions within the bounds of the four themes:

Theme 1) Use of the ICW to support network activities

Preface: The INFOSAN Community Website was launched in 2012 and all members of INFOSAN are registered on there. This online portal is the gateway through which all members can communicate with each other and with the INFOSAN Secretariat.

Questions about experiences:

- How often do you access the INFOSAN Community Website?
- What do you normally use it for?
- Do you think it is supporting the activities of INFOSAN?
- Do you think it could be improved and how?
- Do you think the INFOSAN Secretariat should be posting more frequently on the website?
- What kind of information do you think the INFOSAN Secretariat should be posting there?
- How do you think we might be able to get more members engaged on the website, e.g. involved in discussions, sharing documents, etc.?
- What new features would you like to see?
- Do you think a mobile version of the website would improve participation?

Theme 2) Barriers and facilitators to active participation in INFOSAN

Preface: INFOSAN has an important mandate, to communicate between national government authorities around the world to keep the global food supply safe. However, we know that some members are more active than others. Let's discuss some of the potential barriers to and facilitators to participation.

Questions about experiences:

- Is limited capacity in your country a barrier to participation (i.e. Limited capacity/infrastructure dedicated to addressing food safety; insufficient funds; human resources/expertise; national food control system underdeveloped) and if so, how?
- Is lack of training a barrier (Laboratory analysis; food safety risk assessment; outbreak investigation), and if so, how?
- Does a lack of national food safety standards pose a barrier, and if so, how?
- What is coordination between national authorities like at the national level?
- Does a lack of coordination pose a barrier, and if so, how?
- Are their legal constraints that bar your participation in INFOSAN (e.g. legal implications hinder prompt information sharing; lack of food safety legislation; lack of cooperation from industry, etc.), and if so, how?
- Is food safety a priority in your country? If not, does this pose a barrier, and if so, how?

For peer review only

- Are their perceptions that participating in INFOSAN may have a negative impact on the economy (i.e. through damages to trade or tourism sectors)?
- Is the mandate of INFOSAN clear? If not, how could it be clarified?
- If more members understood their roles and responsibilities better, would that facilitate participations? What would be the best way to do this?
- Have you ever attended any INFOSAN training either online or in person? What did you think about the training? How could the training be improved?
- Do you think language barriers restrict active participation in INFOSAN , and if yes, how so?
- Do you have concerns over confidentiality that limit what you share with INFOSAN?
- How could these concerns be addressed to help facilitate your improved participation?
- Are there any obvious weaknesses of INFOSAN, which if rectified could significantly improve the functioning and thereby the impact of INFOSAN?

Theme 3) Perceptions of network achievements and attainment of objectives (including reducing foodborne illness)

Preface: INFOSAN aims to prevent the international spread of contaminated food and foodborne disease and strengthen food safety systems globally, by: 1) Promoting the rapid exchange of information during food safety incidents/emergencies; 2) Sharing information on important food safety issues of global interest; 3) Promoting partnership and collaboration between countries; and 4) Helping countries strengthen their capacity to manage food safety risks "from farm to table".

Questions about experiences:

- How useful has INFOSAN been in promoting the rapid exchange of information during food safety incidents/emergencies?
- Have you been involved in such exchanges? What was that like? Did you get the information you needed quickly enough to respond and implement risk management measures?
- What have your experiences been like when dealing with the INFOSAN Secretariat?
- Does INFOSAN share information on important food safety issues of global interest that you have found useful?
- Have you shared information with other network members?
- What have your experiences been like in terms of using information you received through INFOSAN or sharing information with others in the network?
- Has INFOSAN promoted partnership and collaboration between countries and networks? If yes, how so? Was it useful?
- What has been your experience when interacting with other INFOSAN members during a food safety emergency?
- Has INFOSAN helped countries strengthen their capacity to manage food safety risks "from farm to table"? If yes, how so?
- How could INFOSAN improve the way it functions in order to better achieve its objectives?

Relevance:

- To what extent are the objectives of INFOSAN still valid?

For peer review only

- Are the activities and outputs of INFOSAN consistent with the overall goal and the attainment of its objectives?
- Are the activities and outputs of INFOSAN consistent with the intended impacts and effects?

Effectiveness:
- To what extent have the objectives been achieved or are likely to be achieved?
- What are the major factors influencing the achievement or non-achievement of the objectives?

Efficiency:
- Were INFOSAN activities cost-efficient?
- Were objectives achieved in a timely manner?
- Were INFOSAN activities delivered in the most efficient way compared to alternatives?

Impact:
- What has been the biggest impact that INFOSAN has made?
- What real difference has INFOSAN made to members? To the safety of the global food supply? To the improvement of global food safety systems?
- How have populations' health been affected?

Sustainability:
- What are the major factors which influence the achievement or non-achievement of the sustainability of INFOSAN?

Theme 4) Assessing value of INFOSAN

Preface: INFOSAN has been in operation since 2004, and this study is the first of its kind to try and determine if the network has value to members in a robust and rigorous way.

Questions about experiences:

- What has participation in INFOSAN been a valuable experience for you? How so?
- Have you been able to connect with other food safety professionals to exchange information related to food safety?
- Has information received through INFOSAN led you to implement risk management measures in your country to prevent foodborne illness? Can you tell me about some specific events?
- Do you believe that participation in INFOSAN has reduced foodborne illness in your country? How so? How do you think this could be measured?
- Do you think participation in INFOSAN will reduce foodborne illness in your country the future? Globally?
- Does the utilization of the INFOSAN Community Website add value to the network activities?
- Do you feel like you are part of a global community? Is that something you value?

Is there anything else about your experience as an INFOSAN Member that you would like to share with me at this time?

For peer review only

Supplementary File 11 – Information Email #5 – Results from Phase 3 and study conclusion (including invitation to attend Webinar #4)

To be sent by the INFOSAN Secretariat (infosan@who.int) on behalf of the researcher (cc: c.savelli@lancaster.ac.uk)

Health & Medicine | Lancaster University

Information Email #5

A mixed-methods exploration into the experience of members of the International Food Safety Authorities Network (INFOSAN): Phase 3 results and study conclusion

---

Dear INFOSAN Member,

My name is Carmen Savelli and in May 2018 I first contacted you to let you know that I was conducting this study as a student in the PhD Public Health programme at Lancaster University, Lancaster, United Kingdom. You may also know me as one of the Technical Officers working as the INFOSAN Secretariat at the World Health Organization (WHO) in Geneva, Switzerland. The study has now concluded.

What was the overall study about?
The purpose of this study is to interrogate INFOSAN in order to describe and explore the experiences of members and better understand the role of the network in mitigating the burden of foodborne illness around the world. The study will examine access to and usage of the INFOSAN Community Website, explore barriers and facilitators to active participation in INFOSAN, determine perceptions about the utility of INFOSAN to mitigate foodborne illness, and scrutinize if and how participation in this network creates value for members.

The study was designed in three phases: Phase 1 has examined access and usage patterns of the INFOSAN Community Website; Phase 2 involved the completion of an online survey by INFOSAN members; and Phase 3 involved individual interviews conducted online using WebEx.

What are the results from Phase 3?

To be inserted

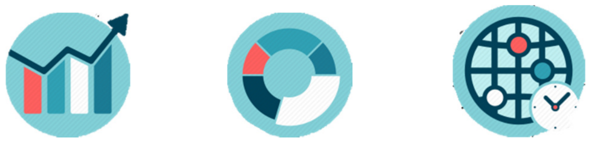

Continued on next page…

Continued from previous page…

How can I find out more about the results from Phase 3 and the overall study conclusion?
Attend the upcoming Webinar:

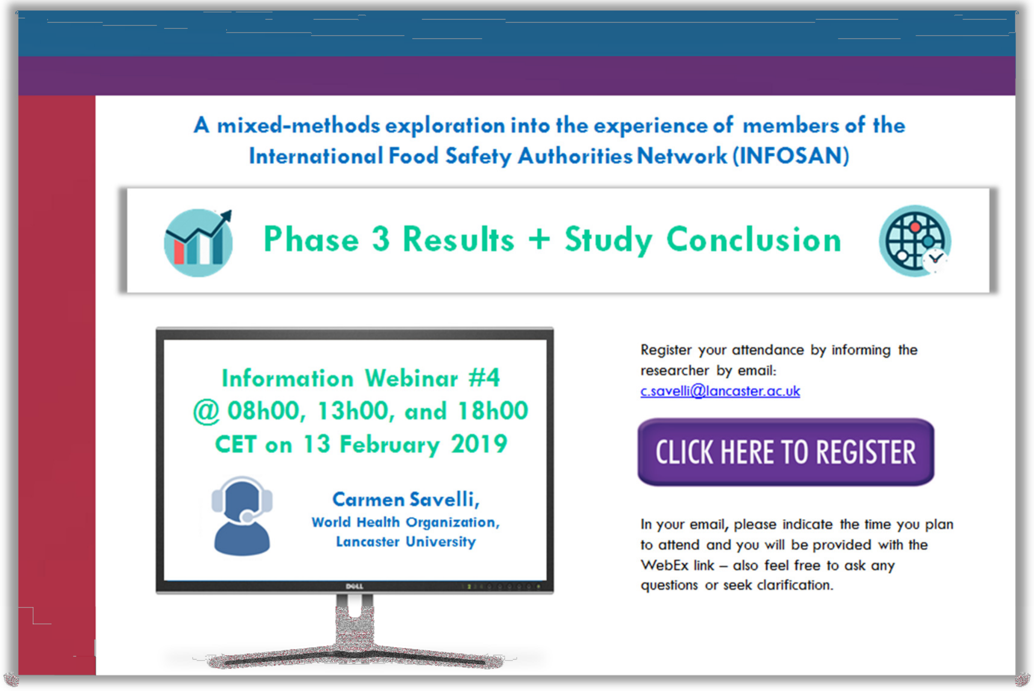

What are the next steps?

The results of this study have been shared with the INFOSAN Secretariat and other senior staff at the World Health Organization for their consideration. Improving the capacity of INFOSAN members to better prevent, detect, and respond to food safety emergencies is a priority.

Any forthcoming publications linked to the study results will be announced by email to the INFOSAN members by the INFOSAN Secretariat.

If you have any additional feedback about this study you are welcome to contact the researcher (c.savelli@lancaster.ac.uk).

If you have other feedback about INFOSAN in general that you wish to share with WHO, please contact the INFOSAN Secretariat directly (infosan@who.int).

Many thanks to all INFOSAN members who contributed time and data to this study. Your participation has been invaluable and is sincerely appreciated.

Kind regards,

Carmen Savelli

BMJ Open

A mixed-methods exploration into the experience of members of the FAO/WHO International Food Safety Authorities Network (INFOSAN): Study Protocol

Journal:	*BMJ Open*
Manuscript ID	bmjopen-2018-027091.R2
Article Type:	Protocol
Date Submitted by the Author:	10-Apr-2019
Complete List of Authors:	Savelli, Carmen; Organisation mondiale de la Sante, Food Safety and Zoonoses; Lancaster University Faculty of Health and Medicine, Health Research<br>Mateus, Ceu; University of Lancaster, Division of Health Research
**Primary Subject Heading**:	Global health
Secondary Subject Heading:	Global health
Keywords:	Information management < BIOTECHNOLOGY & BIOINFORMATICS, Information technology < BIOTECHNOLOGY & BIOINFORMATICS, World Wide Web technology < BIOTECHNOLOGY & BIOINFORMATICS

SCHOLARONE™
Manuscripts

Title: A mixed-methods exploration into the experience of members of the FAO/WHO International Food Safety Authorities Network (INFOSAN): study protocol

Authors: Carmen Joseph Savelli[1,2], Céu Mateus[2]

1) World Health Organization, Geneva, Switzerland
2) Lancaster University, Lancaster, United Kingdom

Corresponding Author: Carmen Joseph Savelli
World Health Organization
Food Safety and Zoonoses
Avenue Appia 20
1211, Geneva, Switzerland
savellic@who.int
+41 22 79 13234

Key words: international cooperation, foodborne diseases, food safety, networking, World Health Organization

Word Count: 6073

For peer review only

Abstract

Introduction: The International Food Safety Authorities Network (INFOSAN) is a global network of national food safety authorities from 188 countries, managed jointly by FAO and WHO, which facilitates the rapid exchange of information during food safety related events. The proposed research will interrogate INFOSAN in order to describe and explore the experiences of members and better understand the role of the network in mitigating the burden of foodborne illness around the world.

Methods: Examined through a community of practice (CoP) lens, a three-phase research design will combine quantitative and qualitative methods (including website analytics in phase 1, online survey administration in phase 1, and semi-structured interviews in phase 3) to elicit a broad and deep understanding of the network operation and member experiences.

Analysis: In phases 1 and 2, quantitative data collected from the INFOSAN Community Website and the online questionnaires will be analysed using descriptive summary statistics. In Phase 3, Interpretative Phenomenological Analysis (IPA) will be utilized to engage in a dialogue with study participants to explore and describe their lived experiences regarding participation in activities related to INFOSAN. An important aspect of the overall analysis will be triangulation of the information collected from each phase including quantitative indicators and qualitative value stories in order to provide a robust understanding of member experience.

Ethics and dissemination: This study has undergone ethical review and has received approval from Lancaster University's Faculty of Health and Medicine Research Ethics Committee as well as the World Health Organization's Ethics Review Committee. Findings from the study will be disseminated as a PhD thesis submitted to Lancaster University. In addition, results of the research shall be submitted for publication to relevant academic or professional conferences and journals or other media including books or websites.

Strengths and limitations of this study

- This study represents the first ever to explore and describe the experiences of all INFOSAN members with respect to their participation in network activities as a means to improve global food safety and prevent foodborne illness, using a range of data collection methods.
- The mixed-method approach will strengthen the credibility of the findings and provide a more complete view and deeper understanding of the experiences of members.
- The main limitation of this study is that the online survey will only be available in English, French and Spanish and interviews will only be conducted in English due to limited resources of the study team.

1. Introduction

In 2015, the WHO reported estimates of the global burden of foodborne diseases for the first time. Together, 31 foodborne hazards are estimated to cause 600-million cases of foodborne disease and 420,000 deaths annually, worldwide[1]. Foodborne diseases are preventable, but ensuring a safe national food-supply requires a robust food control system and coordination among different

For peer review only

government sectors responsible for human health, animal health, agriculture, trade and others. In addition, as a global commodity, food produced in one country can readily cause international outbreaks if contaminated food is exported abroad. Channels of communication on matters of food safety must therefore be well established within and between countries in order to prevent national and international food safety emergencies.[2] It is for these reasons why the WHO launched INFOSAN in 2004. Today, the overall aim of INFOSAN is to halt the international spread of contaminated food, prevent foodborne disease outbreaks, and strengthen food safety systems globally[3]. The four main objectives of INFOSAN are to: 1) Promote the rapid exchange of information during food safety related events; 2) Share information on important food safety related issues of global interest; 3) Promote partnerships and collaboration between countries, and between networks; and 4) Help countries strengthen their capacity to manage food safety emergencies. Using the INFOSAN Community Website (ICW; a secure, online portal), INFOSAN members from around the world exchange information on urgent food safety issues and emerging trends of potential global interest. The ICW also provides a virtual environment to share lessons-learned and allows members to pose questions to one another for the purpose of building knowledge related to food safety.[3]

INFOSAN members have a common identity that is defined by a shared domain of interest. By joining the network, each has committed to taking actions that contribute to a safer global food supply by engaging in joint activities and discussions to facilitate knowledge transfer and exchange (KTE). Common responsibilities are also shared by members, as defined by the INFOSAN Secretariat. Combined, these common responsibilities and activities create a sense of community, and are undertaken with the intention of facilitating the application of best practices to improve food safety. In addition, INFOSAN members are each practitioners in their respective countries, some as food regulators, or risk analysts, or epidemiologists or other professionals. While their focus may be different, the uniting factor is that their practice, in some respect, aims to make contributions towards the reduction of foodborne illness. It is the shared domain, community, and practice which allows for INFOSAN to be understood as a Community of Practice (CoP).[4] A CoP is a group of people sharing a particular concern, problem or passion for an area and who deepen their knowledge and expertise by

For peer review only

learning from one another and interacting on a regular basis.[4] Such interactions may occur in person or through technology-mediated means, as with INFOSAN.

A growing body of research suggests that KTE can be effectively fostered within CoPs, leading to the uptake and application of best practices by individuals and teams in various sectors including health, business, and beyond.[5] Multiple systematic reviews[6, 7, 8,9] suggest that fostering a virtual or electronic CoP among professionals in public health is useful for encouraging KTE which translates into adoption of evidence-based best practices, and by extension, improved public health. Rajic et al.[10] have described the benefits of facilitating KTE among food safety professionals working at the intersection of agriculture and health. Together, the literature suggests that a CoP like INFOSAN, connecting food safety and public health professionals from around the world, is an appropriate tool to facilitate KTE in this area. However, while INFOSAN has been operating for nearly 15 years to facilitate the aforementioned activities among its members, it has never been characterized or examined as a functional CoP and its value, as understood from the perspective of its members, has never been determined in a systematic or rigorous way. Furthermore, a paucity of research has been conducted to investigate the attributes and effectiveness of specific tools or CoPs like INFOSAN to facilitate cross-border communication during international food safety events. To date, most of the publications mentioning such tools focus on summarising a particular incident response, rather than explicitly examining the tools that were utilised. However, such reports of international food safety events commonly conclude with recommendations to better utilise existing international networks and communication tools to improve and expedite information exchange.[11, 12, 13, 14, 15, 16, 17] In addition, several published studies have specified the important role that INFOSAN has played in facilitating rapid international communication between government officials that led to the timely implementation of risk management measures during a food safety emergency.[18, 19, 20]

3. Research Aim

The overall aim is to explore and describe the experiences of INFOSAN members with respect to their participation in network activities as a means to improve global food safety and prevent foodborne illness.

4. Research Objectives

For peer review only

1) Assess the functioning of INFOSAN as a community of practice by obtaining systematic insights into the characteristics, performance and opinions of members.
2) Gain a broad and deep understanding of members' perceptions of the utility of INFOSAN as a global communication tool for KTE and the prevention of foodborne illness in their respective country.
3) Determine if participation in INFOSAN creates value for members and explore the mechanisms through which this may occur.

For peer review only

5. Main research questions

1) How is the ICW being used to support the network activities?
2) What are the barriers and enablers to active participation in INFOSAN?
3) Do members of INFOSAN believe that participation in the network has prevented (or will prevent) foodborne illness in their country?
4) Does participation in INFOSAN create value for members and if so, through what mechanisms does this occur?

6. Methodology and methods

6.1. Philosophical underpinnings

The theoretical framework for exploring experiences in CoPs proposed by Wenger et al. [21] (2011) is utilized for this study and has its roots in critical realism. Critical realism is a philosophical perspective that accepts the existence of stable and enduring features of reality independently of one's ability to perceive them and therefore should be measured as a sum of different perspectives.[22] This concept represents the ontological perspective of critical realism (where ontology should be understood as the philosophical study of being). Furthermore, critical realism asserts that social phenomena and their meanings are continually being created by social actors (i.e. the philosophical concept of constructivism) and may differ depending on one's perspective (i.e. the philosophical concept of relativism). Constructivism and relativism represent the epistemic orientation of critical realism (where epistemology is the philosophical study of knowledge and how one knows).[23]

The importance of utilizing quantitative indicators together with qualitative narratives from different perspectives in a community is a concept described by Wenger et al.[21] in order to understand what kind of experiences create value within CoPs. Quantitative indicators can be readily obtained from most virtual CoPs by measuring the number of unique users logging in to a web portal over time or the number of postings within a discussion forum, for example. However, Wenger et al.[21] suggest that relying on such indicators alone would require too many assumptions to be made to accurately find meaning in experiences and determine value. Such indicators should rather serve as a point of reference from which value-creation stories can be elicited from members directly in order to provide a more robust understanding of experience. Likewise, examining only qualitative narratives from members would ignore the opportunity to crosscheck information with indicators to determine how perceptions and actions correspond. It is this acknowledgement of multiple perspectives of reality which demonstrates congruence with critical realism. In taking this approach, discrepancies and correspondences can be observed and both grounded narratives and aspirational narratives become illuminated (where grounded narratives are those for which value-creation stories are supported by quantitative indicators and aspirational narratives are not).[21]

With an understanding of INFOSAN as a functional CoP, and with an appreciation for the critical realist perspective that underpins its examination, it is incumbent upon the researcher of the proposed study to take a mixed-method approach in order to strengthen the credibility of the findings and provide a more complete view and deeper understanding of the experiences of members. The utilization of such an approach for the investigation of experiences of online community members is strongly supported by a growing body of literature.[24, 25, 26]

6.2. Setting

The study will be conducted by the researcher from within the Department of Food Safety and Zoonoses (FOS) at the headquarters of the WHO in Geneva, Switzerland (where the INFOSAN Secretariat is based), however the true setting is global since INFOSAN membership spans 188 countries.

6.3 General comments on sampling

For peer review only

For each phase of the research, participants will only include registered INFOSAN members. These individuals have been officially designated by their national government and are all registered on the ICW (N=500). INFOSAN membership includes both men and women in approximately equal proportions.

6.4 General comments on recruitment

Written permission to conduct the proposed research on INFOSAN has been received from the Director of FOS at the WHO, where the INFOSAN Secretariat is based. To announce the launch of the study, all members of INFOSAN will receive by email, written introductory information about the proposed research (Information Email #1; supplementary file #1), including an invitation to attend an online seminar (i.e. webinar), delivered by the researcher, to find out more information about the study and ask any questions or seek clarification. This webinar, like all future webinars discussed in this proposal, will be scheduled at three different times to allow INFOSAN members across different time zones to participate. The webinar will be delivered using the secure online conferencing tool, WebEx. The live webinars will not be recorded, but a recording will be made by the researcher alone and will be made available to INFOSAN members to view in case they would like to do so at their convenience.

6.5. Phase 1: Analysis of INFOSAN Community Website access and usage

6.5.1. Sampling, recruitment and consent

Information Email #1 will explain the three different phases of the proposed research and remind INFOSAN members that data analysed in Phase 1 of the study will be extracted from the ICW in accordance with the Terms and Conditions of Use that each member consented to when they registered online. The relevant text within the Terms and Conditions of Use, reads as follows: "Utilization of website analytics tools or other methods will be applied periodically to summarize members' access to and usage of the INFOSAN Community Website for monitoring, evaluation and research purposes." As such, data pertaining to all members of INFOSAN with respect to their access to and usage of the ICW, will be utilised in Phase 1 of the study unless a member expressly indicates their wish to be excluded. Information Email #1 (and related webinars) will ensure that INFOSAN members understand that any members not wishing to have their website access and usage data

For peer review only

utilised for the purpose of this study will have two weeks to make this indication by email to the researcher. All those members who do not otherwise object will be recruited for Phase 1. Those who are recruited in Phase 1 will have a further two weeks to opt out of the study, after which time this will no longer be possible due to aggregation of the data.

6.5.2. Data collection and analysis

Access to the ICW is granted to the researcher in his capacity as a staff member at WHO and approval for use in this research has been granted by the Director of FOS, WHO. Data from the ICW will be collected retrospectively for the period between February 2012 (when the website was launched) and December 2018. Information from all recruited members concerning the following variables will be downloaded from the website, anonymised and exported into Microsoft Excel and SPSS for analysis: type of member, sex, country, languages spoken, government sector, primary function (i.e. risk assessment, risk communication, or risk management), and areas of scientific expertise. These data have all been automatically collected and stored in the internal website database at the time of member registration. Additional data about length of membership, last access to the website, and discussion thread initiations, responses and views will also be collected and exported for analysis. Once collected, all anonymised data will be analysed using descriptive summary statistics, which will allow for stratification by a number of variables including type of member, geographic region, and length of membership. Analysing these data will provide an objective, foundational layer of information about the experiences of all members and will be triangulated with data from Phases 2 and 3 to determine if members' reported attitudes and experiences reflect their online behaviours. In addition, triangulated data will also allow for INFOSAN to be described with respect to its stage of community development according to Wenger et al.[4], taking into account its structuring characteristics as described by Dubé, Bourhis and Jacob.[27]

6.6. Phase 2: Administration of an online survey

6.6.1. Sampling, recruitment and consent

Upon conclusion of data collection and analysis in Phase 1, all members of INFOSAN will receive Information Email #2 (supplementary file #2) which will include indicative results from Phase 1 and an invitation to attend a webinar, delivered by the researcher, to learn more about the results

For peer review only

from Phase 1 and to provide further details about Phase 2. Immediately following the webinar, all INFOSAN members (N=500) will receive Information Email #3 (supplementary file #3), including the invitation to participate in Phase 2 of the study and containing a link to an online survey. Only those who express consent will be recruited as participants for Phase 2. No consent form will be collected because that would compromise anonymity, however, the email will explain that by clicking on the link, the individual confirms that they have read the introductory information and understand what is expected of them as a participant in this phase of the study (full details in supplementary file #3).

6.6.2. Data collection and analysis

Recruited participants will be requested to complete an online questionnaire that will take between 30 and 45 minutes. Responses to the questionnaire are intended to provide systematic insights into the characteristics, performance and opinions of INFOSAN members and contribute to a broader understanding of their experiences. The questionnaire will consist of questions from the Community Assessment Toolkit (CAT) [28] as well as a supplemental set of questions, tailored specifically to INFOSAN members. During the development of the CAT, Verburg and Andriessen[28] demonstrated that the methodology for its development was based on dominant theories of CoPs and group dynamics. The CAT was pilot tested and studied with seven CoPs (n=271) to enable reliability tests and scale analysis. Cronbach's alpha for the scales ranged from .59 to .91 and as the items referred to quite separate goals, Verburg and Andriessen[28] reported these values acceptable. Utilizing the CAT in this study will enable future comparative research between CoPs that have been assessed with the same tool (e.g. Roberts[25]). However, given the unique nature of INFOSAN and the specific objectives of this study, it is also necessary to develop a short set of supplemental questions to examine the experiences of INFOSAN members that are unique to this particular CoP. A preliminary set of supplemental questions has been inserted to the appropriate sections of the CAT questionnaire. These supplemental questions will be reviewed for content validity by a panel of six experts consisting members of the INFOSAN Advisory Group since they are familiar with the constructs that the supplemental questions are designed to measure. The expert panel will judge whether the supplemental questions adequately measure the construct they are intended to assess, and whether

For peer review only

these supplemental questions are indeed sufficient to measure the domain of interest. A Content Validity Index (CVI) will be computed for each supplementary item and items with a CVI of 0.78 or higher will be considered evidence of good content validity.[29] Based on feedback from the expert panel, the supplemental questions will be revised prior to pilot testing. After incorporating any suggestions from INFOSAN Advisory Group members, FAO/WHO Regional Food Safety Advisors/Officers will be requested to pilot test the supplemental questions. As a measure of reliability, internal consistency will be estimated using the coefficient alpha, also known as Cronbach's alpha. Determining the internal consistency will reflect the extent to which the questionnaire items are inter-correlated, or whether they are consistent in measurement of the same construct.[30]

Because the questionnaire (including the CAT questions and the supplementary questions) will be disseminated to INFOSAN members in 188 countries, it will be adapted from English into French and Spanish in order to encourage a higher response rate. The aim of the adaptation process is to achieve different language versions of the English instrument that are conceptually equivalent in both French and Spanish. The instrument should be equally natural and acceptable and should practically perform in the same way, with a focus on cross-cultural and conceptual equivalence, rather than on linguistic/literal equivalence. A well-established method to achieve this goal is to use forward-translations and back-translations.[31] This method has been refined over the course of several WHO studies to result in the following process which will be undertaken to adapt the questionnaires into French and Spanish.[31] First, one native Spanish speaker and one native French speaker from the Department of Food Safety and Zoonoses at WHO will conduct forward translations of the entire questionnaire. These translators will be health professionals, familiar with the terminology of the area covered by the instrument. Next, two bilingual expert panels will be convened to identify and resolve the inadequate expressions/concepts of the translation, as well as any discrepancies between the forward translation and the English versions of the questions. Next, using the same approach as outlined in the first step, the questionnaire will then be translated back to English by an independent translator (from central translation services at WHO), whose mother tongue is English and who has no knowledge of the questionnaire. As in the forward-translation, emphasis in the back-translation should

be on conceptual and cultural equivalence and not linguistic equivalence. Discrepancies should be discussed with the researcher and further work (forward translations, discussion by the bilingual expert panel, etc.) will be iterated as many times as needed until a satisfactory version is reached. This version will then be pre-tested with a group of 20 interns at WHO including 10 native French speakers and 10 native Spanish speakers. A call for volunteers will be disseminated through the WHO intern mailing list. Pre-test respondents will be administered the questionnaire and then systematically debriefed. This debriefing will ask respondents what they thought the questions were asking, whether they could repeat the questions in their own words, and what came to their minds when they heard a particular phrase or term. The debriefing will take the form of a focus group, organized at WHO. The adaptation process followed will be traceable through a number of documents including: 1) initial forward versions; 2) a summary of recommendations by the expert panels; 3) the back-translations; 4) a summary of problems found during the pre-testing of the instrument and the modifications proposed; 5) the final version; and 6) a description of the samples used in this process (i.e. the composition of the expert panel and the pre-test respondent samples). The process of expanding and adapting the questionnaire is summarized in supplementary file #4.

Quantitative data collected from the online questionnaires will be analysed using descriptive summary statistics, allowing for stratification by a number of variables including type of member, geographic region, length of membership, etc. A variety of techniques for univariate, bivariate and multivariate analysis using SPSS will be employed in order to examine patterns and relationships between variables. Depending on the response rate to the survey, the researcher may need to adjust for non-response bias in order to generalise the results to the entire network.[32] The researcher is a native English speaker with a working knowledge of French and basic knowledge of Spanish. As such, for the very few instances where an open text response is an open, answers provided in French or Spanish will be translated by the researcher with the aid of Google translate, if necessary.

6.7. Phase 3: Semi-structured interviews

6.7.1. Sampling, recruitment and consent

Upon conclusion of data collection and analysis in Phase 2, all members of INFOSAN will receive Information Email #4 (supplementary file #5) which will include indicative results from Phase

For peer review only

2 and an invitation to attend a webinar, delivered by the researcher, to learn more about the results from Phase 2 and to provide further details about Phase 3. The email and webinar will also include a request that members interested in participating in Phase 3 indicate this by emailing the researcher. The online survey in Phase 2 will also serve as a recruitment tool for Phase 3 since a concluding statement on the survey will indicate that members interested in participating in Phase 3 of the research should contact the researcher separately by email.

A minimum of six and a maximum of 12 participants will be included in the study sample, aiming for two participants from each of the six geographic regions delineated by WHO. The purpose is not to be representative of the entire network, however, because INFOSAN is global, including participants from each region may reveal a richer pool of experience than if all members were selected from a single region. In addition, a sample size within this range should allow for the examination of similarities and differences between individuals, without producing an overwhelmingly large amount of qualitative data that cannot be managed within the confines of the study timeline. This sample of participants will be restricted to those INFOSAN members who have been registered members for a minimum of two years at the time of their interview to ensure they have a reasonable level of experience with the network from which to draw. The sample will be limited to those members who speak English due to limited funding for research conduct (including for translation and interpretation) and limited time for collecting and analysing data in other languages. In the event that at least one member per WHO region does not volunteer after receiving Information Email #4, follow-up emails will be sent to those members from the regions where volunteers are still needed, to indicate as such.

If more than two people from a single WHO region indicate their interest in participating in Phase 3, the two people who have been members the longest will be selected and notified by email from the researcher, providing they are not from the same country**.** Any individuals who volunteer for Phase 3 but who are not selected will be emailed individually by the researcher and provided with an explanation about how the selection was made (supplementary file #6).

Prior to commencing their interview, INFOSAN members who volunteer and are recruited for Phase 3 of the study will need to have read, signed, and returned by email to the researcher, the Phase

For peer review only

3 consent form as detailed in supplementary file #7. Immediately before the interview, the researcher will ensure the participants are aware they can choose to withdraw from the study at any time, for any reason, before or during their interview and withdraw their data up to two weeks after their interview.

6.7.2. Data Collection and Analysis

Recruited participants will be requested to participate in a semi-structured interview conducted online using the secure tool, WebEx, because participants are anticipated to be located in various countries around the world (no face-to-face interviews will be conducted). The interviews will be scheduled to last between 45 minutes and one hour. The same four themes explored in Phase 2 will be explored during the interviews, and the discussion will focus on answering the related questions of 'how?' and 'why?' instead of just 'what?' as elaborated in the interview guide in supplementary file #8 (results from Phase 1 and/or 2 may lead to the addition or deletion of certain questions from the current interview guide). The researcher will follow Interpretative Phenomenological Analysis (IPA; a qualitative research approach which is used to explore and examine personal lived experiences[33]) to engage in a dialogue with study participants to explore and describe their lived experiences regarding participation in activities related to INFOSAN. Such a method requires a flexible data collection instrument[33] and therefore, the semi-structured format of the interviews will be conversational in style, allowing the researcher and participant to engage in a dialogue where questions can be modified depending on responses. This format also enables the researcher to prompt further elaboration in certain areas of interest identified by participants, allowing for more flexibility than a structured interview.[32] The interviews will be audio recorded and then transcribed by the researcher. Audio recordings will be made using WebEx and immediately downloaded, password protected and encrypted on a laptop. Audio recorded data will be anonymised as far as is possible (given the nature of audio data) by saving the file with an de-identified tag (e.g. Participant #1 Region X). Prior to recording, the researcher will remind the participant that they can refrain from using names of people and places to the extent possible when answering questions, to assist with anonymization. Once recording has started, the researcher will not use the participant's name during the interview. Transcripts will be anonymized by replacing identifying names of people or places with a de-identified tag (e.g. Participant #1 Region X). Analysis will involve multiple readings of the interview

For peer review only

transcripts, coupled with note taking, followed by transformation of notes into emergent themes, and finally, seeking relationships and clustering themes.[32]

An important aspect of the overall analysis will be triangulation of the information collected from each phase including quantitative indicators and qualitative value stories. Anonymised information and quotations from participant interviews will be reported, representing the limits to confidentiality. The combined strengths of quantitative and qualitative methods can contribute to improved study validity, credibility, and overall integrity and provide a broad and deep understanding of members' experiences.[32, 34, 35] The indicative results from Phase 3, along with a triangulated analysis of data from all three phases will be communicated to INFOSAN members through Information Email #5 (supplementary file #9) and a final webinar, delivered by the researcher.

7. Patient and Public Involvement

Patients were not involved in the design of this study. The first author has had conversations with several INFOSAN members in finalizing this study design. These conversations have followed a presentation of the research design at a regional meeting of INFOSAN members in Miami, USA in November 2017 where INFOSAN members from approximately 30 countries were present. In addition, a more detailed presentation of the study design was delivered at a meeting of 8 INFOSAN members in Geneva, Switzerland in December 2017, where further discussion has contributed to the finalization of the study design, including the overall aim, objectives and research questions. The questionnaire will also be vetted by several target participants (selected because they are also part of the INFOSAN Advisory Group) as described in supplementary file #4. The results of each phase of the study will be communicated to study participants via email and through webinars delivered by the first author.

8. – Ethical issues

8.1. – Data management and storage

In accordance with the General Data Protection Regulation (GDPR) and the (UK) Data Protection Act 2018, all electronic data will be anonymised and stored onto encrypted password-

For peer review only

protected storage devices, including data keys and a laptop computer) provided by the WHO. These encrypted files will utilise meaningful file names and version numbers and will be accompanied by a Readme file. Personal identifiers will be kept separately from anonymised data in either encrypted computer storage (for electronic files) or in a locked filing cabinet (for paper files) and destroyed following the completion and acceptance of the PhD thesis. Only the researcher and his supervisors will have access to the anonymised data for the duration of the study. Electronic copies of transcripts will be transferred in person by the researcher from WHO headquarters to Lancaster University on encrypted, password-protected storage devices and then archived for 10 years in secure encrypted storage on the Lancaster University server, after which time they will be destroyed. Storage and destruction of data will be done by Lancaster University's designated Data Protection Officer. The researcher may need to modify the aforementioned arrangements related to data management and storage and privacy protection measures to ensure that provisions of the GDPR are accommodated in case such provisions are supplemented, interpreted or clarified while the study is being conducted.

8.2. – Conducting insider research

By the nature of this work-based research project, the researcher is an insider, investigating an issue that examines, in broad terms, the operation of an organizational programme. The researcher is therefore an agent of his organization as a technical officer at WHO and also an agent of Lancaster University as a PhD student. As such, the ethical considerations for the design of this research project have been carefully made from the insider researcher perspective.

In addition to approval being granted by the Director of FOS to conduct this research, the proposed study has been subject to scrutiny and approval by the Faculty of Health and Medicine Research Ethics Committee at Lancaster University and the WHO Research Ethics Board before it could commence. This process involved technical review by an external scientific committee of experts. In addition, the conduct of the research will be governed by the WHO Code of Ethics and Professional Conduct and the WHO Code of Conduct for Responsible Research which both emphasise the need for all research to be conducted with integrity, accountability, independence, impartiality, respect, and professional commitment.

For peer review only

While there are several positive aspects to being an insider researcher (e.g. informed perspective, ability to implement study recommendations directly, etc.), the potential conflicts of interest must be carefully considered, acknowledged and addressed. As members of INFOSAN are familiar with the researcher in his role at WHO, he will need to ensure transparency and clarity that the proposed research is being conducted as part of a PhD study. It will also be necessary to ensure that despite having access to additional data or information, that only those data collected with the expressed consent of participants is utilised and reported on for the purposes of this study. INFOSAN members must be assured that neither their participation nor abstention will impact their future treatment as an INFOSAN member or the technical support provided to them or their agency by the WHO.

Acknowledging the role of the researcher as an insider is congruent with the methodology used to conduct this research on INFOSAN through a CoP lens. IPA as a research methodology is concerned with carefully detailing the lived experience of individuals. As explained by Guldberg and Mackness[26], utilizing IPA to understand experience aligns with a CoP lens since the focus of analysis is on the interpretations of members and the values they attribute to them. In addition, IPA acknowledges and embraces the role of the researcher's interpretation and understanding of members' lived experiences. The researcher, through his insider role on the INFOSAN Secretariat, plays an important part in connecting, communicating and facilitating interaction among members, and therefore his familiarity with the participants and ability to provide expertise in the interpretation and understanding of members' experiences should be considered an asset.

While the role of insider researcher can present some potential conflicts of interest, transparency in process, due permissions from senior WHO staff, and assurances given to INFOSAN members, should ensure this research is conducted to the highest ethical standard. In addition, several techniques well-known to insider researchers will be employed to aid in accurately understanding and documenting the experiences of INFOSAN members, including the practice of reflexivity. The practice of reflexivity will involve active engagement of the self and questioning of the researcher's own perceptions in order to expose their contextualized nature.[36] A diary will be kept by the researcher during data collection and analysis to document reflexivity including predictions of

For peer review only - http://bmjopen.bmj.com/site/about/guidelines.xhtml

outcomes for each phase of the study and how they relate to one another. The diary will not include any identifying information related to participants to ensure anonymity of participants and any reflections on interviewed participants will reference a de-identified tag (e.g. Participant #1 Region X). The diary will be stored in a locked cabinet in the main researcher's office when not being used in case it contains any sensitive information.

9. Dissemination plans

Results from this study can be utilized directly by the INFOSAN Secretariat at WHO in order to plan future interventions intended to encourage and support active participation in this important global network. In addition, results from this study may also contribute new knowledge regarding the coordination of international virtual networks and communities of practice in the realm of global health. The researcher will therefore disseminate findings from the study in the following ways: 1) via webinars, open to all INFOSAN members following each of the three phases of the study; 2) as an oral presentation to staff within FOS, WHO; and 3) as a PhD thesis submitted to Lancaster University. In addition, results of the research shall be submitted for publication to relevant academic or professional conferences and journals or other media including books or websites.

Authors' contributions: CS conceived the original idea, designed the study, drafted the manuscript and approved the final document. CM drafted the manuscript and approved the final document. CS is a staff member of the World Health Organization. The authors alone are responsible for the views expressed in this publication and they do not necessarily represent the views, decisions or policies of the World Health Organization.

Data Availability Statement: All data relevant to the study will be anonymized and uploaded as supplementary information upon publication of the results in a peer-reviewed journal. A copy of the questionnaire can be obtained on request from the first author.

Funding statement: This study is funded by the World Health Organization.

Competing interests: None

For peer review only

References

1. World Health Organization. (2015). *Global burden of foodborne disease report*. Retrieved from: http://apps.who.int/iris/bitstream/10665/200046/1/WHO_FOS_15.02_eng.pdf?ua=1.

2. World Health Organization. (2014). *Advancing food safety initiative: strategic plan for food safety including foodborne zoonoses 2013-2022*. Retrieved from: http://www.who.int/foodsafety/publications/strategic-plan/en/.

3. World Health Organization. (2016). *INFOSAN Activity Report 2014/2015*. Retrieved from: http://apps.who.int/iris/bitstream/10665/246204/1/9789241510592-eng.pdf.

4. Wenger, E., McDermott, R. and Snyder, W. (2002). *Cultivating communities of practice*. Boston, Mass.: Harvard Business School Press.

5. Ho, K., Jarvis-Selinger, S., Norman, C., Li, L., Olatunbosun, T., Cressman, C., & Nguyen, A. (2010). Electronic communities of practice: Guidelines from a project. *Journal Of Continuing Education In The Health Professions*, *30*(2), 139-143. http://dx.doi.org/10.1002/chp.20071

6. Kothari, A., Hovanec, N., Hastie, R., & Sibbald, S. (2011). Lessons from the business sector for successful knowledge management in health care: A systematic review. *BMC Health Services Research*, *11*(1). http://dx.doi.org/10.1186/1472-6963-11-173

7. Mairs, K., McNeil, H., McLeod, J., Prorok, J., & Stolee, P. (2013). Online strategies to facilitate health-related knowledge transfer: a systematic search and review. *Health Information & Libraries Journal*, *30*(4), 261-277. http://dx.doi.org/10.1111/hir.12048

8. Quinn, E., Huckel-Schneider, C., Campbell, D., Seale, H., & Milat, A. (2014). How can knowledge exchange portals assist in knowledge management for evidence-informed decision making in public health?. *BMC Public Health*, *14*(1). http://dx.doi.org/10.1186/1471-2458-14-443

9. Barbour, L., Armstrong, R., Condron, P., & Palermo, C. (2018). Communities of practice to improve public health outcomes: a systematic review. Journal Of Knowledge Management, 22(2), 326-343. doi: 10.1108/jkm-03-2017-0111

10. Rajić, A., Young, I., & McEwen, S. (2013). Improving the Utilization of Research Knowledge in Agri-food Public Health: A Mixed-Method Review of Knowledge Translation and Transfer. *Foodborne Pathogens And Disease*, *10*(5), 397-412. http://dx.doi.org/10.1089/fpd.2012.1349

11. Einöder-Moreno, M., Lange, H., Grepp, M., Osborg, E., Vainio, K., & Vold, L. (2016). Non-heat-treated frozen raspberries the most likely vehicle of a norovirus outbreak in Oslo, Norway, November 2013. *Epidemiology And Infection*, *144*(13), 2765-2772. http://dx.doi.org/10.1017/s0950268816000194

12. Guzman-Herrador, B., Nilsen, E., Cudjoe, K., Jensvoll, L., Kvamme, J., & Lindegård Aanstad, A. et al. (2013). A Shigella sonnei outbreak traced to imported basil – the importance of good typing tools and produce traceability systems, Norway, 2011. *Eurosurveillance*, *18*(49), 20650. http://dx.doi.org/10.2807/1560-7917.es2013.18.49.20650

13. Inns, T., Ashton, P., Herrera-Leon, S., Lighthill, J., Foulkes, S., & Jombart, T. et al. (2016). Prospective use of whole genome sequencing (WGS) detected a multi-country outbreak of Salmonella Enteritidis. *Epidemiology And Infection*, *145*(02), 289-298. http://dx.doi.org/10.1017/s0950268816001941

14. Knoblauch, A., Bratschi, M., Zuske, M., Althaus, D., Stephan, R., & Hächler, H. et al. (2015). Cross-border outbreak of Salmonella enterica ssp. enterica serovar Bovismorbificans: multiple approaches for an outbreak investigation in Germany and Switzerland. *Swiss Medical Weekly*. http://dx.doi.org/10.4414/smw.2015.14182

15. Nygård, K., Lassen, J., Vold, L., Andersson, Y., Fisher, I., & Löfdahl, S. et al. (2008). Outbreak of Salmonella Thompson Infections Linked to Imported Rucola Lettuce. *Foodborne Pathogens And Disease*, *5*(2), 165-173. http://dx.doi.org/10.1089/fpd.2007.0053

16. Pezzoli, L., Elson, R., Little, C., Yip, H., Fisher, I., & Yishai, R. et al. (2008). Packed with Salmonella —Investigation of an International Outbreak of Salmonella Senftenberg Infection Linked to Contamination of Prepacked Basil in 2007. *Foodborne Pathogens And Disease*, *5*(5), 661-668. http://dx.doi.org/10.1089/fpd.2008.0103

17. Rebolledo, J., Garvey, P., Ryan, A., O'donnell, J., Cormican, M., & Jackson, S. et al. (2013). International outbreak investigation of Salmonella Heidelberg associated with in-flight catering. *Epidemiology And Infection*, *142*(04), 833-842. http://dx.doi.org/10.1017/s0950268813001714

18. Acciari, V., Iannetti, L., Gattuso, A., Sonnessa, M., Scavia, G., Montagna, C., et al. (2015). Tracing sources of Listeria contamination in traditional Italian cheese associated with a US outbreak: investigations in Italy. *Epidemiology And Infection*, *144*(13), 2719-2727. http://dx.doi.org/10.1017/s095026881500254x

19. Gossner, C., Schlundt, J., Ben Embarek, P., Hird, S., Lo-Fo-Wong, D., & Beltran, J. et al. (2009). The Melamine Incident: Implications for International Food and Feed Safety. *Environmental Health Perspectives*, *117*(12), 1803-1808. http://dx.doi.org/10.1289/ehp.0900949

20. Khardori, N. (2012). A Multistate Outbreak of Hepatitis A Associated With Semidried Tomatoes in Australia, 2009. *Yearbook Of Medicine*, *2012*, 101-102. http://dx.doi.org/10.1016/j.ymed.2012.08.004

21. Wenger, E., Trayner, B., and de Laat, M. (2011). Promoting and assessing value creation in communities and networks: a conceptual framework. Rapport 18, Ruud de Moor Centrum, Open University of the Netherlands.

22. Fade, S. (2004). Using interpretative phenomenological analysis for public health nutrition and dietetic research: a practical guide. *Proceedings Of The Nutrition Society*, *63*(04), 647-653. http://dx.doi.org/10.1079/pns2004398

23. Maxwell, J. (2012). *A realist approach for qualitative research*. Thousand Oaks, CA: SAGE.

24. de Laat, M., & Lally, V. (2003). Complexity, theory and praxis: Researching collaborative learning and tutoring processes in a networked learning community. *Instructional Science*, 31, pp. 7-39.

25. Roberts, G. (2015). Communities of practice: Exploring enablers and barriers with school health clinicians. *Canadian Journal Of Occupational Therapy*, *82*(5), 294-306. http://dx.doi.org/10.1177/0008417415576776

26. Guldberg, K., & Mackness, J. (2009). Foundations of communities of practice: enablers and barriers to participation. *Journal Of Computer Assisted Learning*, *25*(6), 528-538. http://dx.doi.org/10.1111/j.1365-2729.2009.00327.x

For peer review only

27. Dube, L., Bourhis, A., & Jacob, R. (2006). Towards a Typology of Virtual Communities of Practice. Interdisciplinary Journal Of Information, Knowledge, And Management, 1, 069-093. doi: 10.28945/115

28. Verburg, R., & Andriessen, J. (2006). The assessment of communities of practice. *Knowledge And Process Management*, *13*(1), 13-25. http://dx.doi.org/10.1002/kpm.241

29. Polit, D., Beck, C., & Owen, S. (2007). Is the CVI an acceptable indicator of content validity? Appraisal and recommendations. *Research In Nursing & Health*, *30*(4), 459-467. http://dx.doi.org/10.1002/nur.20199

30. Tsang, S., Royse, C., & Terkawi, A. (2017). Guidelines for developing, translating, and validating a questionnaire in perioperative and pain medicine. *Saudi Journal Of Anaesthesia*, *11*(5), 80. http://dx.doi.org/10.4103/sja.sja_203_17

31. World Health Organization. (2018). *Process of translation and adaptation of instruments*. Retrieved from: http://www.who.int/substance_abuse/research_tools/translation/en/.

32. Bryman, A. (2012). *Social research methods (4th Edition)*. Oxford: Oxford University Press.

33. Smith, J, Flowers, P, & Larkin, M. (2009). *Interpretative phenomenological analysis: Theory, method and research*. Thousand Oaks, California: Sage.

34. Allsop, J. (2013). *Researching health: qualitative, quantitative and mixed methods*. Sage.

35. Creswell, JW. (2009). *Research Design: Qualitative, Quantitative and Mixed Methods Approaches*. Sage.

36. Greene, M. (2014). On the inside looking in: Methodological insights and challenges in conducting qualitative insider research. *The Qualitative Report*, 19 (15), 1-13.

For peer review only

Supplementary File 1 – Information Email #1: Introductory information about the study (including the overall Participant Information Sheet) and invitation to attend information webinar #1

To be sent by the INFOSAN Secretariat (infosan@who.int) on behalf of the researcher (cc: c.savelli@lancaster.ac.uk)

Health & Medicine | Lancaster University

Information Email #1

A mixed-methods exploration into the experience of members of the International Food Safety Authorities Network (INFOSAN): Introductory Information

---

Dear INFOSAN Member,

My name is Carmen Savelli and I am conducting this study as a student in the PhD Public Health programme at Lancaster University, Lancaster, United Kingdom. You may also know me as one of the Technical Officers working as the INFOSAN Secretariat at the World Health Organization (WHO) in Geneva, Switzerland.

What is the study about?
The purpose of this study is to interrogate INFOSAN in order to describe and explore the experiences of members and better understand the role of the network in mitigating the burden of foodborne illness around the world. The study will examine access to and usage of the INFOSAN Community Website, explore barriers and facilitators to active participation in INFOSAN, determine perceptions about the utility of INFOSAN to mitigate foodborne illness, and scrutinize if and how participation in this network creates value for members.

Why have I been approached?
You have been approached because the study requires information from registered INFOSAN members.

Do I have to take part?
No. It is completely up to you to decide whether or not you take part. The study is designed in three phases: Phase 1 will examine access and usage patterns of the INFOSAN Community Website; Phase 2 will involve the completion of an online survey; and Phase 3 will involve individual interviews conducted online using WebEx. Taking part in one phase of the study does not require your participation in the other phases.

What will I be asked to do if I take part?
Participation in Phase 1 of the study requires no further action on your part. Data on access and usage of the INFOSAN Community Website has already been collected as part of the ongoing monitoring and evaluation activities of the INFOSAN Secretariat in accordance with the Terms and Conditions of Use of the INFOSAN Community Website. **Any members not wishing to have their website access and usage data utilized for the purpose of this particular study should make this known by email to the researcher within the next two weeks (c.savelli@lancaster.ac.uk).** Those who are recruited in Phase 1 will have a further two weeks to opt out of the study, after which time this will no longer be possible due to aggregation of data.

Participation in Phase 2 of the study requires the completion of an online survey of approximately 30-45 minutes which will explore questions about your experience as an INFOSAN member. Once the survey has been submitted, it will not be possible to opt out because survey responses are anonymous.

Participation in Phase 3 of the study will involve a one-on-one individual interview conducted online using WebEx with the researcher. The interview will last approximately 45-60 minutes and will explore in more detail your experiences as an INFOSAN Member. Those who are recruited for Phase 3 of the study will have two weeks to opt out after which time this will no longer be possible.

Continued on next page…

Continued from previous page…

Will my data be Identifiable?

No. The information you provide will be reported anonymously. Any direct quotes from participants in Phase 3 will be reported anonymously. Quantitative data will be reported by region so that no country-specific details will be reported. The data collected for this study will be stored securely and only the researchers conducting this study will have access to this data:

- Audio recordings will be deleted once the thesis has been examined and accepted
- The files on the computer will be encrypted (that is no-one other than the researcher will be able to access them) and the computer itself password protected for the duration of the study
- At the end of the study, all related electronic information will be archived for 10 years in secure encrypted storage on the Lancaster University server. At the end of this period, they will be destroyed.
- The typed version of your interview will be made anonymous by removing any identifying information including your name. Anonymised direct quotations from your interview may be used in the reports or publications from the study, and neither your name nor your country will be attached to them.
- All your personal data will be confidential and will be kept separately from your interview responses (if you participate in Phase 3). However, there are some limits to confidentiality in Phase 3: if what is said in the interview makes me think that you, or someone else, is at significant risk of harm, I will have to break confidentiality and speak to a member of staff about this. If possible, I will tell you if I have to do this. In addition, whilst every effort will be made, it is not possible for the researcher to ensure confidentiality of your participation in Phase 3 if you choose to connect to the researcher by WebEx on your work premises during the working day. If you volunteer to participate in Phase 3, you are encouraged to connect by WebEx in a private room to conduct your interview, where no one else can see your computer screen or hear your conversation.

For further information about how Lancaster University processes personal data for research purposes and your data rights please visit our webpage: www.lancaster.ac.uk/research/data-protection

What will happen to the results?

The results will be summarised and reported in a PhD thesis and may be submitted for publication in relevant academic or professional conferences and journals or other media including books or websites, pending necessary clearance from WHO. Only those data collected with the expressed consent of participants is utilized and reported on for the purposes of this study. INFOSAN Members will also be invited to participate in webinars after each phase to learn about the results and next steps.

Are there any risks?

There are no risks anticipated with participating in this study. However, if you experience any distress following participation you are encouraged to inform the researcher and contact the resources provided at the end of this sheet. Neither participating nor abstaining will impact your future treatment as an INFOSAN member or the technical support provided to you or your country by the WHO.

Are there any benefits to taking part?

Although you may find participating interesting, there are no direct benefits in taking part. However, some participants may find it a positive experience to reflect on their role as an INFOSAN member as well as their contributions to a safer food supply, and to participate in research which they may feel is of relevance to them, their institution, their country or the global community.

Who has reviewed the project?

This study has been reviewed and approved by the Ethics Review Committee at the World Health Organization and the Faculty of Health and Medicine Research Ethics Committee at Lancaster University.

Where can I obtain further information about the study if I need it?

If you have any questions about the study, please contact the researcher:

Carmen Savelli
c.savelli@lancaster.ac.uk
+41 22 791 3234

Or his primary supervisor in the Department of Health Research at Lancaster University:

Dr Céu Mateus
c.mateus@lancaster.ac.uk
+44 1524 593182

Continued on next page…

Continued from previous page…

Or attend the upcoming webinar:

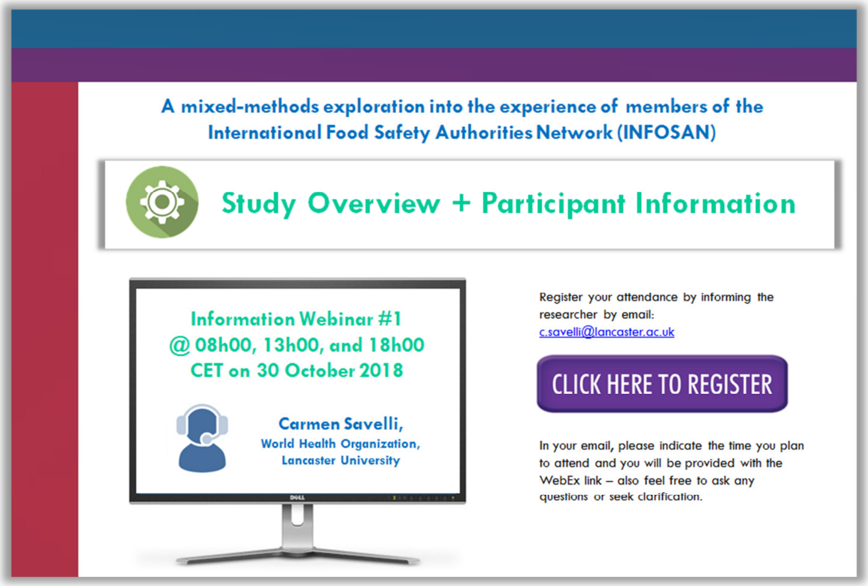

Complaints

If you wish to make a complaint or raise concerns about any aspect of this study and do not want to speak to the researcher or his supervisor, you can contact:

Professor Catherine Walshe
Head of the Division of Health Research
+44 (0)1524 510124
c.walshe@lancaster.ac.uk
Lancaster University
Lancaster
LA1 4YW

If you wish to speak to someone outside of the Public Health Doctorate Programme, you may also contact:

Professor Roger Pickup Tel: +44 (0)1524 593746
Associate Dean for Research Email: r.pickup@lancaster.ac.uk
Faculty of Health and Medicine
(Division of Biomedical and Life Sciences)
Lancaster University
Lancaster
LA1 4YG

Thank you for taking the time to read this information.

Resources in the event of distress

Should you feel distressed either as a result of taking part, or in the future, the following resource may be of assistance: https://online.supportgroups.com/

Kind regards,

C Savelli

Carmen Savelli

Supplementary File 2 – Information Email #2: Results from Phase 1 and invitation to attend Webinar #2 (includes Phase 2 Participant Information)

To be sent by the INFOSAN Secretariat (infosan@who.int) on behalf of the researcher (cc: c.savelli@lancaster.ac.uk)

Health & Medicine | Lancaster University

Information Email #2

A mixed-methods exploration into the experience of members of the International Food Safety Authorities Network (INFOSAN): Phase 1 results and information about Phase 2

Dear INFOSAN Member,

My name is Carmen Savelli and in August 2018 I contacted you to let you know that I was conducting this study as a student in the PhD Public Health programme at Lancaster University, Lancaster, United Kingdom. You may also know me as one of the Technical Officers working as the INFOSAN Secretariat at the World Health Organization (WHO) in Geneva, Switzerland. I am writing again to inform you of the progress made so far, including results from Phase 1 and provide some additional information for participants in Phase 2.

What is the overall study about?
The purpose of this study is to interrogate INFOSAN in order to describe and explore the experiences of members and better understand the role of the network in mitigating the burden of foodborne illness around the world. The study will examine access to and usage of the INFOSAN Community Website, explore barriers and facilitators to active participation in INFOSAN, determine perceptions about the utility of INFOSAN to mitigate foodborne illness, and scrutinize if and how participation in this network creates value for members.

The study is designed in three phases: Phase 1 is complete and has examined access and usage patterns of the INFOSAN Community Website; Phase 2 will involve the completion of an online survey; and Phase 3 will involve individual interviews conducted online using WebEx. Taking part in one phase of the study does not require your participation in the other phases.

What are the results from Phase 1?

To be inserted

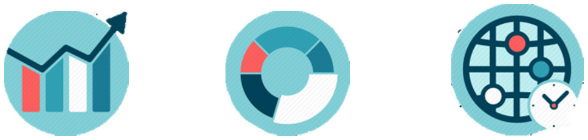

Why have I been approached about Phase 2 of this study?
You have been approached because the study requires information from registered INFOSAN members.

Do I have to take part in Phase 2?
No. It is completely up to you to decide whether or not you take part. Once the survey has been submitted, it will not be possible to opt out because survey responses are anonymous.

Continued on next page…

Continued from previous page…

What will I be asked to do if I take part?
Participation in Phase 2 of the study requires the completion of an online survey of approximately 30-45 minutes which will explore questions about your experience as an INFOSAN member. All INFOSAN Members will receive a separate invitation by email(Information Email #3) to provide consent and take the survey.

Will my data be Identifiable?
No. The information you provide will be reported anonymously. Quantitative data will be reported by region so that no country-specific details will be reported. The data collected for this study will be stored securely and only the researchers conducting this study will have access to this data:

- The files on the computer will be encrypted (that is no-one other than the researcher will be able to access them) and the computer itself password protected for the duration of the study
- At the end of the study, all related electronic information will be archived for 10 years in secure encrypted storage on the Lancaster University server. At the end of this period, they will be destroyed.

For further information about how Lancaster University processes personal data for research purposes and your data rights please visit our webpage: www.lancaster.ac.uk/research/data-protection

What will happen to the results?
The results will be summarised and reported in a PhD thesis and may be submitted for publication in relevant academic or professional conferences and journals or other media including books or websites, pending necessary clearance from WHO. Only those data collected with the expressed consent of participants is utilized and reported on for the purposes of this study. INFOSAN Members will also be invited to participate in a webinar after Phase 2 to learn about the results and next steps.

Are there any risks?
There are no risks anticipated with participating in this study. However, if you experience any distress following participation you are encouraged to inform the researcher and contact the resources provided at the end of this sheet. Neither participating nor abstaining will impact your future treatment as an INFOSAN member or the technical support provided to you or your country by the WHO.

Are there any benefits to taking part?
Although you may find participating interesting, there are no direct benefits in taking part. However, some participants may find it a positive experience to reflect on their role as an INFOSAN member as well as their contributions to a safer food supply, and to participate in research which they may feel is of relevance to them, their institution, their country or the global community.

Who has reviewed the project?
This study has been reviewed and approved by the Ethics Review Committee at the World Health Organization and the Faculty of Health and Medicine Research Ethics Committee at Lancaster University.

Where can I obtain further information about the study if I need it?
If you have any questions about the study, please contact the researcher:

Carmen Savelli
c.savelli@lancaster.ac.uk
+41 22 791 3234

Or his primary supervisor in the Department of Health Research at Lancaster University:

Dr Céu Mateus
c.mateus@lancaster.ac.uk
+44 1524 593182

Or attend the upcoming webinar:

Continued on next page…

Continued from previous page…

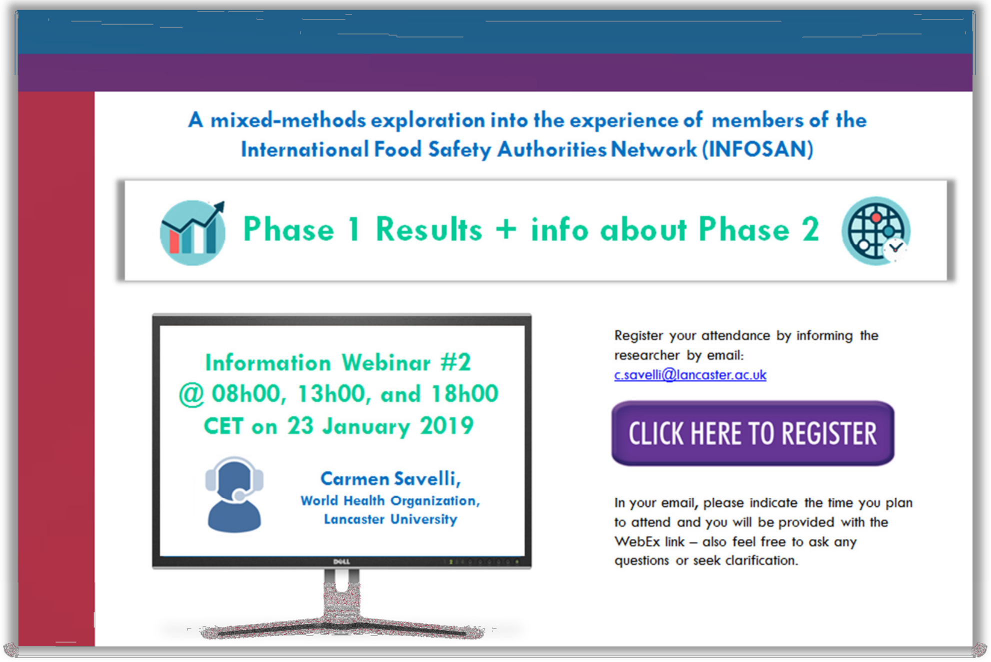

Complaints
If you wish to make a complaint or raise concerns about any aspect of this study and do not want to speak to the researcher or his supervisor, you can contact:

Professor Catherine Walshe
Head of the Division of Health Research
+44 (0)1524 510124
c.walshe@lancaster.ac.uk
Lancaster University
Lancaster
LA1 4YW

If you wish to speak to someone outside of the Public Health Doctorate Programme, you may also contact:

Professor Roger Pickup Tel: +44 (0)1524 593746
Associate Dean for Research Email: r.pickup@lancaster.ac.uk
Faculty of Health and Medicine
(Division of Biomedical and Life Sciences)
Lancaster University
Lancaster
LA1 4YG

Thank you for taking the time to read this information.

Resources in the event of distress
Should you feel distressed either as a result of taking part, or in the future, the following resource may be of assistance: https://online.supportgroups.com/

Kind regards,

Carmen Savelli

Supplementary File 3 – Information Email #3: Invitation to participate in Phase 2 including consent information and link to online survey

To be sent by the INFOSAN Secretariat (infosan@who.int) on behalf of the researcher (cc: c.savelli@lancaster.ac.uk)

Health & Medicine | Lancaster University

Information Email #3

A mixed-methods exploration into the experience of members of the International Food Safety Authorities Network (INFOSAN): Invitation to participate in Phase 2

Dear INFOSAN member,

You are invited to participate in Phase 2 of our study to explore the experiences of members of the International Food Safety Authorities Network (INFOSAN).

Phase 2 of the study involves completing an online survey that should take approximately 30-45 minutes of your time. Complete background details are available in the Participant Information Sheet, shared with you previously and also attached for ease of reference.

By proceeding to the survey you confirm that:

- ✓ You have read the information sheet and understand what is expected of you within this study;
- ✓ You confirm that you understand that any responses/information you give will remain anonymous;
- ✓ Your participation is voluntary;
- ✓ You consent for the information you provide to be discussed with my supervisor at Lancaster University;
- ✓ You consent to Lancaster University keeping the anonymised data for a period of 10 years after the study has finished;
- ✓ By clicking on the button below, you consent to taking part in the current study.

If you have any questions about this survey or other aspects of the study, please do not hesitate to contact me at any time: c.savelli@lancaster.ac.uk

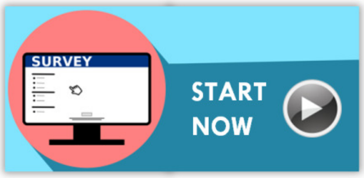

Kind regards,

Carmen Savelli

Supplementary File 4 – Expansion and Adaptation of Questionnaire

Contributor	Step
Verburg and Andriessen (2006)	1. Questionnaire in English from the Community Assessment Toolkit (CAT)
INFOSAN Advisory Group (Appendix 8)	2. Supplemental questions reviewed for content validity by a panel of experts consisting of Members of the INFOSAN Advisory Group
C. Savelli, Technical Officer, INFOSAN Secretariat, Department of Food Safety and Zoonoses (FOS)	3. Supplemental questions revised based on feedback prior to pilot testing
Regional Food Safety Officers and Advisors from FAO and WHO (Appendix 9)	4. Regional Food Safety Officers and Advisors from FAO and WHO pilot test the supplemental questions
C. Savelli	5. Revision prior to finalization in English
French translation: RD, WHO Intern, FOS<br>Spanish translation: RG, WHO Consultant, FOS	6. Forward translations of CAT questionnaire and supplemental questions into French and Spanish
**French Panel:**<br>▪ RC - PhD Candidate, formerly INFOSAN Secretariat at WHO<br>▪ CM – MD - WHO Implementation Research Scientist<br>▪ PBE - PhD, WHO, INFOSAN Secretariat<br>▪ RD - WHO Intern, FOS<br>**Spanish Panel**:<br>▪ EP – PhD, DVM, WHO Regional Food Safety Advisor<br>▪ SR – DVM, WHO Regional Food Safety Advisor<br>▪ JM – WHO, Technical Officer, FOS<br>▪ RG, WHO Consultant, FOS	7. Review of entire questionnaire by expert panels
C. Savelli	8. Revision as required
WHO Translation services	9. Backward translations into English
C. Savelli	10. Revision as required
WHO Interns	11. Pilot test
C. Savelli	12. Revision as required to finalized version

Supplementary File 5 – Information Email #4: Results from Phase 2 and invitation to attend Webinar #3 (includes Phase 3 Participant Information)

To be sent by the INFOSAN Secretariat (infosan@who.int) on behalf of the researcher (cc: c.savelli@lancaster.ac.uk)

Health & Medicine | Lancaster University

Information Email #4

A mixed-methods exploration into the experience of members of the International Food Safety Authorities Network (INFOSAN): Phase 2 results and information about Phase 3

Dear INFOSAN Member,

My name is Carmen Savelli and in August 2018 I first contacted you to let you know that I was conducting this study as a student in the PhD Public Health programme at Lancaster University, Lancaster, United Kingdom. You may also know me as one of the Technical Officers working as the INFOSAN Secretariat at the World Health Organization (WHO) in Geneva, Switzerland. I am writing again to inform you of the progress made so far, including results from Phase 2 and provide some additional information for participants in Phase 3.

What is the overall study about?
The purpose of this study is to interrogate INFOSAN in order to describe and explore the experiences of members and better understand the role of the network in mitigating the burden of foodborne illness around the world. The study will examine access to and usage of the INFOSAN Community Website, explore barriers and facilitators to active participation in INFOSAN, determine perceptions about the utility of INFOSAN to mitigate foodborne illness, and scrutinize if and how participation in this network creates value for members.

The study is designed in three phases: Phase 1 is complete and has examined access and usage patterns of the INFOSAN Community Website; Phase 2 is complete and involved the completion of an online survey by INFOSAN members; and Phase 3 will involve individual interviews conducted online using WebEx. Taking part in one phase of the study does not require your participation in the other phases.

What are the results from Phase2?

To be inserted

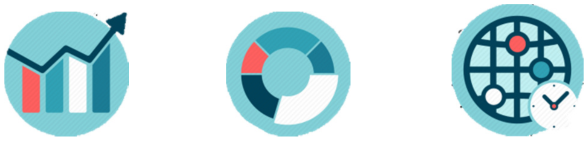

Why have I been approached about Phase 3 of this study?
You have been approached because the study requires information from registered INFOSAN members.

Do I have to take part in Phase 3?
No. It is completely up to you to decide whether or not you take part. Between 6 and 12 volunteers will be selected for Phase 3. If more than 2 people from a single WHO region indicate their interest in participating in Phase 3, the two people who have been members the longest will be selected and notified by email from the researcher, providing they are not from the same country.

Continued on next page…

Continued from previous page…

What will I be asked to do if I take part?

Participation in Phase 3 of the study will involve a one-on-one individual interview conducted online using WebEx with the researcher. The interview will last approximately 45-60 minutes and will explore in more detail your experiences as an INFOSAN Member.

To volunteer for Phase 3, please express your interest by contacting the researcher: c.savelli@lancaster.ac.uk

Will my data be Identifiable?

No. The information you provide will be reported anonymously. Any direct quotes from participants in Phase 3 will be reported anonymously. Quantitative data will be reported by region so that no country-specific details will be reported. The data collected for this study will be stored securely and only the researchers conducting this study will have access to this data:

- Audio recordings will be deleted once the thesis has been examined and accepted
- The files on the computer will be encrypted (that is no-one other than the researcher will be able to access them) and the computer itself password protected for the duration of the study
- At the end of the study, all related electronic information will be archived for 10 years in secure encrypted storage on the Lancaster University server. At the end of this period, they will be destroyed.
- The typed version of your interview will be made anonymous by removing any identifying information including your name. Anonymised direct quotations from your interview may be used in the reports or publications from the study, and neither your name nor your country will be attached to them.
- All your personal data will be confidential and will be kept separately from your interview responses. There are some limits to confidentiality: if what is said in the interview makes me think that you, or someone else, is at significant risk of harm, I will have to break confidentiality and speak to a member of staff about this. If possible, I will tell you if I have to do this. In addition, whilst every effort will be made, it is not possible for the researcher to ensure confidentiality of your participation in Phase 3 if you choose to connect to the researcher by WebEx on your work premises during the working day. If you volunteer to participate in Phase 3, you are encouraged to connect by WebEx in a private room to conduct your interview, where no one else can see your computer screen or hear your conversation.

For further information about how Lancaster University processes personal data for research purposes and your data rights please visit our webpage: www.lancaster.ac.uk/research/data-protection

What will happen to the results?

The results will be summarised and reported in a PhD thesis and may be submitted for publication in relevant academic or professional conferences and journals or other media including books or websites, pending necessary clearance from WHO. Only those data collected with the expressed consent of participants is utilized and reported on for the purposes of this study. INFOSAN Members will also be invited to participate in webinars after each phase to learn about the results and next steps.

Are there any risks?

There are no risks anticipated with participating in this study. However, if you experience any distress following participation you are encouraged to inform the researcher and contact the resources provided at the end of this sheet. Neither participating nor abstaining will impact your future treatment as an INFOSAN member or the technical support provided to you or your country by the WHO.

Are there any benefits to taking part?

Although you may find participating interesting, there are no direct benefits in taking part. However, some participants may find it a positive experience to reflect on their role as an INFOSAN member as well as their contributions to a safer food supply, and to participate in research which they may feel is of relevance to them, their institution, their country or the global community.

Who has reviewed the project?

This study has been reviewed and approved by the Ethics Review Committee at the World Health Organization and the Faculty of Health and Medicine Research Ethics Committee at Lancaster University.

Where can I obtain further information about the study if I need it?

If you have any questions about the study, please contact the researcher:

Carmen Savelli
c.savelli@lancaster.ac.uk
+41 22 791 3234

Or his supervisor in the Department of Health Research at Lancaster University:

Dr Céu Mateus
c.mateus@lancaster.ac.uk

Continued on next page…

Continued from previous page…

Or attend the upcoming webinar:

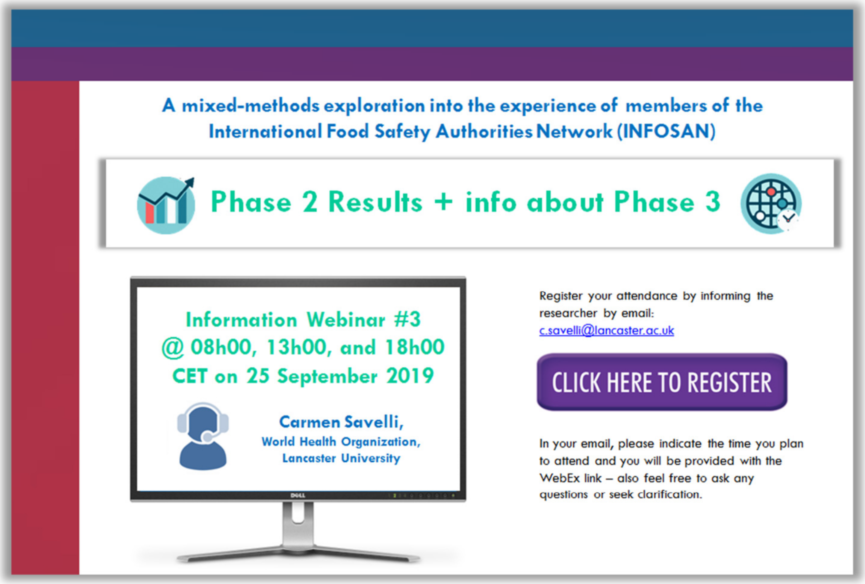

Complaints
If you wish to make a complaint or raise concerns about any aspect of this study and do not want to speak to the researcher or his supervisor, you can contact:

Professor Catherine Walshe
Head of the Division of Health Research
+44 (0)1524 510124
c.walshe@lancaster.ac.uk

Lancaster University
Lancaster
LA1 4YW

If you wish to speak to someone outside of the Public Health Doctorate Programme, you may also contact:

Professor Roger Pickup Tel: +44 (0)1524 593746
Associate Dean for Research Email: r.pickup@lancaster.ac.uk
Faculty of Health and Medicine
(Division of Biomedical and Life Sciences)
Lancaster University
Lancaster
LA1 4YG

Thank you for taking the time to read this information.

Resources in the event of distress
Should you feel distressed either as a result of taking part, or in the future, the following resource may be of assistance:
https://online.supportgroups.com/

Kind regards,

Carmen Savelli

Supplementary File 6 – Email to volunteers for Phase 3 that were not selected

To be sent from the researcher: c.savelli@lancaster.ac.uk

Health & Medicine | Lancaster University

Dear <<Name>>,

Many thanks for your interest in participating in Phase 3 of our study to explore the experiences of members of the International Food Safety Authorities Network (INFOSAN).

We have seen an overwhelmingly positive response to our call for volunteers to fill a limited number of interview slots. Unfortunately, due to resource restrictions including time restraints we will not be able to include you in this phase of the study.

Volunteers were selected on the basis of geographic location (maximum of two participants per WHO region) and length of membership to INFOSAN. If more than two members volunteered from the same region, the two who have been members of INFOSAN the longest were selected. This is what happened in your case.

Please be reminded that your active participation in the INFOSAN community is valued and appreciated and we hope you continue to engage in the important activities of the Network in the future.

If you have any additional information you wish to share regarding your experience as an INFOSAN member, you are always welcome to contact the INFOSAN Secretariat at WHO directly (infosan@who.int) outside of the context of this study.

Kind regards,

CSavelli

Carmen Savelli

Supplementary File 7 – Consent form for volunteers participating in Phase 3

To be sent from the researcher: c.savelli@lancaster.ac.uk

Health & Medicine | Lancaster University

A mixed-methods exploration into the experience of members of the International Food Safety Authorities Network (INFOSAN): Phase 3 consent form

Many thanks for your interest in participating in Phase 3 of our study to explore the experiences of members of the International Food Safety Authorities Network (INFOSAN).

Consent Form

Before you consent to participating in the study we ask that you re-read the participant information (contained in Information Email #4 and attached for ease of reference) and mark each box below with your initials if you agree. If you have any questions or queries before signing the consent form please contact the researcher, Carmen Savelli (c.savelli@lancaster.ac.uk or +41 (0) 79 9456 320).

Initial here

1. I confirm that I have read the participant information and fully understand what is expected of me within this study ☐
2. I confirm that I have had the opportunity to ask any questions and to have them answered. ☐
3. I understand that my interview will be audio recorded and then made into an anonymised written transcript. ☐
4. I understand that audio recordings will be kept until the research project has been examined. ☐
5. I understand that my participation is voluntary and that I am free to withdraw at any time without giving any reason. ☐
6. I understand that once my data have been anonymised and incorporated into themes it might not be possible for it to be withdrawn, though every attempt will be made to extract my data, up to the point of publication. ☐
7. I understand that the information from my interview will be pooled with other participants' responses, anonymised and may be published. ☐
8. I consent to information and quotations from my interview being used in reports, conferences and training events. ☐
9. I understand that the researcher will discuss data with their supervisor as needed. ☐
10. I understand that any information I give will remain confidential and anonymous unless it is thought that there is a risk of harm to myself or others, in which case the principal investigator will need to share this information with their research supervisor. ☐ ☐
11. I consent to Lancaster University keeping written transcriptions of the interview for 10 years after the study has finished. ☐
12. I consent to take part in the above study. ☐

Name of Participant__________________ Signature____________________ Date ___________

Name of Researcher __________________Signature ____________________Date ___________

Supplementary File 8 – Interview Guide for Phase 3

The semi-structured format of the interviews will be conversational in style, allowing the researcher and participant to engage in a dialogue where questions can be modified depending on responses. However, the following questions can serve to start discussions within the bounds of the four themes:

Theme 1) Use of the ICW to support network activities

Preface: The INFOSAN Community Website was launched in 2012 and all members of INFOSAN are registered on there. This online portal is the gateway through which all members can communicate with each other and with the INFOSAN Secretariat.

Questions about experiences:

- How often do you access the INFOSAN Community Website?
- What do you normally use it for?
- Do you think it is supporting the activities of INFOSAN?
- Do you think it could be improved and how?
- Do you think the INFOSAN Secretariat should be posting more frequently on the website?
- What kind of information do you think the INFOSAN Secretariat should be posting there?
- How do you think we might be able to get more members engaged on the website, e.g. involved in discussions, sharing documents, etc.?
- What new features would you like to see?
- Do you think a mobile version of the website would improve participation?

Theme 2) Barriers and facilitators to active participation in INFOSAN

Preface: INFOSAN has an important mandate, to communicate between national government authorities around the world to keep the global food supply safe. However, we know that some members are more active than others. Let's discuss some of the potential barriers to and facilitators to participation.

Questions about experiences:

- Is limited capacity in your country a barrier to participation (i.e. Limited capacity/infrastructure dedicated to addressing food safety; insufficient funds; human resources/expertise; national food control system underdeveloped) and if so, how?
- Is lack of training a barrier (Laboratory analysis; food safety risk assessment; outbreak investigation), and if so, how?
- Does a lack of national food safety standards pose a barrier, and if so, how?
- What is coordination between national authorities like at the national level?
- Does a lack of coordination pose a barrier, and if so, how?
- Are their legal constraints that bar your participation in INFOSAN (e.g. legal implications hinder prompt information sharing; lack of food safety legislation; lack of cooperation from industry, etc.), and if so, how?
- Is food safety a priority in your country? If not, does this pose a barrier, and if so, how?

For peer review only

- Are their perceptions that participating in INFOSAN may have a negative impact on the economy (i.e. through damages to trade or tourism sectors)?
- Is the mandate of INFOSAN clear? If not, how could it be clarified?
- If more members understood their roles and responsibilities better, would that facilitate participations? What would be the best way to do this?
- Have you ever attended any INFOSAN training either online or in person? What did you think about the training? How could the training be improved?
- Do you think language barriers restrict active participation in INFOSAN , and if yes, how so?
- Do you have concerns over confidentiality that limit what you share with INFOSAN?
- How could these concerns be addressed to help facilitate your improved participation?
- Are there any obvious weaknesses of INFOSAN, which if rectified could significantly improve the functioning and thereby the impact of INFOSAN?

Theme 3) Perceptions of network achievements and attainment of objectives (including reducing foodborne illness)

Preface: INFOSAN aims to prevent the international spread of contaminated food and foodborne disease and strengthen food safety systems globally, by: 1) Promoting the rapid exchange of information during food safety incidents/emergencies; 2) Sharing information on important food safety issues of global interest; 3) Promoting partnership and collaboration between countries; and 4) Helping countries strengthen their capacity to manage food safety risks "from farm to table".

Questions about experiences:

- How useful has INFOSAN been in promoting the rapid exchange of information during food safety incidents/emergencies?
- Have you been involved in such exchanges? What was that like? Did you get the information you needed quickly enough to respond and implement risk management measures?
- What have your experiences been like when dealing with the INFOSAN Secretariat?
- Does INFOSAN share information on important food safety issues of global interest that you have found useful?
- Have you shared information with other network members?
- What have your experiences been like in terms of using information you received through INFOSAN or sharing information with others in the network?
- Has INFOSAN promoted partnership and collaboration between countries and networks? If yes, how so? Was it useful?
- What has been your experience when interacting with other INFOSAN members during a food safety emergency?
- Has INFOSAN helped countries strengthen their capacity to manage food safety risks "from farm to table"? If yes, how so?
- How could INFOSAN improve the way it functions in order to better achieve its objectives?

Relevance:

- To what extent are the objectives of INFOSAN still valid?

For peer review only

- Are the activities and outputs of INFOSAN consistent with the overall goal and the attainment of its objectives?
- Are the activities and outputs of INFOSAN consistent with the intended impacts and effects?

Effectiveness:

- To what extent have the objectives been achieved or are likely to be achieved?
- What are the major factors influencing the achievement or non-achievement of the objectives?

Efficiency:

- Were INFOSAN activities cost-efficient?
- Were objectives achieved in a timely manner?
- Were INFOSAN activities delivered in the most efficient way compared to alternatives?

Impact:

- What has been the biggest impact that INFOSAN has made?
- What real difference has INFOSAN made to members? To the safety of the global food supply? To the improvement of global food safety systems?
- How have populations' health been affected?

Sustainability:

- What are the major factors which influence the achievement or non-achievement of the sustainability of INFOSAN?

Theme 4) Assessing value of INFOSAN

Preface: INFOSAN has been in operation since 2004, and this study is the first of its kind to try and determine if the network has value to members in a robust and rigorous way.

Questions about experiences:

- What has participation in INFOSAN been a valuable experience for you? How so?
- Have you been able to connect with other food safety professionals to exchange information related to food safety?
- Has information received through INFOSAN led you to implement risk management measures in your country to prevent foodborne illness? Can you tell me about some specific events?
- Do you believe that participation in INFOSAN has reduced foodborne illness in your country? How so? How do you think this could be measured?
- Do you think participation in INFOSAN will reduce foodborne illness in your country the future? Globally?
- Does the utilization of the INFOSAN Community Website add value to the network activities?
- Do you feel like you are part of a global community? Is that something you value?

Is there anything else about your experience as an INFOSAN Member that you would like to share with me at this time?

For peer review only

Supplementary File 9 – Information Email #5 – Results from Phase 3 and study conclusion (including invitation to attend Webinar #4)

To be sent by the INFOSAN Secretariat (infosan@who.int) on behalf of the researcher (cc: c.savelli@lancaster.ac.uk)

Health & Medicine | Lancaster University

Information Email #5

A mixed-methods exploration into the experience of members of the International Food Safety Authorities Network (INFOSAN): Phase 3 results and study conclusion

Dear INFOSAN Member,

My name is Carmen Savelli and in May 2018 I first contacted you to let you know that I was conducting this study as a student in the PhD Public Health programme at Lancaster University, Lancaster, United Kingdom. You may also know me as one of the Technical Officers working as the INFOSAN Secretariat at the World Health Organization (WHO) in Geneva, Switzerland. The study has now concluded.

What was the overall study about?
The purpose of this study is to interrogate INFOSAN in order to describe and explore the experiences of members and better understand the role of the network in mitigating the burden of foodborne illness around the world. The study will examine access to and usage of the INFOSAN Community Website, explore barriers and facilitators to active participation in INFOSAN, determine perceptions about the utility of INFOSAN to mitigate foodborne illness, and scrutinize if and how participation in this network creates value for members.

The study was designed in three phases: Phase 1 has examined access and usage patterns of the INFOSAN Community Website; Phase 2 involved the completion of an online survey by INFOSAN members; and Phase 3 involved individual interviews conducted online using WebEx.

What are the results from Phase 3?

To be inserted

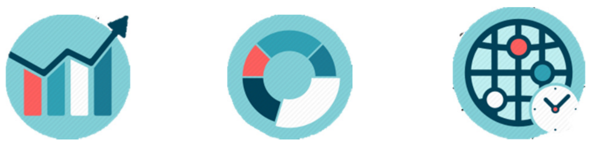

Continued on next page…

Continued from previous page…

How can I find out more about the results from Phase 3 and the overall study conclusion?
Attend the upcoming Webinar:

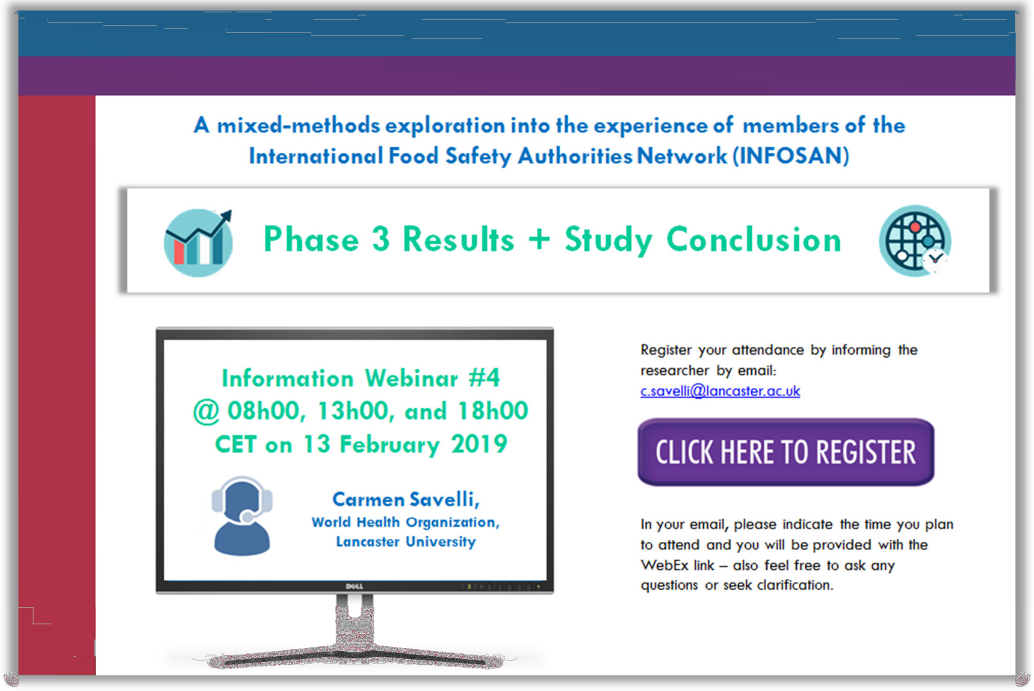

What are the next steps?

The results of this study have been shared with the INFOSAN Secretariat and other senior staff at the World Health Organization for their consideration. Improving the capacity of INFOSAN members to better prevent, detect, and respond to food safety emergencies is a priority.

Any forthcoming publications linked to the study results will be announced by email to the INFOSAN members by the INFOSAN Secretariat.

If you have any additional feedback about this study you are welcome to contact the researcher (c.savelli@lancaster.ac.uk).

If you have other feedback about INFOSAN in general that you wish to share with WHO, please contact the INFOSAN Secretariat directly (infosan@who.int).

Many thanks to all INFOSAN members who contributed time and data to this study. Your participation has been invaluable and is sincerely appreciated.

Kind regards,

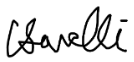

Carmen Savelli
